# Supplementary material for: Fam134c and Fam134b shape axonal endoplasmic reticulum architecture in vivo
Source: EMBO Rep. 2024 Jul 22;25(8):25. doi: 10.1038/s44319-024-00213-7 (PMC11316074; doi:10.1038/s44319-024-00213-7)
Supplement: Supplementary file 1 — Appendix [file 44319_2024_213_MOESM1_ESM.pdf]

Appendix

Fam134c and Fam134b shape axonal endoplasmic reticulum architecture in vivo.

Francescopaolo Iavarone[1*#], Marta Zaninello[2,3,4*], Michela Perrone[5], Mariagrazia Monaco[1], Esther Barth[2,3], Felix Gaedke[3], Maria Teresa Pizzo[1], Giorgia Di Lorenzo[1], Vincenzo Desiderio[6], Eduardo Sommella[7], Fabrizio Merciai[7], Emanuela Salviati[7], Pietro Campiglia[7], Livio Luongo[5], Elvira De Leonibus[1,8#], Elena Rugarli[2,3,4#], Carmine Settembre[1,9#]

[1] Telethon Institute of Genetics and Medicine (TIGEM), Pozzuoli, Italy.
[2] Institute for Genetics, Faculty of Mathematics and Natural Sciences, University of Cologne, Germany.
[3]Cologne Excellence Cluster on Cellular Stress Responses in Aging-Associated Diseases, University of Cologne, Germany.
[4]Center for Molecular Medicine, University of Cologne, Cologne, Germany.
[5]Department of Experimental Medicine, Division of Pharmacology, University of Campania "L. Vanvitelli", Naples, Italy.
[6] Department of Experimental Medicine, University of Campania "Luigi Vanvitelli", Via L. Armanni 5, 80138 Naples, Italy.
[7] Department of Pharmacy, University of Salerno, Via Giovanni Paolo II, 132, Fisciano, SA, 84084, Italy
[8] Institute of Biochemistry and Cell Biology, Monterotondo (Rome), Italy.
[9] Department of Clinical Medicine and Surgery, Federico II University, Naples, Italy.

[*] Equal contribution.
[#] Corresponding authors.
f.iavarone@tigem.it
deleonibus@tigem.it
elena.rugarli@uni-koeln.de
settembre@tigem.it

Table of contents:

Appendix Figure S1 ……………………………………………………………………..……..2
Appendix Figure S2 ……………………………………………………………………..………..3
Appendix Figure S3 ……………………………………………………………………..………..4
Appendix Figure S4 ………….……………………………………………………………………..6
Appendix Figure S5 ………….……………………………………………………………………..7
Appendix Figure S6 ………….……………………………………………………………………..8
Appendix Figure S7 ………….……………………………………………………………………..9
Appendix Figure S8 ………….……………………………………………………………………..11

Appendix Figure S1

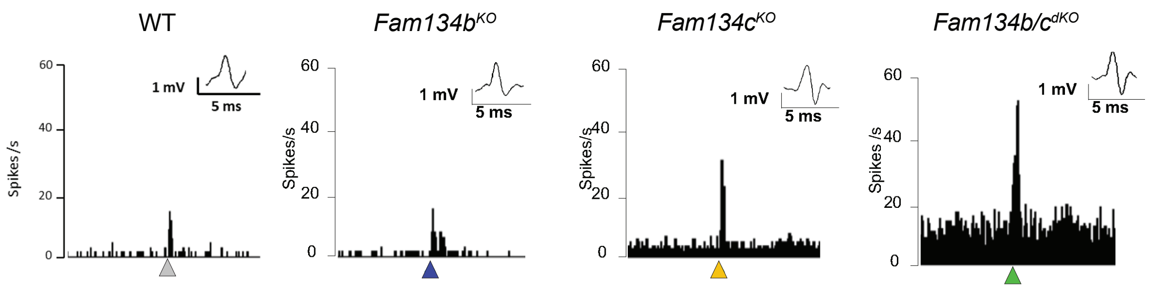

Appendix Figure S1

Representative ratemeters showing spontaneous activity, evoked activity and duration of the evoked activity. Activity was evoked by pain stimulation in WT, *Fam134b*^{KO}, and *Fam134c*^{KO}, and *Fam134b/c*^{dKO} spinal cord nociceptive neurons. The arrowheads at the bottom of the plots show the time at which nociceptive stimulation was performed in the animal's hind paw.

Appendix Figure S2

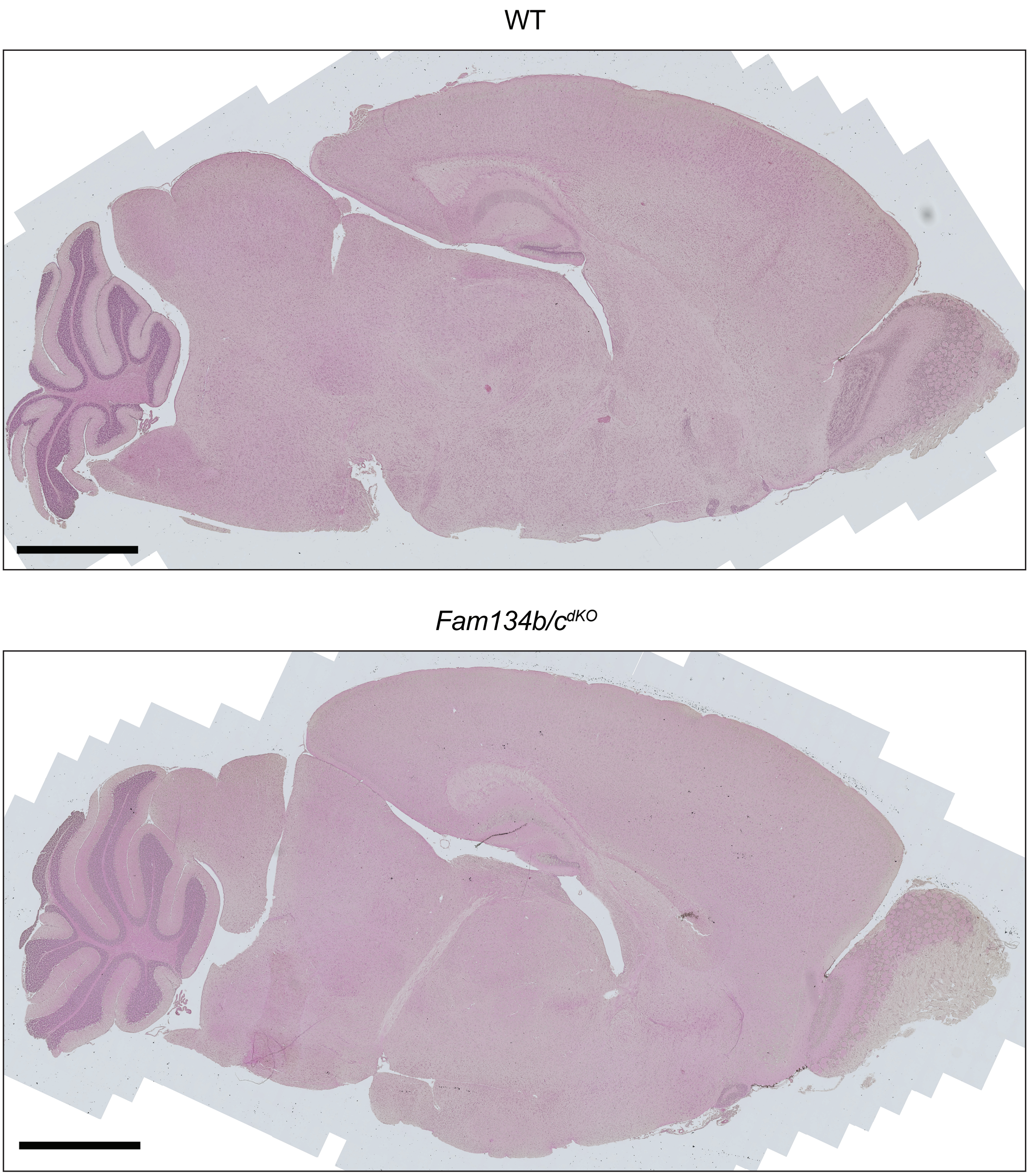

Appendix Figure S2

Representative Haematoxylin & Eosin staining of sagittal brain section from WT and *Fam134b/c*^{dKO} mice aged 4 weeks. Scale bar, 1mm.

Appendix Figure S3

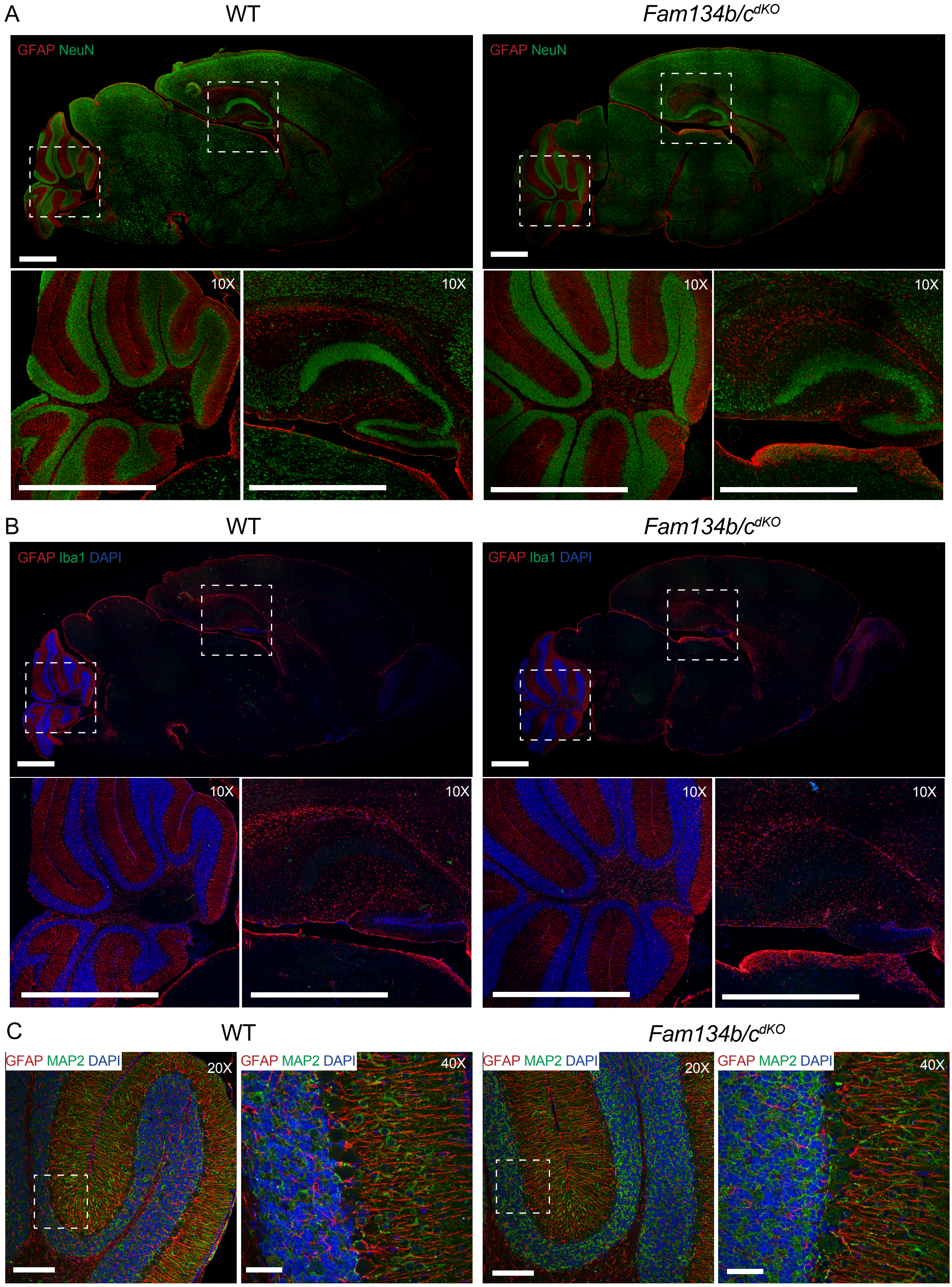

Appendix Figure S3

(A-B) Representative immunofluorescence staining of GFAP (red) and NeuN (green) (A) or Iba1 (green) (B) in sagittal brain sections from WT and *Fam134b/c*^{dKO} mice aged 4 weeks. Scale bar, 1mm.
(C) Representative immunofluorescence staining of GFAP (red) and MAP2 (green) in sagittal brain sections from WT and *Fam134b/c*^{dKO} mice aged 4 weeks. Scale bar, 150µm (50µm in the insets).

Appendix Figure S4

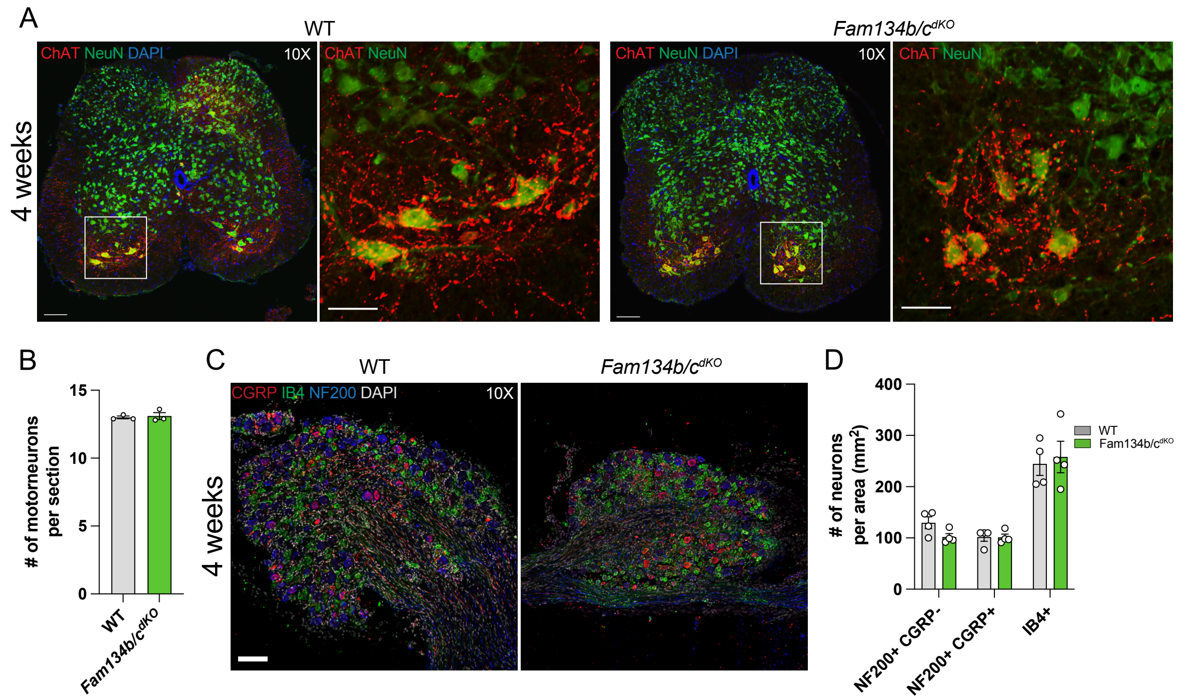

Appendix Figure S4

(A) Representative immunofluorescence staining of ChAT (red) and NeuN (green) of L4 spinal cord sections from WT and *Fam134b/c*^{dKO} mice aged 4 weeks. Scale bar, 100 µm or 50 µm in the insets. (B) Quantification of motoneuron number per section identified as ChAT+ cells in the ventral horn area. n = 3 animals/group. Data represent mean ± SEM. (C) Representative immunofluorescence staining of NF200 (blue), CGRP (red), IB4 (green) of lumbar DRG sections from WT and *Fam134b/c*^{dKO} mice aged 4 weeks. Scale bar, 100 µm. (D) Quantification showing the number of sensory neurons per area (mm^2). n = 4 animals/group. Data represent mean ± SEM.

Appendix Figure S5

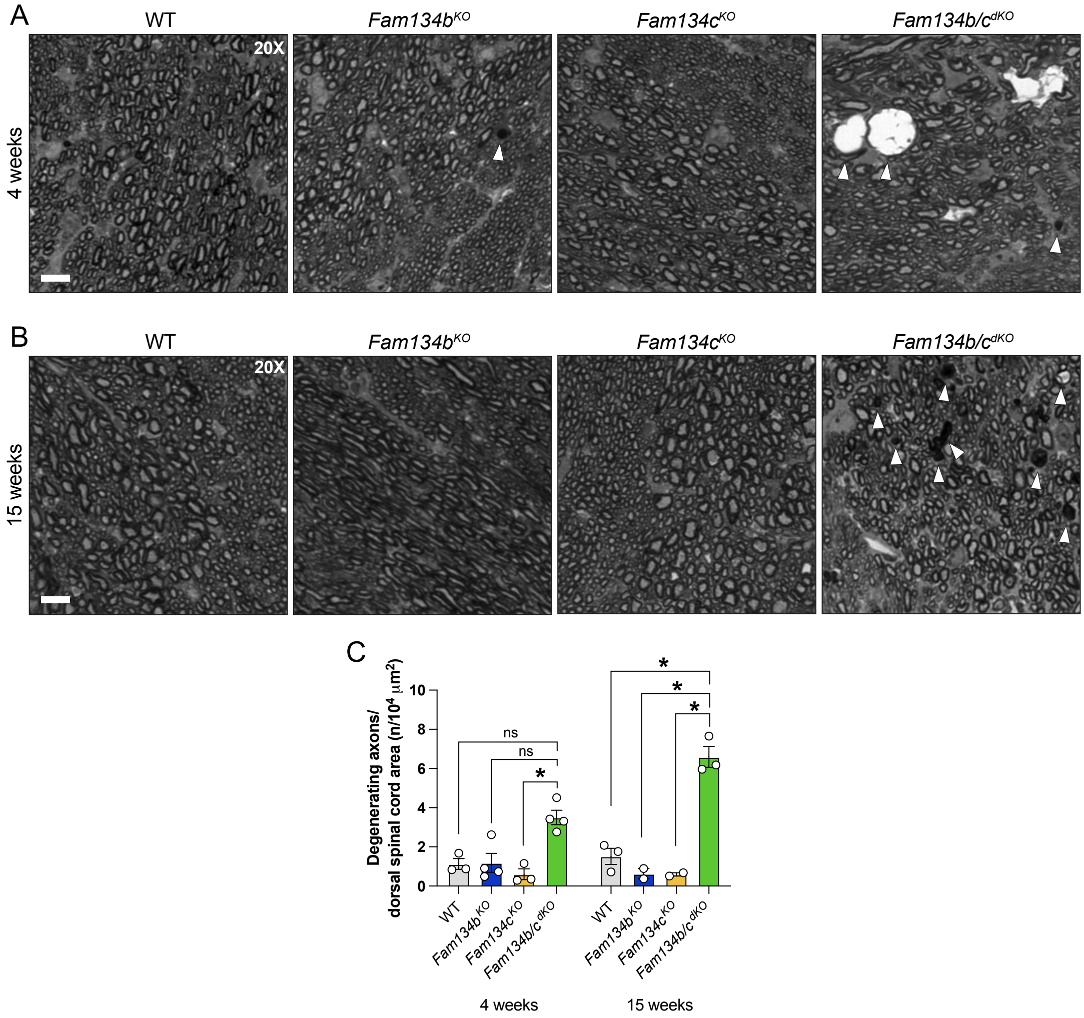

Appendix Figure S5

(A, B) Representative toluidine staining of dorsal spinal cord of WT, *Fam134b*^{KO}, *Fam134c*^{KO} and *Fam134b/c*^{dKO} mice aged 4 (A) and 15 (B) weeks. White arrowheads indicate degenerating axons. Scale bars, 20 µm. (C) *Fam134b/c*^{dKO} mice show a progressive degeneration of axons in the dorsal spinal cord from 4 to 15 weeks of age respect with WT, *Fam134b*^{KO} and *Fam134c*^{KO} mice. Statistical significance was determined by One-way ANOVA ($p=0.0311$) followed by Dunnett's T3 multiple comparisons test. Data represent mean ± SEM. $n \geq 3$ animals/group. ns $p > 0.05$, *$p < 0.05$.

Appendix Figure S6

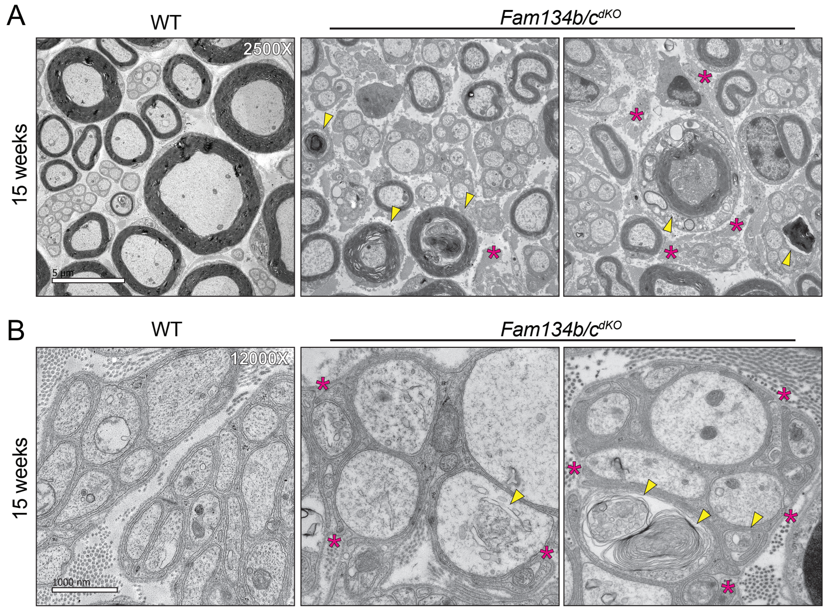

Appendix Figure S6

(A) Representative electron micrographs showing degenerating axons, accumulating organelles, and cytoskeletal components (yellow arrows) in sciatic nerves of WT and *Fam134b/c*^{dKO} mice aged 15 weeks. Collagen areas are indicated by magenta asterisks. Scale bar, 5 µm. (B) Unmyelinated axons exhibit accumulation of material (yellow arrows) and incomplete wrapping by Schwann cells (magenta asterisks) in tibial nerves of *Fam134b/c*^{dKO} aged 15 weeks. Scale bar, 1 µm.

Appendix Figure S7

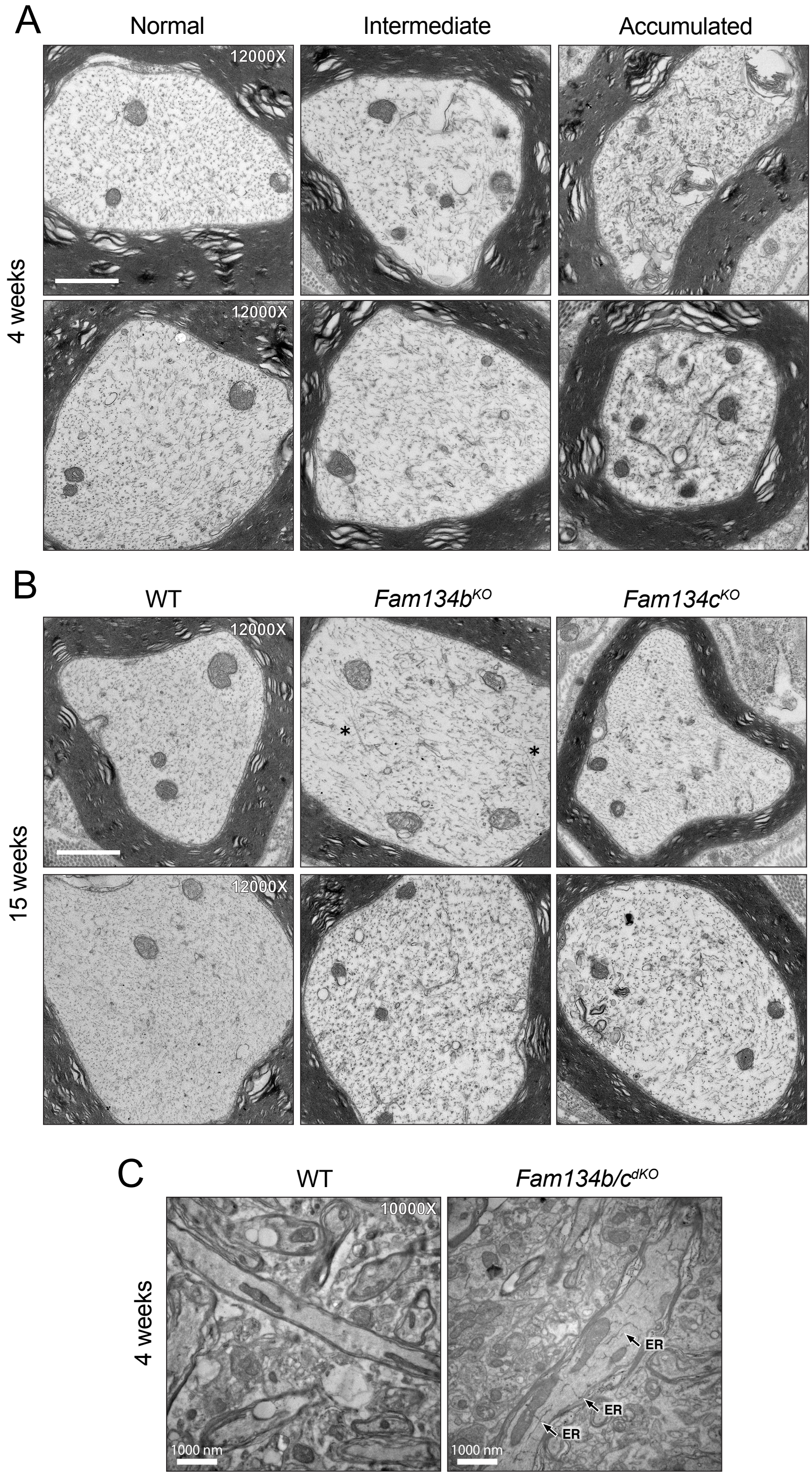

Appendix Figure S7

(A) Representative electron micrographs of normal, intermediate and accumulated ER in tibial nerves axons from mice aged 4 weeks. Scale bar, 1 µm. (B) Representative electron micrographs of ER in tibial nerves axons from WT, *Fam134b*^{KO}, and *Fam134c*^{KO} mice aged 15 weeks. Alterations of the cytoskeleton are highlighted with asterisks. Scale bar, 1 µm. (C) Representative electron micrographs of longitudinal axon from WT and *Fam134b/c*^{dKO} ventral spinal cord at 4 weeks, showing ER accumulation and disorganization in *Fam134b/c*^{dKO} axon. Scale bar, 1 µm.

Appendix Figure S8

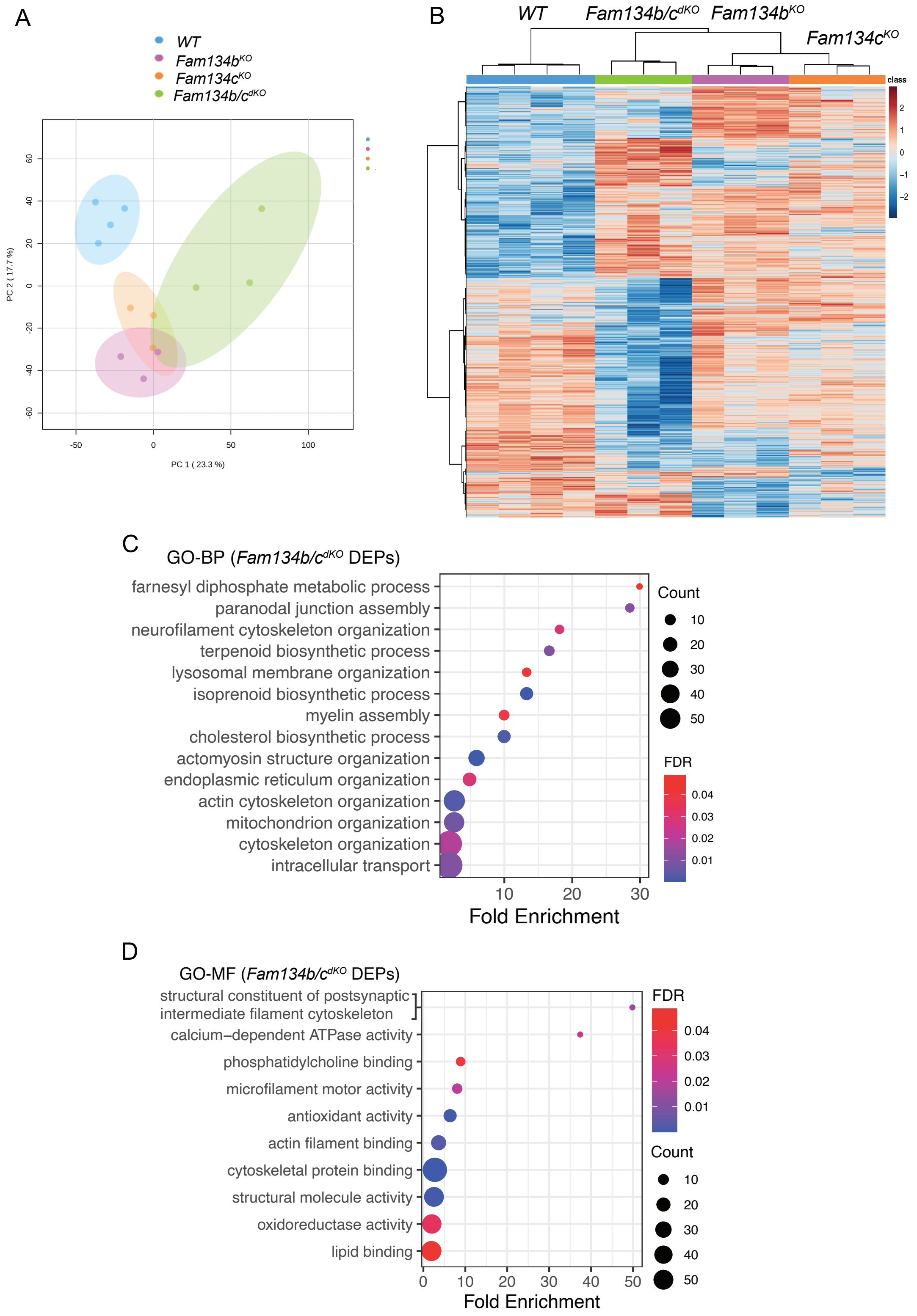

Appendix Figure S8

(A) Principal component analysis for WT, *Fam134b*^{KO}, *Fam134c*^{KO} and *Fam134b/c*^{dKO} sciatic nerve proteome. (B) Heatmap and 2-dimension hierarchical clustering of protein expression among WT, *Fam134b*^{KO}, *Fam134c*^{KO} and *Fam134b/c*^{dKO} group. The log2 relative protein expression scale is depicted on the top right. (C-D) Most relevant terms from Gene Ontology Biological Process (GO-BP) (C) or Gene Ontology Molecular Function (GO-MF) (D) of total *Fam134b/c*^{dKO} DEPs are represented by dot plots indicating FDR, DEP count and fold enrichment. $n \geq 3$ animals/group. FDR < 0.05.
