# Supplementary material for: Fam134c and Fam134b shape axonal endoplasmic reticulum architecture in vivo
Source: EMBO Rep. 2024 Jul 22;25(8):25. doi: 10.1038/s44319-024-00213-7 (PMC11316074; doi:10.1038/s44319-024-00213-7)
Supplement: Supplementary file 8 — Movie EV4 [file 44319_2024_213_MOESM8_ESM.zip › Movie EV4 - Legend.rtf]

Video showing 3D reconstruction of Fam134b/c dKO neuromuscular junction at 15 weeks showed in Fig. 3G.
