# Supplementary material for: Fam134c and Fam134b shape axonal endoplasmic reticulum architecture in vivo
Source: EMBO Rep. 2024 Jul 22;25(8):25. doi: 10.1038/s44319-024-00213-7 (PMC11316074; doi:10.1038/s44319-024-00213-7)
Supplement: Supplementary file 13 — Movie EV9 [file 44319_2024_213_MOESM13_ESM.zip › Movie EV9 - Legend.rtf]

Video showing electron microscopy tomograms of sciatic axon from WT 4-wk-old mice, used to perform the 3-D reconstruction showed in Fig. 4E.
