# Supplementary figures and images for: Fam134c and Fam134b shape axonal endoplasmic reticulum architecture in vivo
Source: EMBO Rep. 2024 Jul 22;25(8):25. doi: 10.1038/s44319-024-00213-7 (PMC11316074; doi:10.1038/s44319-024-00213-7)

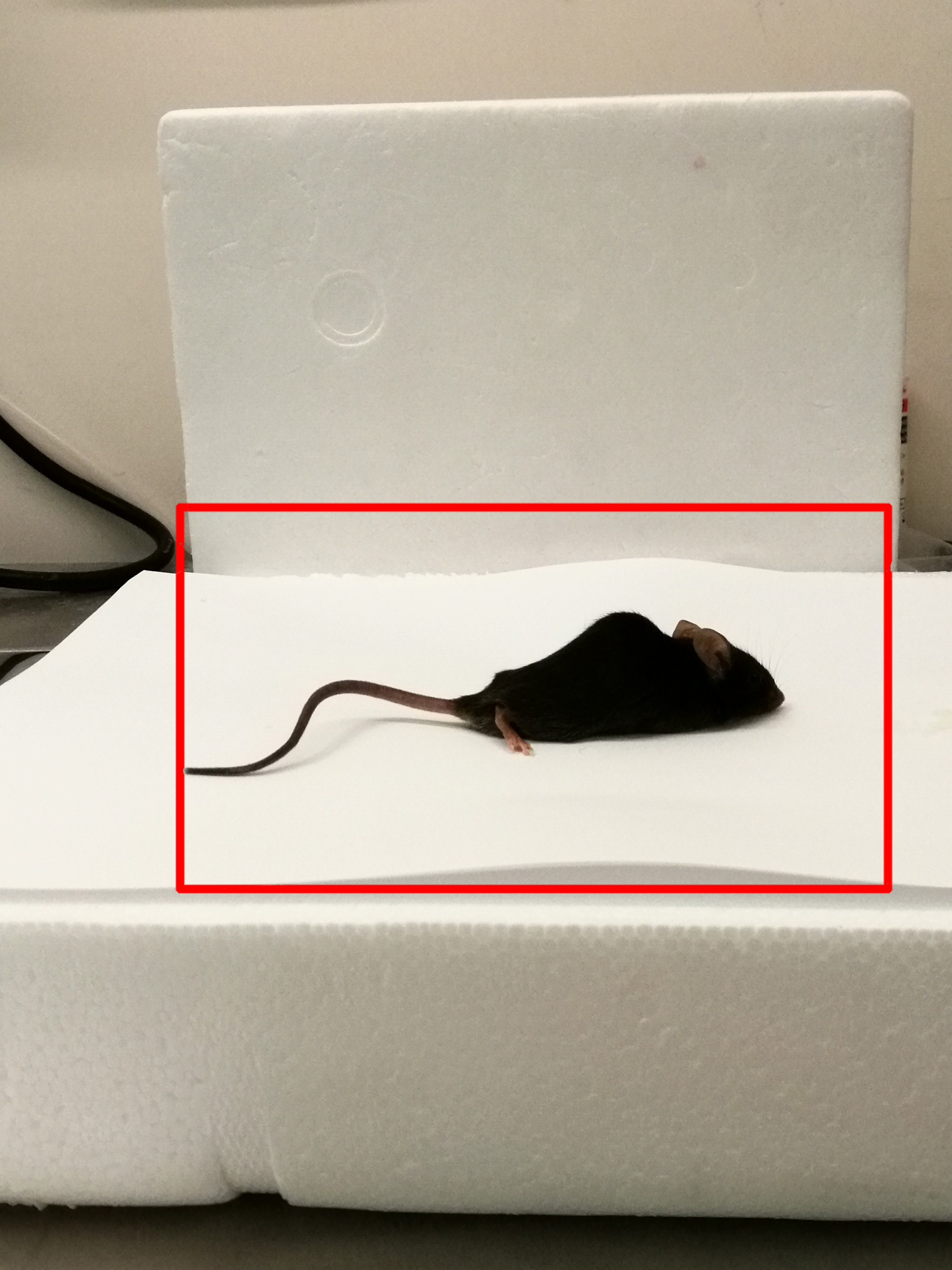

Supplement: Supplementary file 17 — Source data Fig. 1 [file 44319_2024_213_MOESM17_ESM.zip › Figure 1/1F/IMG_20230929_104408.jpg]

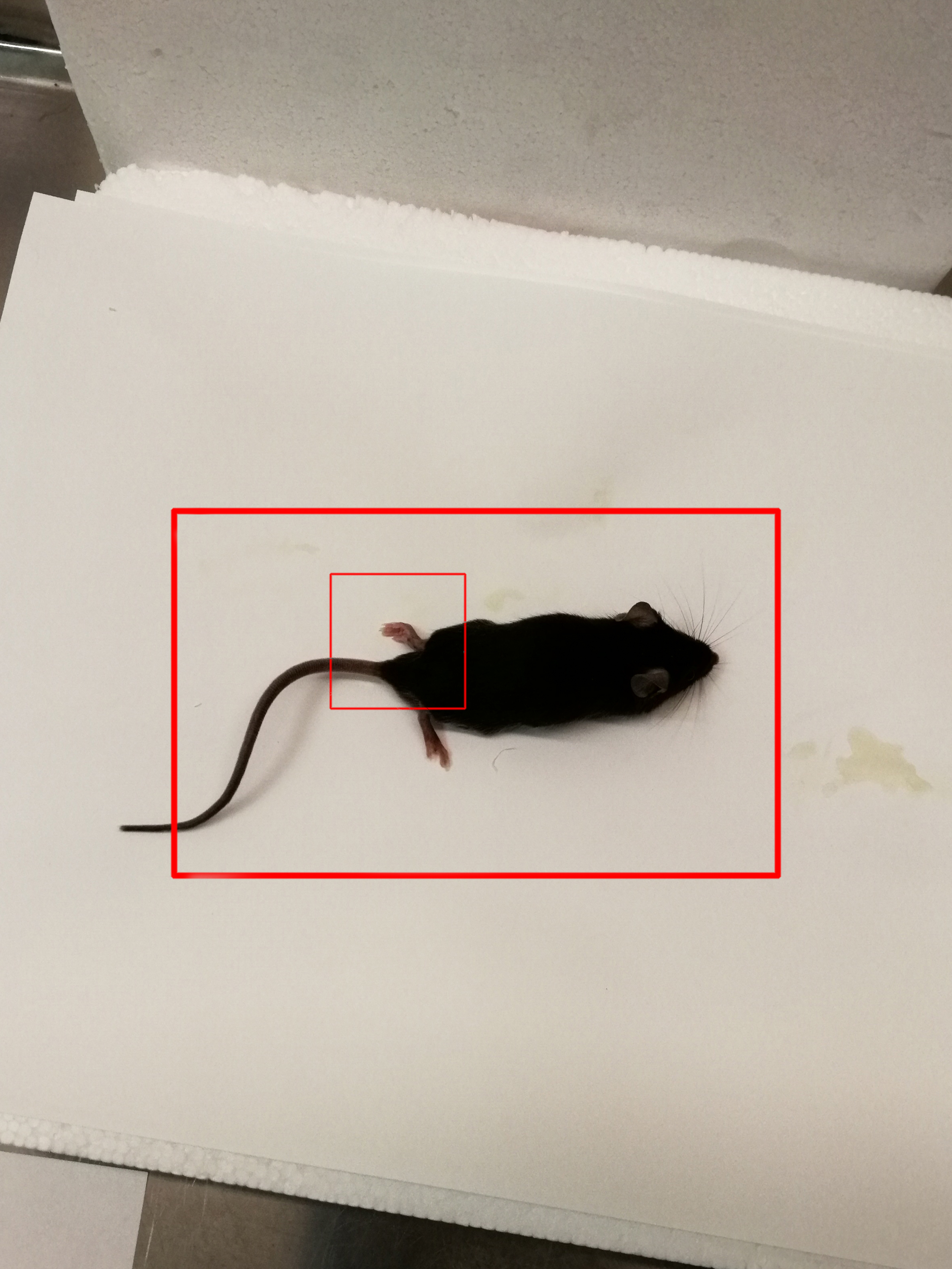

Supplement: Supplementary file 17 — Source data Fig. 1 [file 44319_2024_213_MOESM17_ESM.zip › Figure 1/1F/IMG_20230929_104404.jpg]

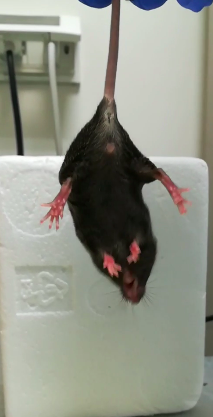

Supplement: Supplementary file 17 — Source data Fig. 1 [file 44319_2024_213_MOESM17_ESM.zip › Figure 1/1G/WT.png]

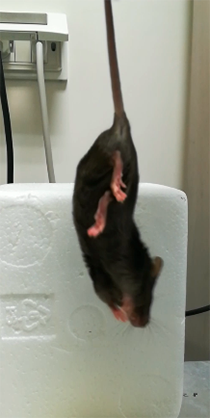

Supplement: Supplementary file 17 — Source data Fig. 1 [file 44319_2024_213_MOESM17_ESM.zip › Figure 1/1G/BC dKO.png]

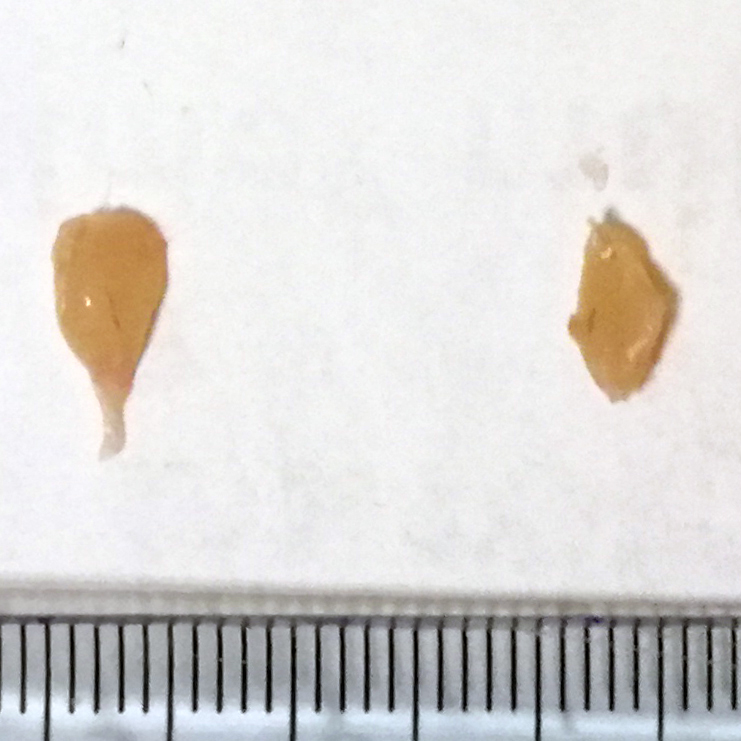

Supplement: Supplementary file 17 — Source data Fig. 1 [file 44319_2024_213_MOESM17_ESM.zip › Figure 1/1B/CKO_Gastro_Tricep.jpg]

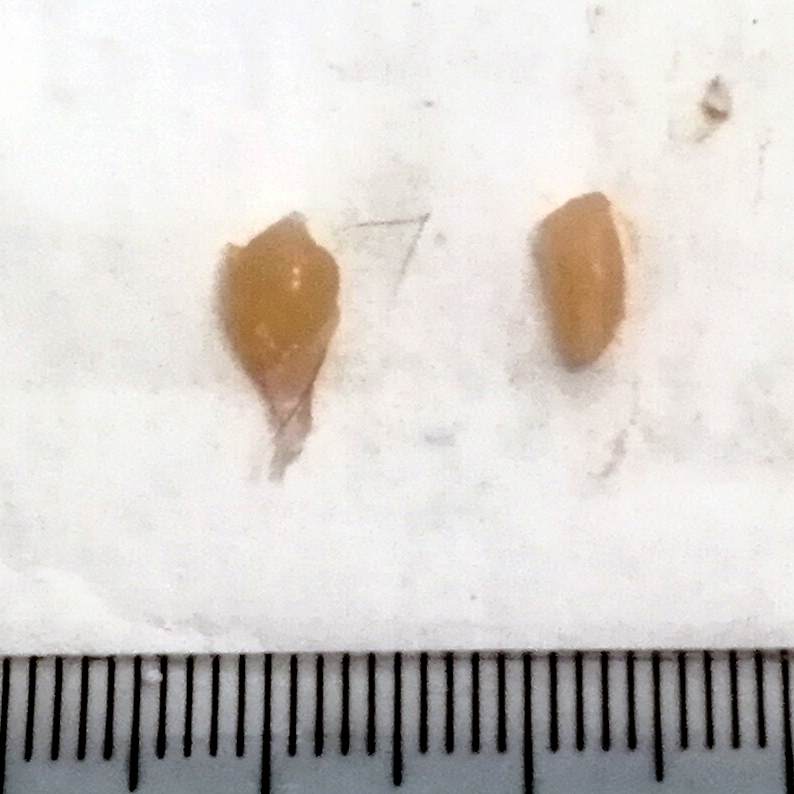

Supplement: Supplementary file 17 — Source data Fig. 1 [file 44319_2024_213_MOESM17_ESM.zip › Figure 1/1B/BKO_Gastro_Tricep.jpg]

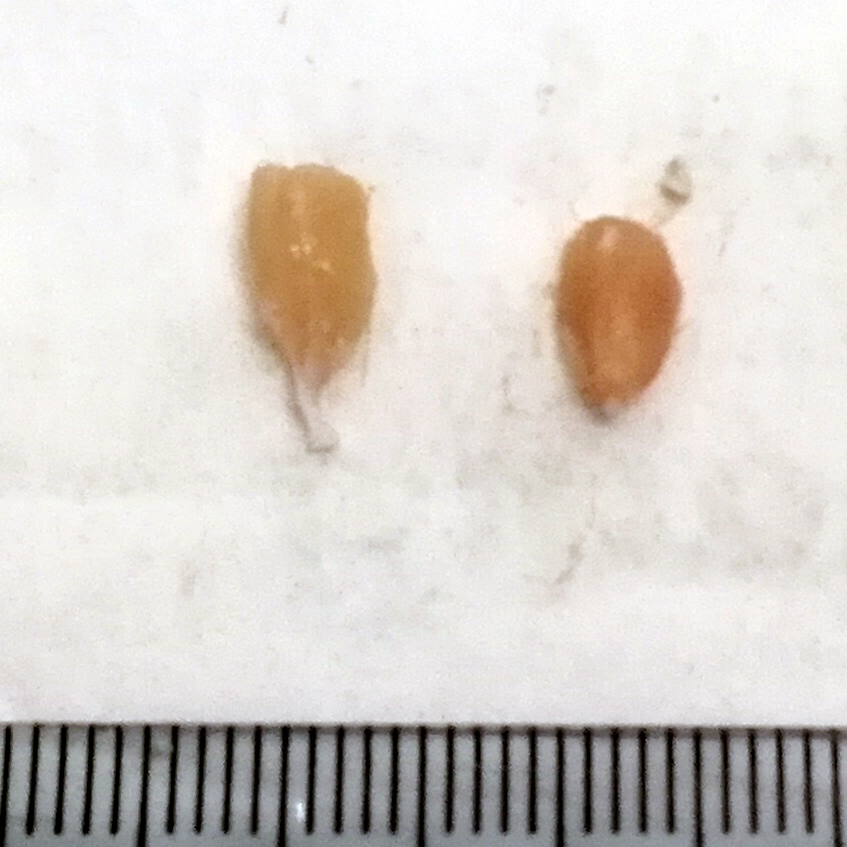

Supplement: Supplementary file 17 — Source data Fig. 1 [file 44319_2024_213_MOESM17_ESM.zip › Figure 1/1B/WT_Gastro_Tricep.jpg]

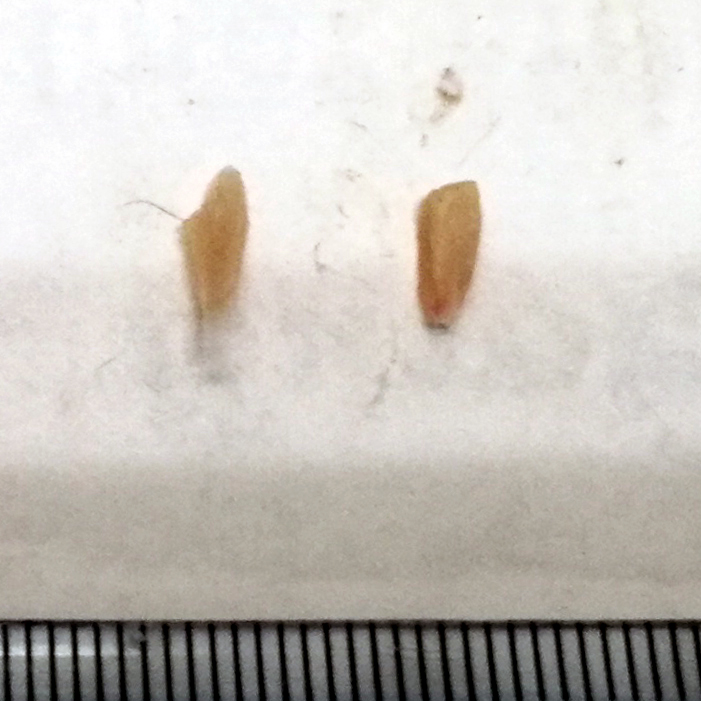

Supplement: Supplementary file 17 — Source data Fig. 1 [file 44319_2024_213_MOESM17_ESM.zip › Figure 1/1B/BCdKO_Gastro_Tricep.jpg]

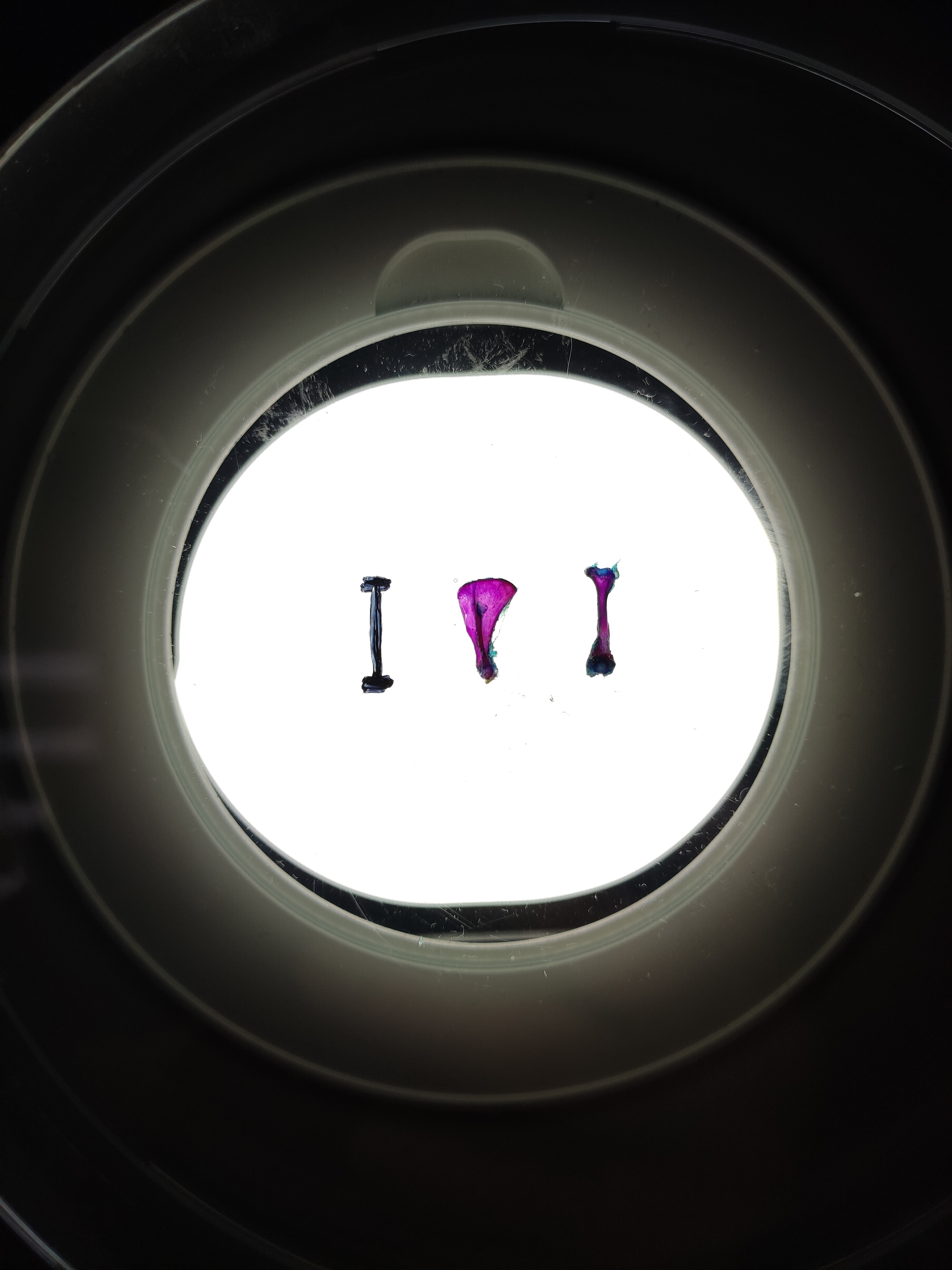

Supplement: Supplementary file 17 — Source data Fig. 1 [file 44319_2024_213_MOESM17_ESM.zip › Figure 1/1E/WT_scapula_Humerus1.jpg]

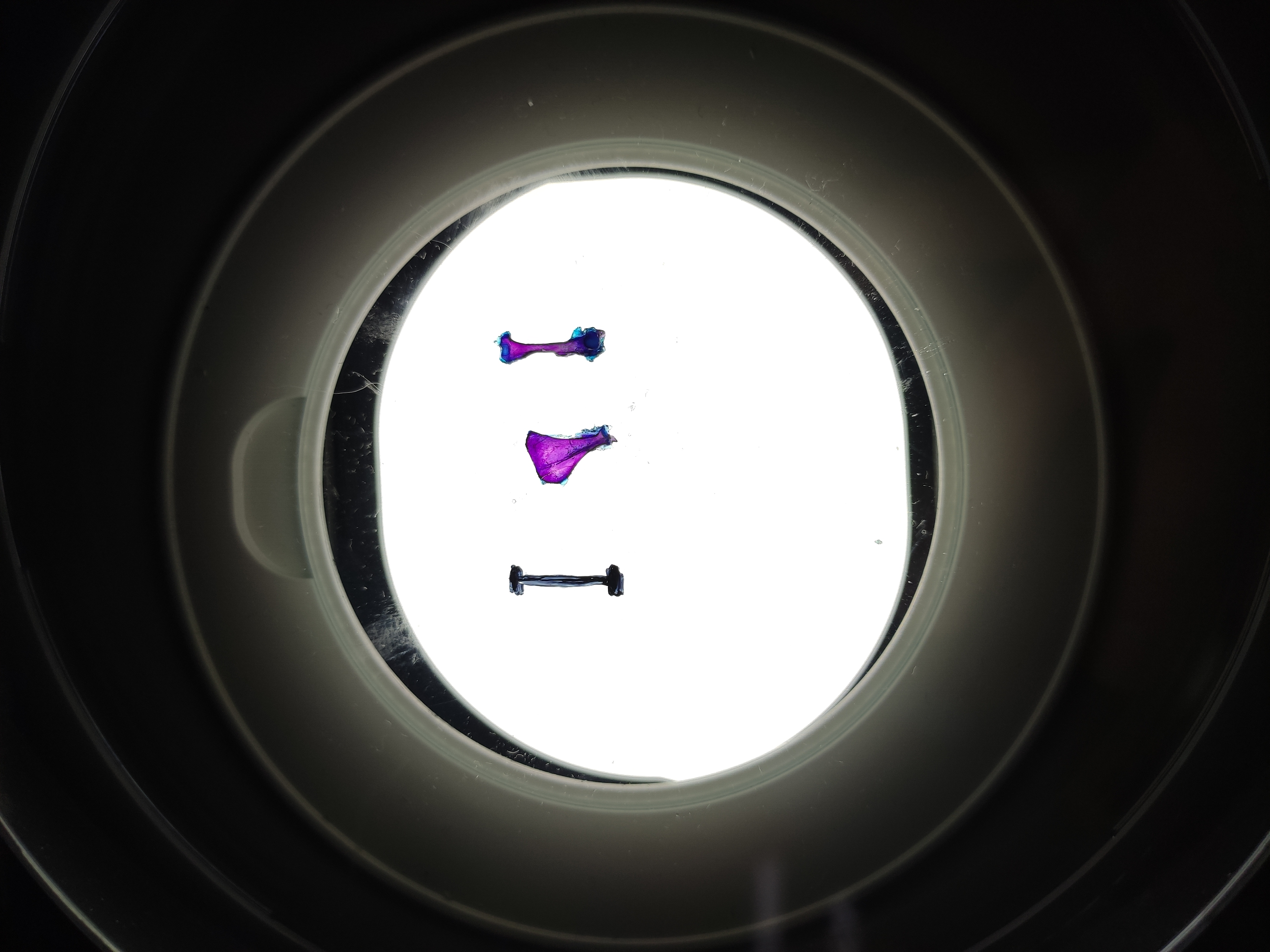

Supplement: Supplementary file 17 — Source data Fig. 1 [file 44319_2024_213_MOESM17_ESM.zip › Figure 1/1E/BCko_Scapula_Humerus1.jpg]

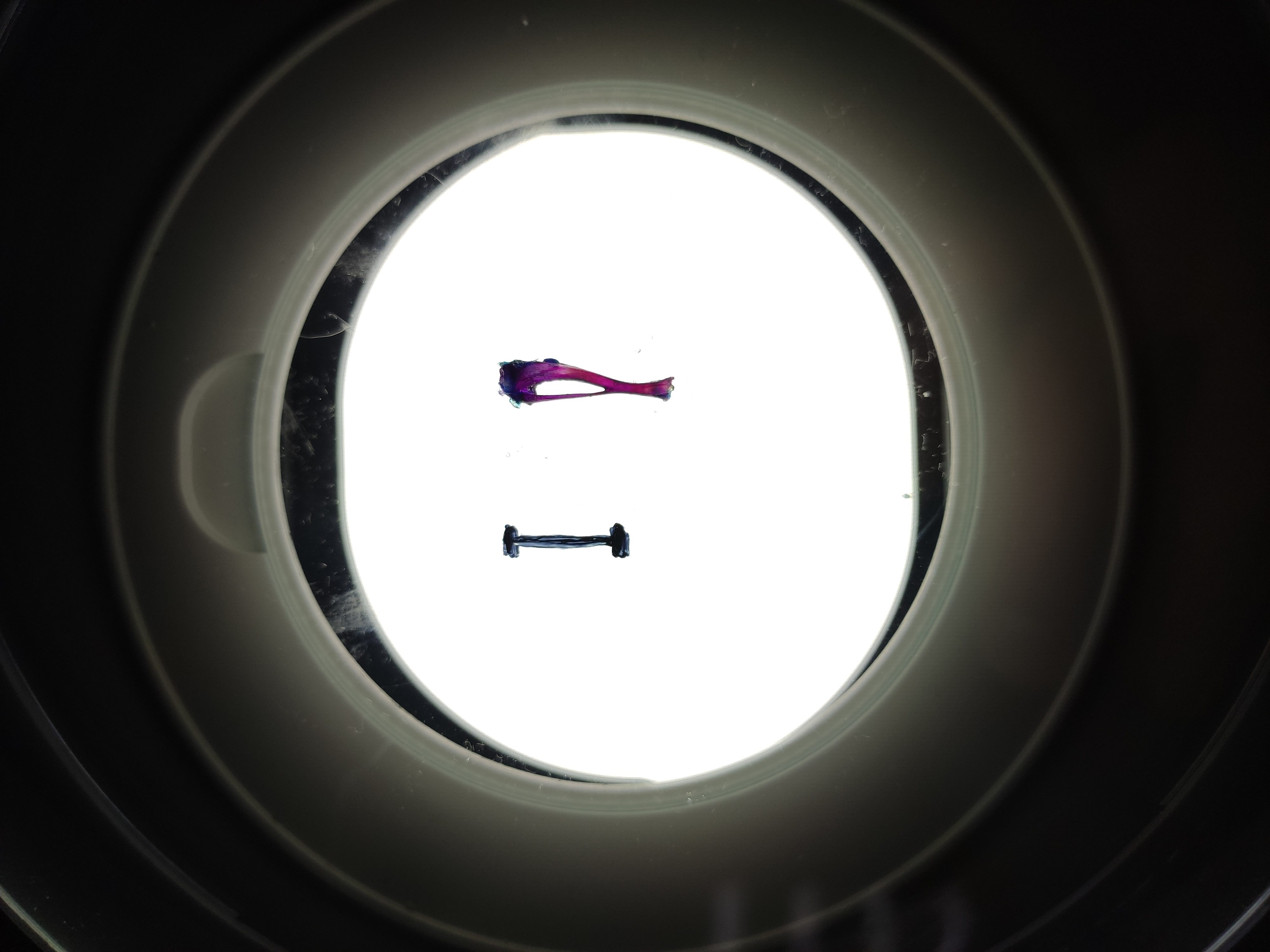

Supplement: Supplementary file 17 — Source data Fig. 1 [file 44319_2024_213_MOESM17_ESM.zip › Figure 1/1E/WT_tibia2.jpg]

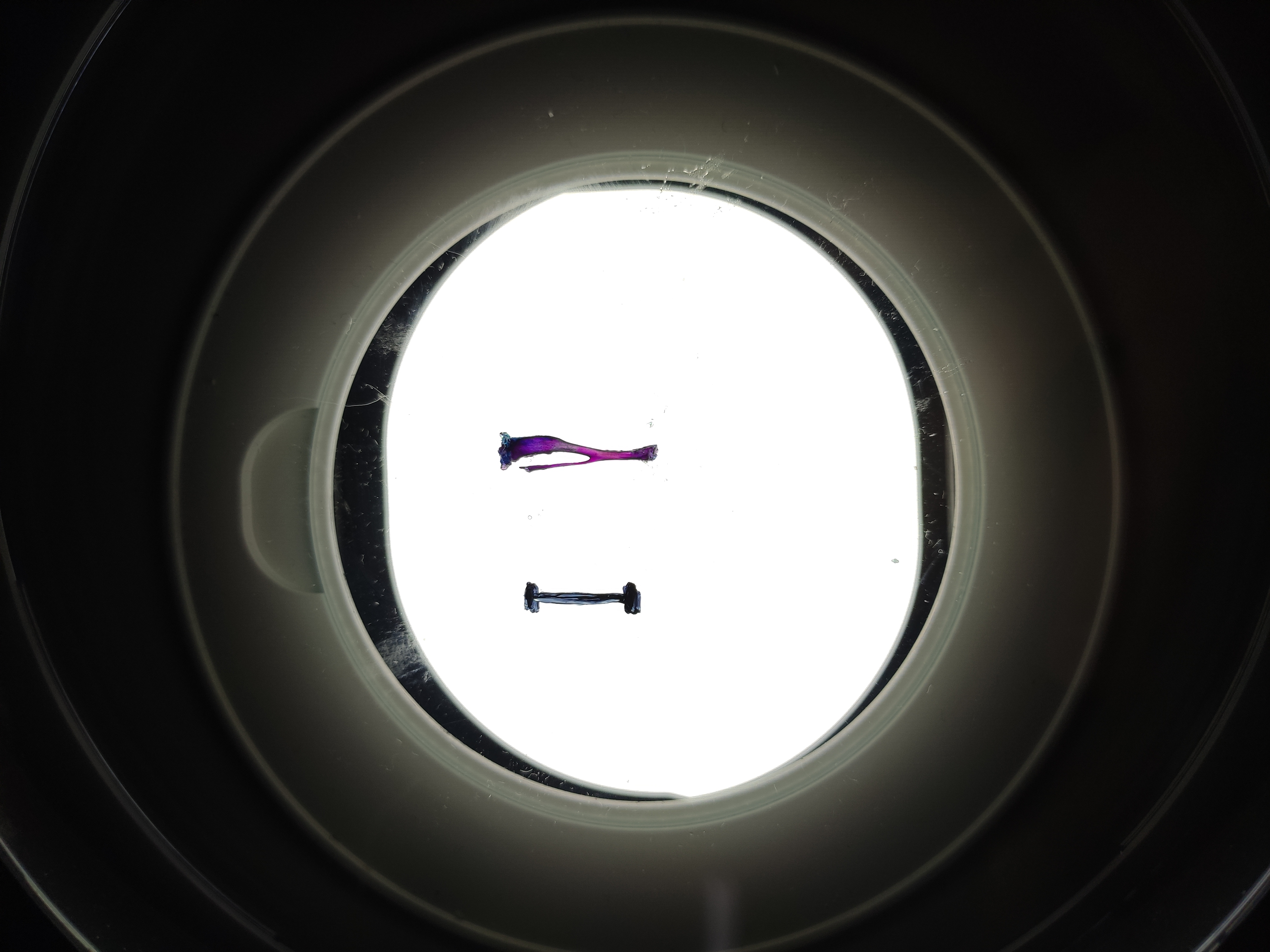

Supplement: Supplementary file 17 — Source data Fig. 1 [file 44319_2024_213_MOESM17_ESM.zip › Figure 1/1E/BCko_tibia1.jpg]

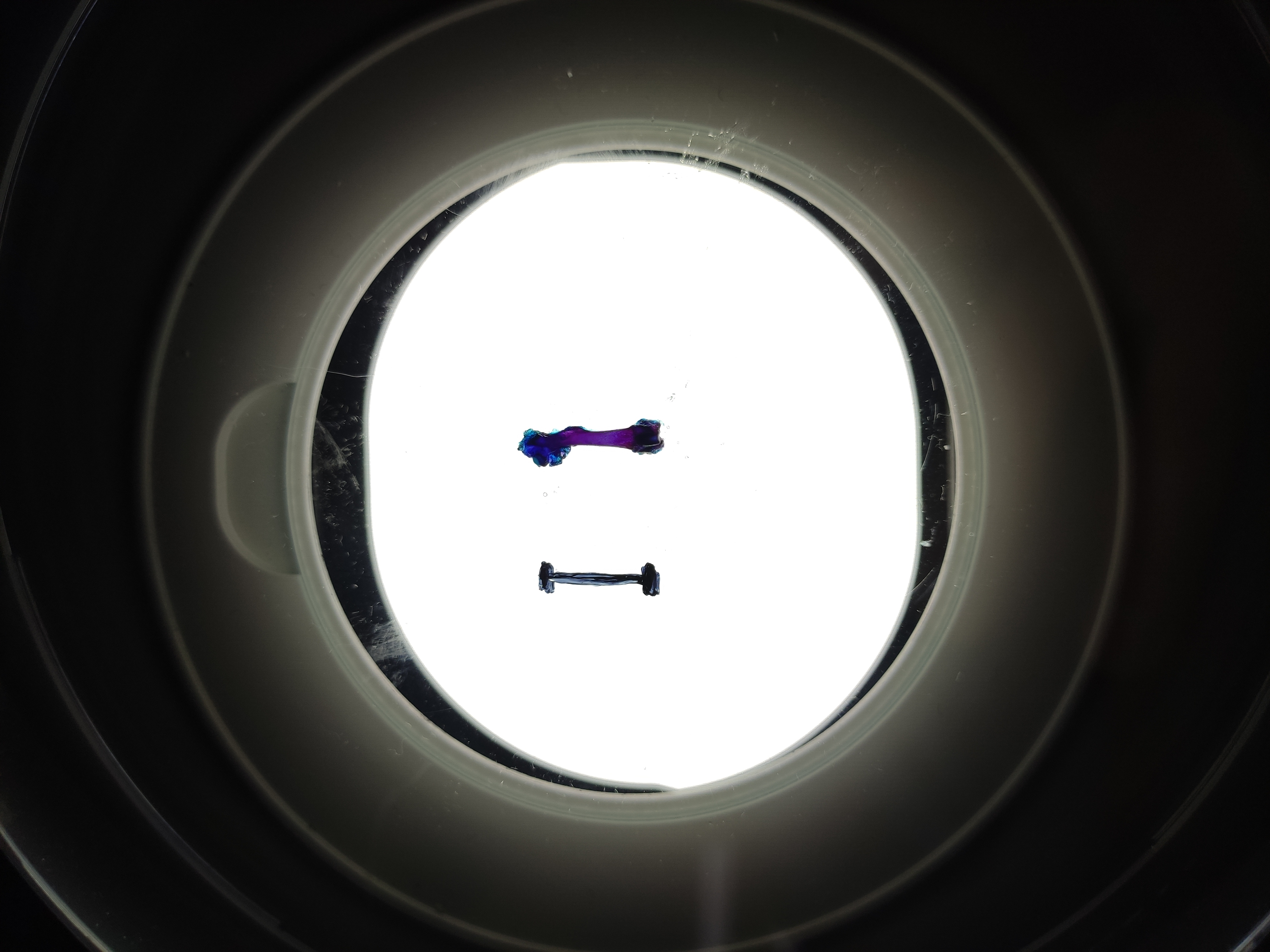

Supplement: Supplementary file 17 — Source data Fig. 1 [file 44319_2024_213_MOESM17_ESM.zip › Figure 1/1E/BCko_femur2.jpg]

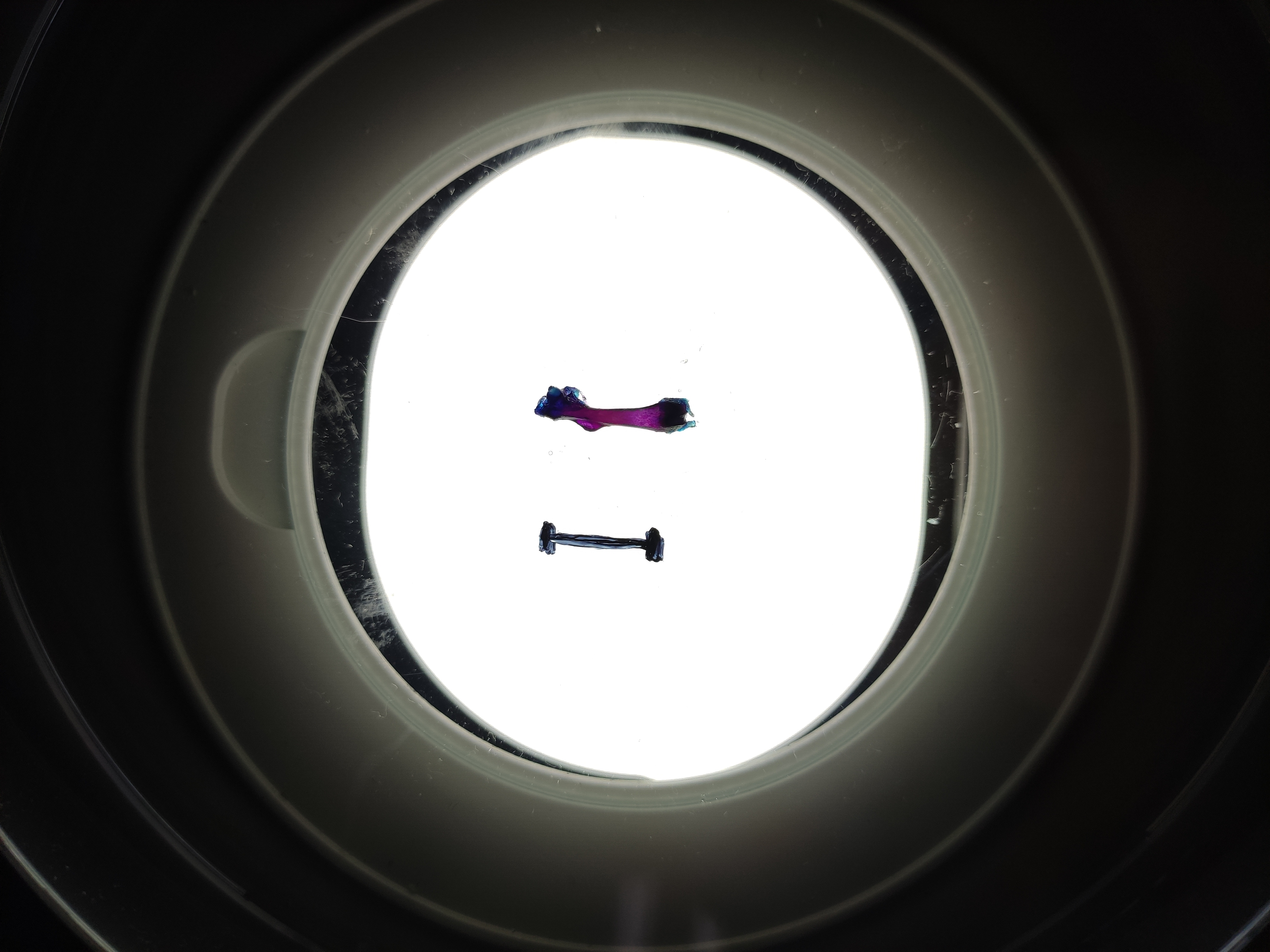

Supplement: Supplementary file 17 — Source data Fig. 1 [file 44319_2024_213_MOESM17_ESM.zip › Figure 1/1E/WT_femur1.jpg]

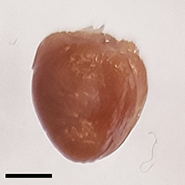

Supplement: Supplementary file 17 — Source data Fig. 1 [file 44319_2024_213_MOESM17_ESM.zip › Figure 1/1D/BC_KO_Heart_1710520663330.tif]

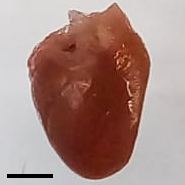

Supplement: Supplementary file 17 — Source data Fig. 1 [file 44319_2024_213_MOESM17_ESM.zip › Figure 1/1D/WT_Heart_1710441286476.tif]

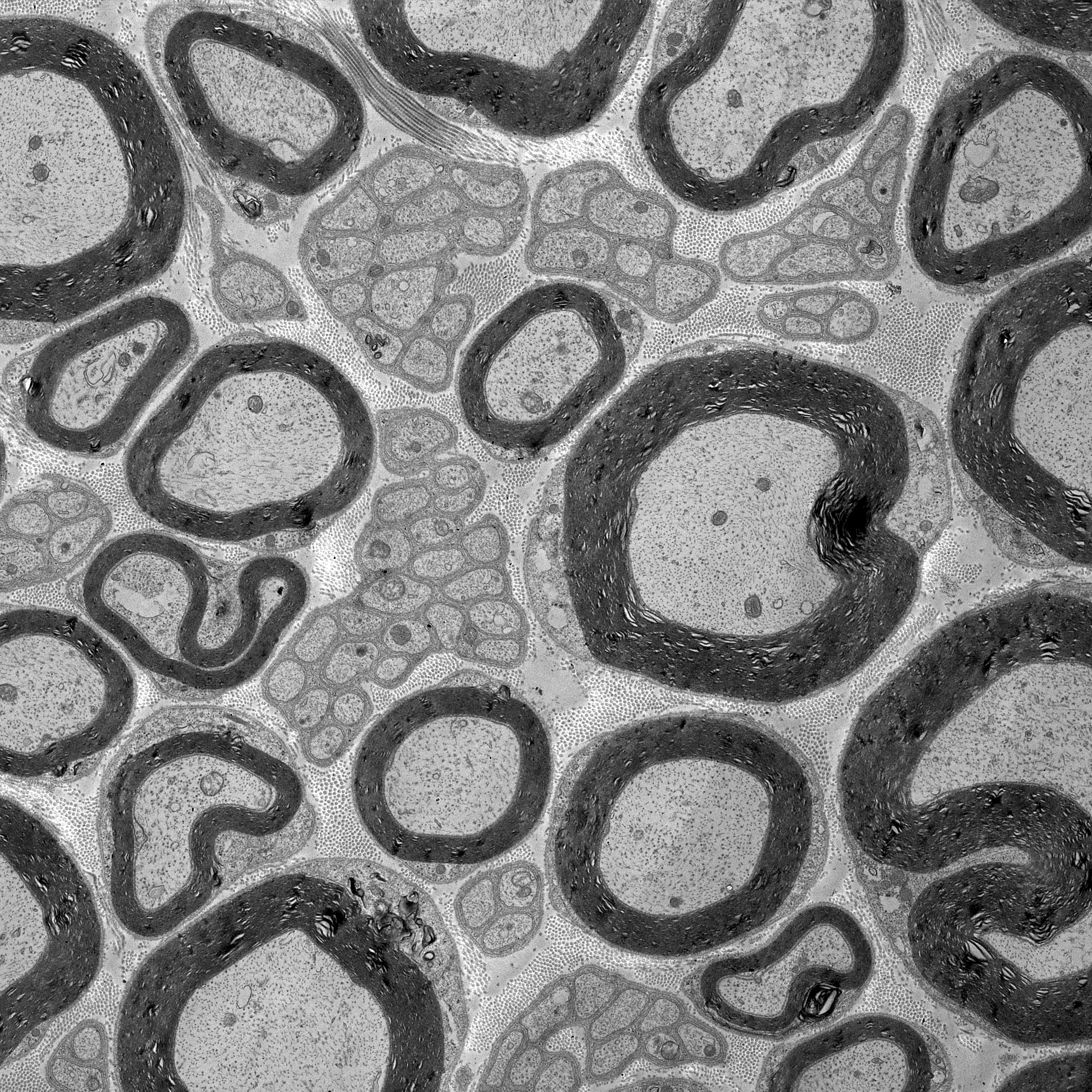

Supplement: Supplementary file 18 — Source data Fig. 2 [file 44319_2024_213_MOESM18_ESM.zip › Figure 2/2I/2500X_2410_WT 15 weeks_0017 nobar.tif]

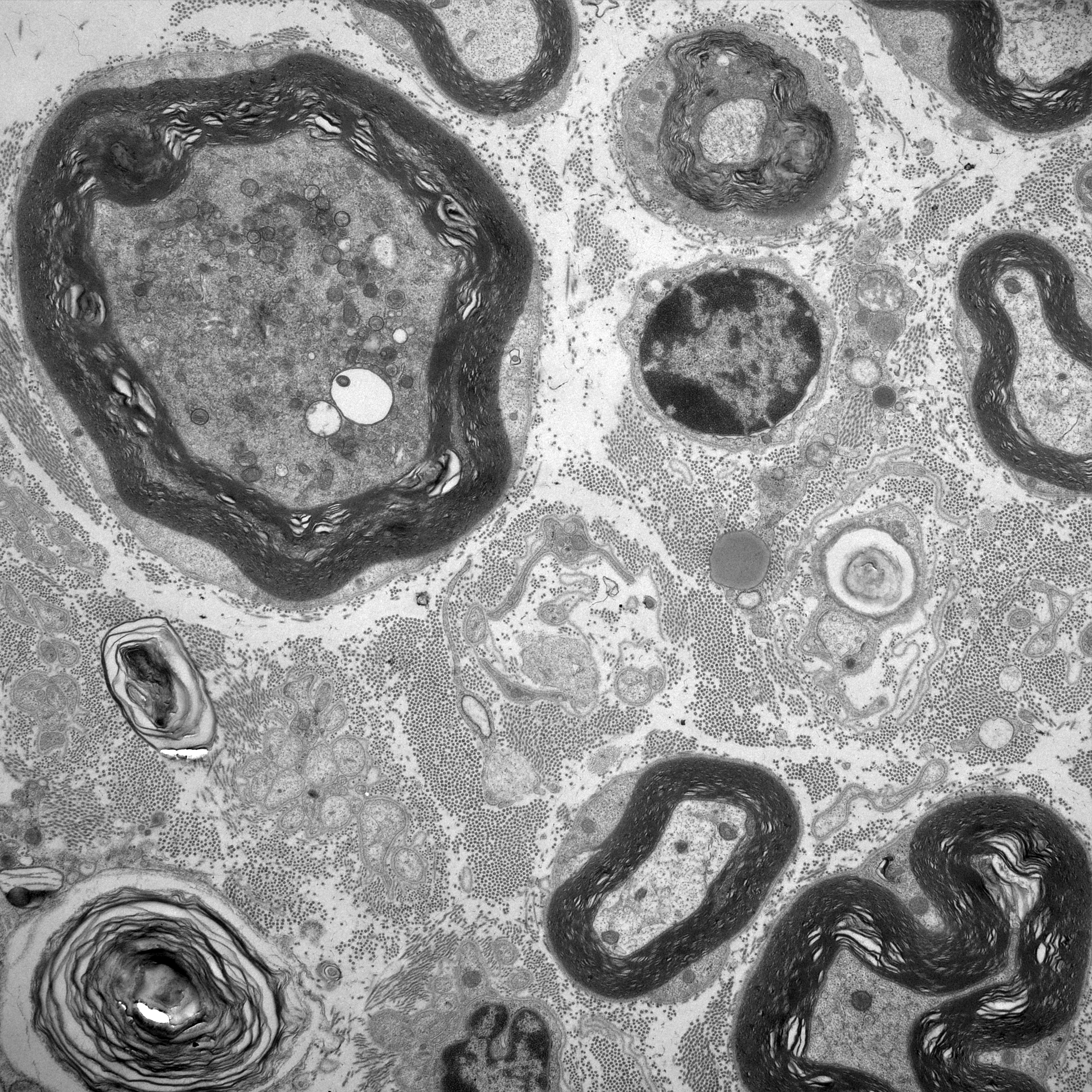

Supplement: Supplementary file 18 — Source data Fig. 2 [file 44319_2024_213_MOESM18_ESM.zip › Figure 2/2I/2500X_2425__BCKO 15 weeks_0018 no bar.tiff]

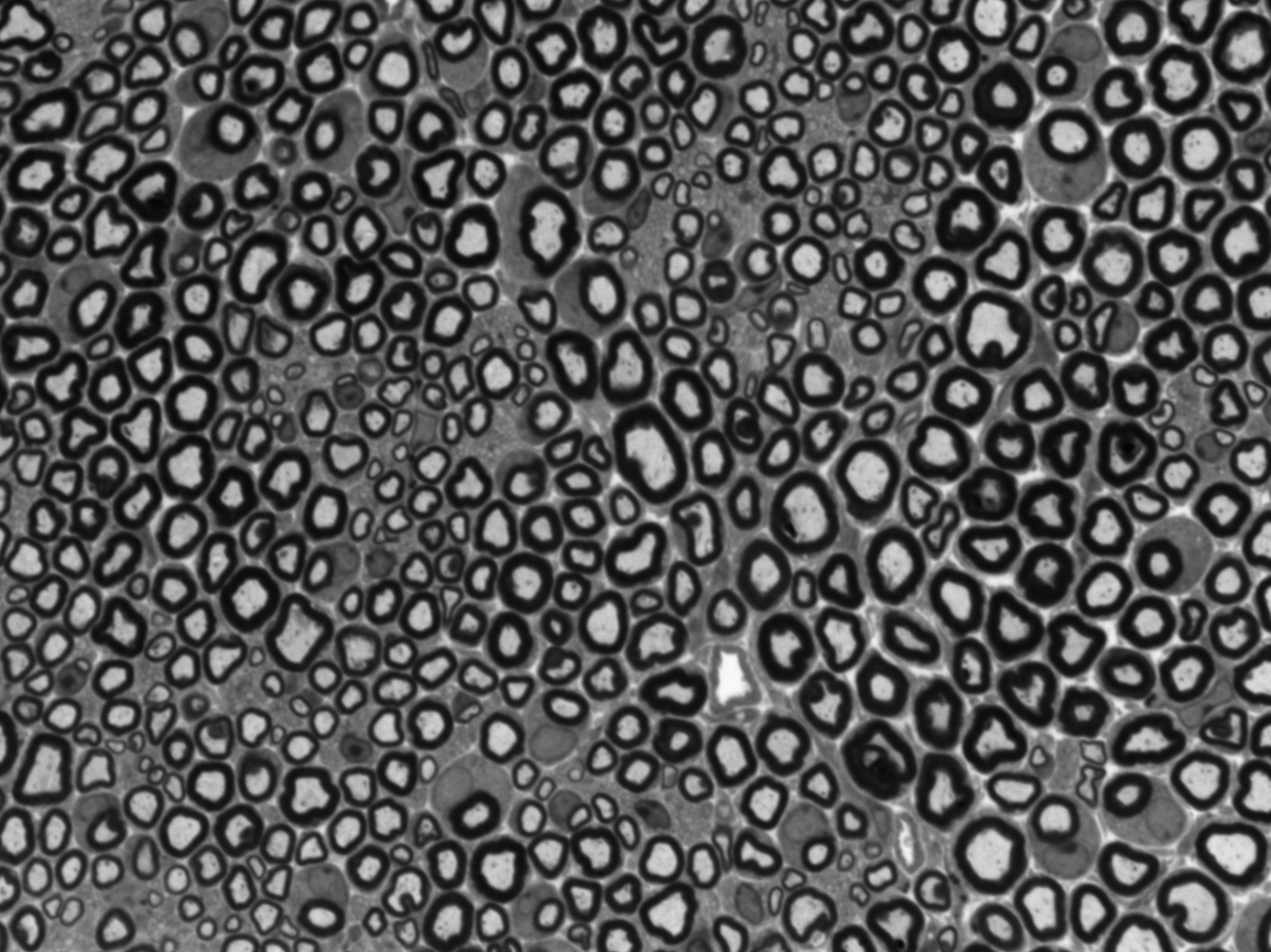

Supplement: Supplementary file 18 — Source data Fig. 2 [file 44319_2024_213_MOESM18_ESM.zip › Figure 2/2A/BKO 4 weeks Snap-1597.czi - C=0.tif]

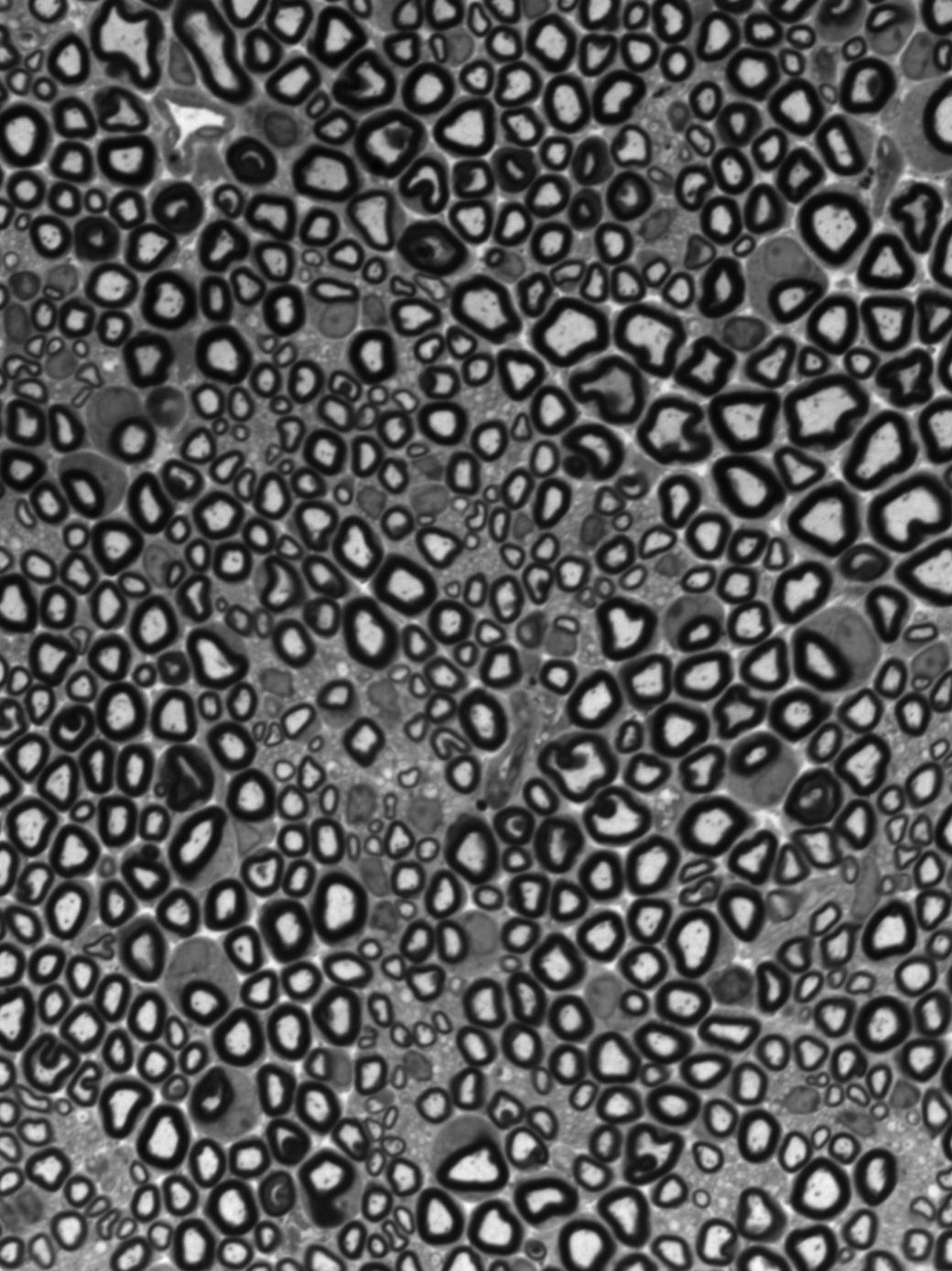

Supplement: Supplementary file 18 — Source data Fig. 2 [file 44319_2024_213_MOESM18_ESM.zip › Figure 2/2A/CKO 4 weeks Snap-1592.czi - C=0.tif]

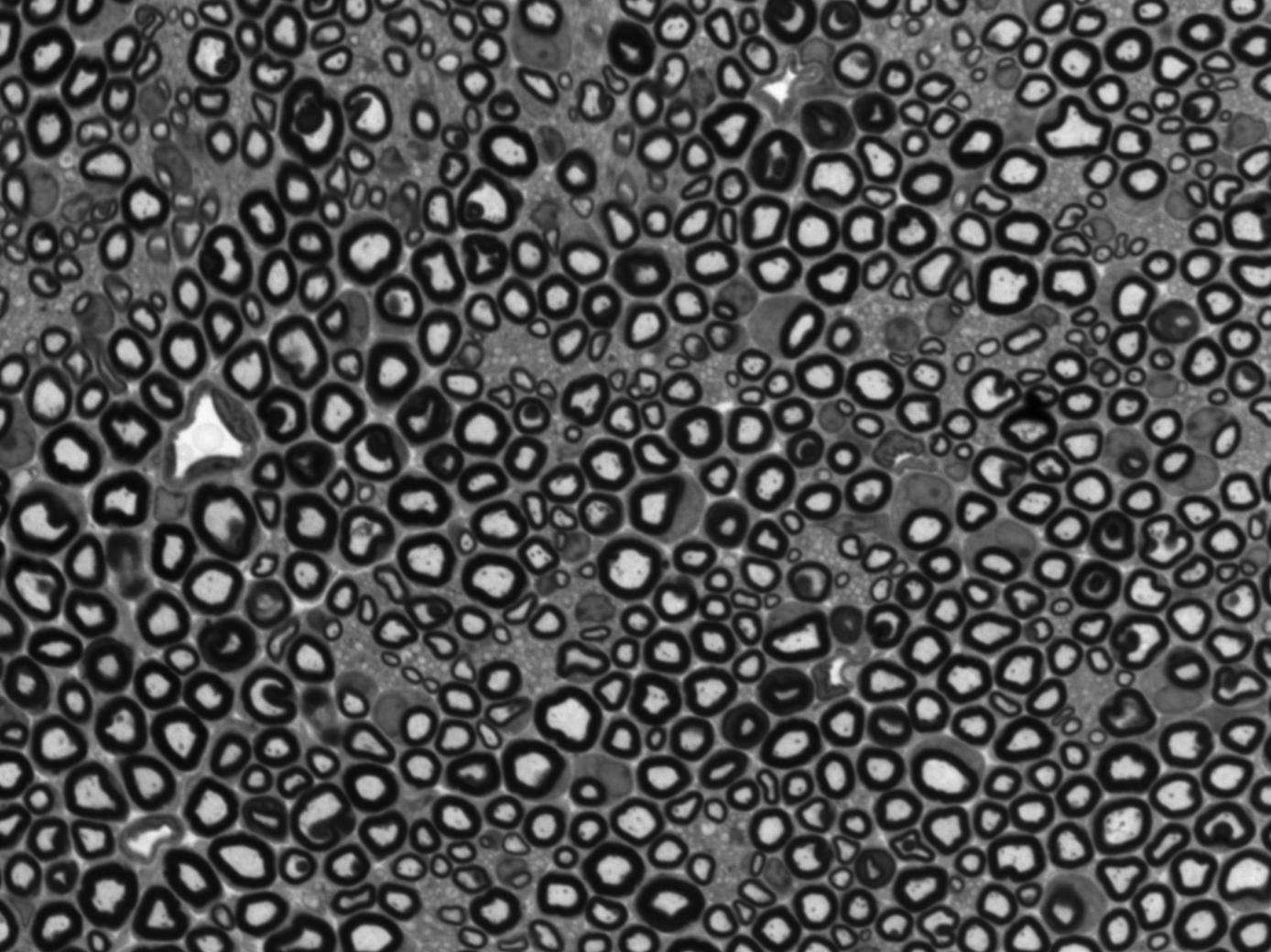

Supplement: Supplementary file 18 — Source data Fig. 2 [file 44319_2024_213_MOESM18_ESM.zip › Figure 2/2A/WT 4 weeks Snap-1600.czi - C=0.tif]

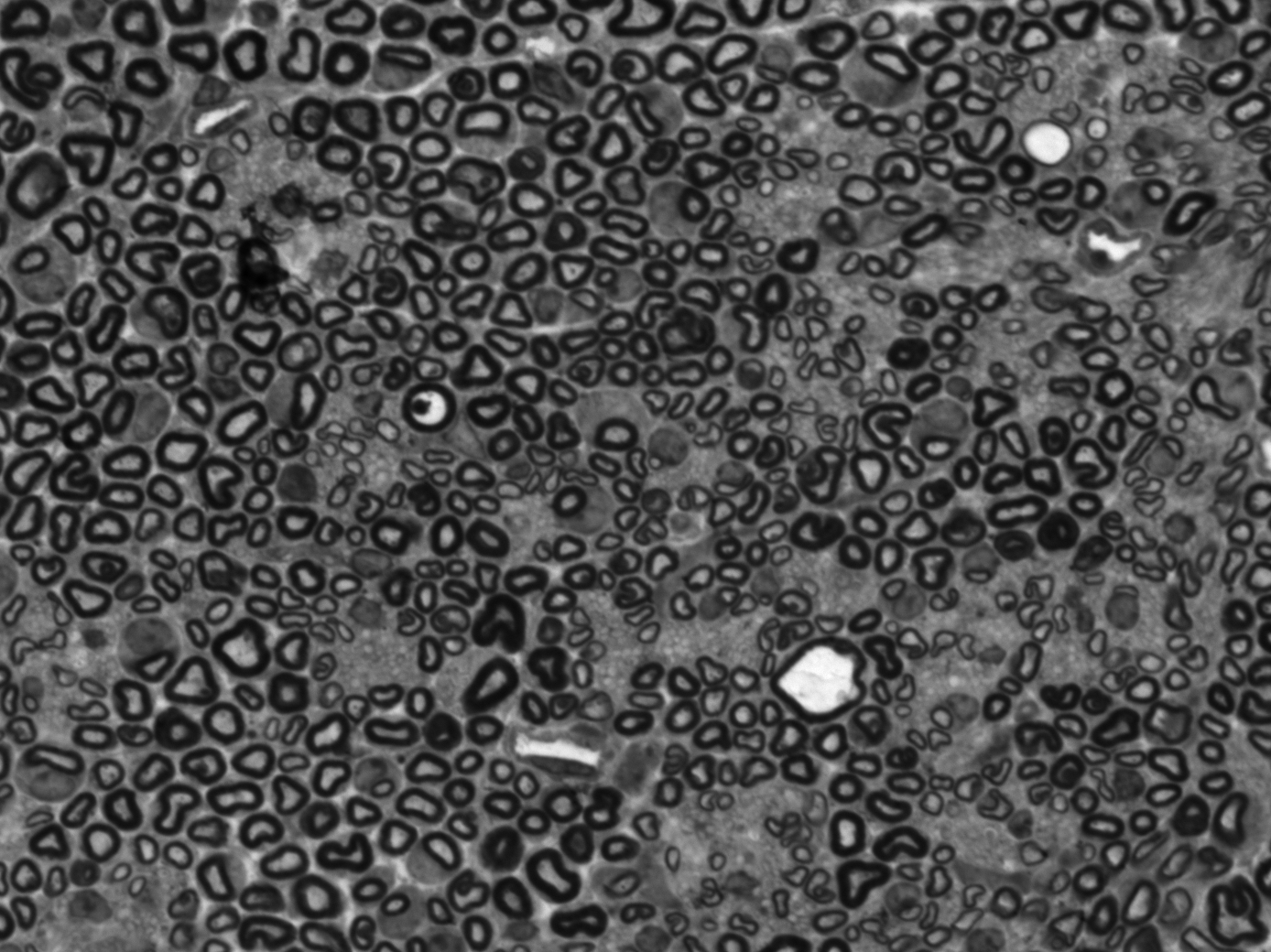

Supplement: Supplementary file 18 — Source data Fig. 2 [file 44319_2024_213_MOESM18_ESM.zip › Figure 2/2A/BCKO 4 weeks Snap-1586.czi - C=0.tif]

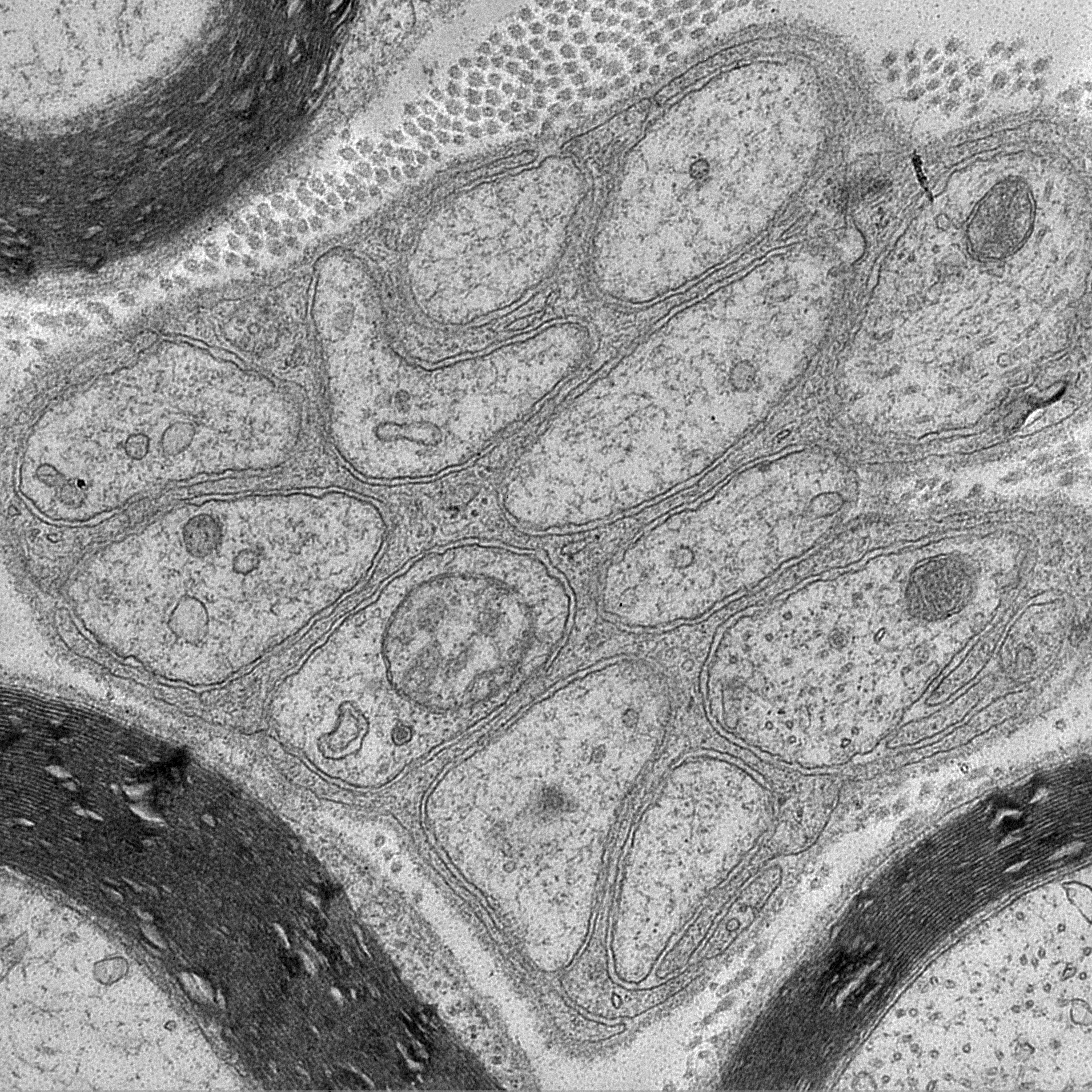

Supplement: Supplementary file 18 — Source data Fig. 2 [file 44319_2024_213_MOESM18_ESM.zip › Figure 2/2J/WT 12kX_2410__0006 no bar.tiff]

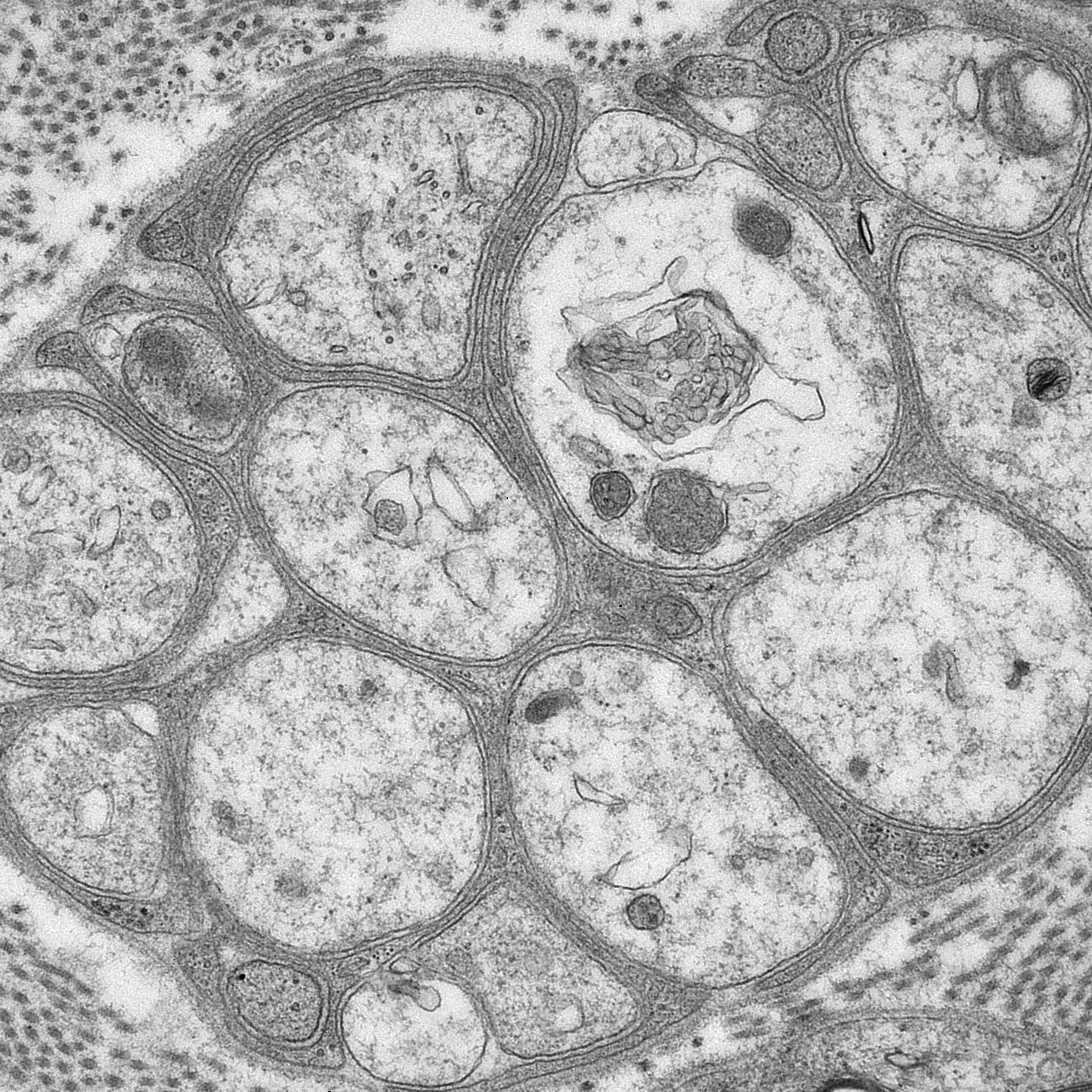

Supplement: Supplementary file 18 — Source data Fig. 2 [file 44319_2024_213_MOESM18_ESM.zip › Figure 2/2J/BC KO 12kX_2426__0001 no bar.tiff]

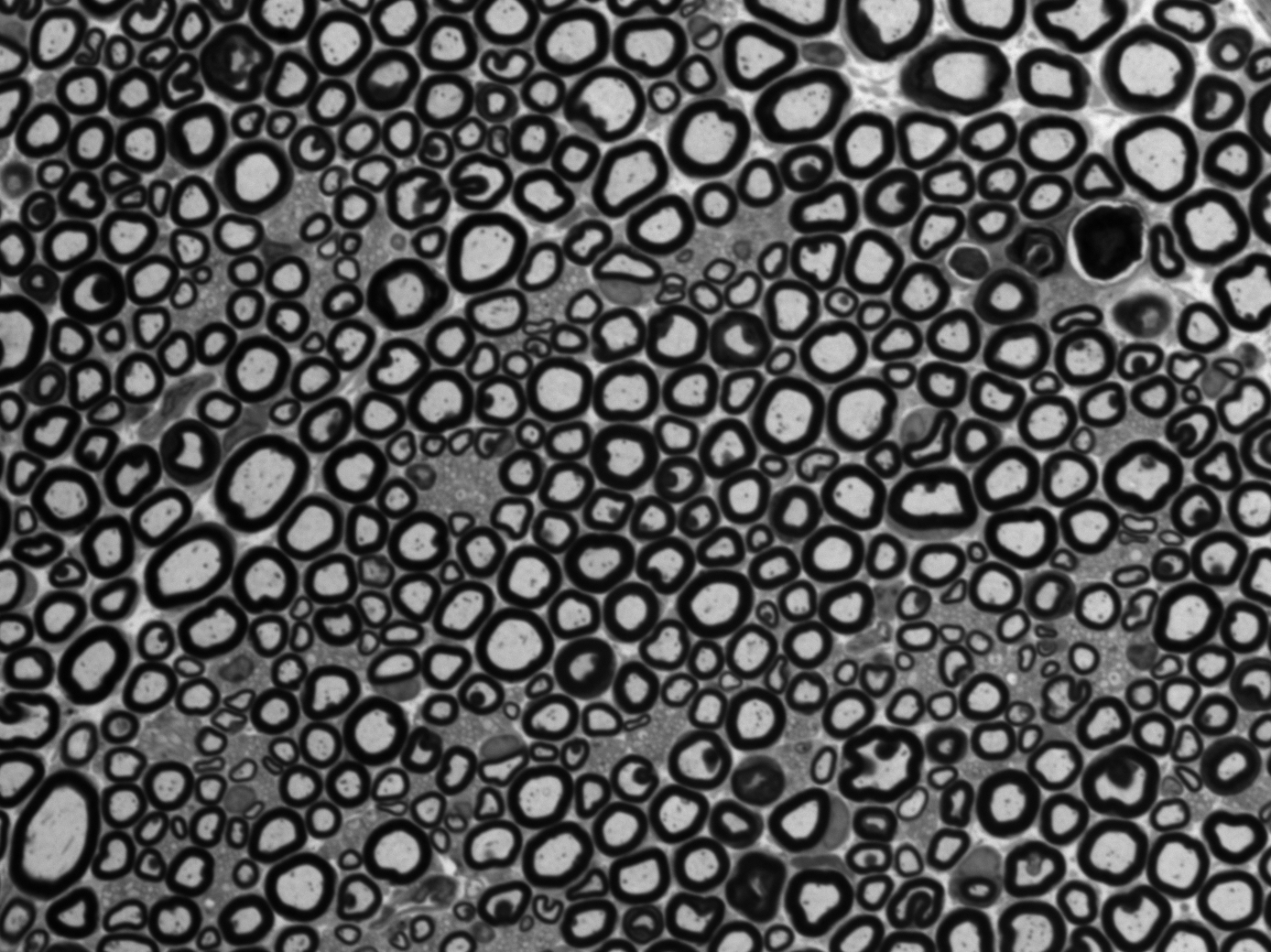

Supplement: Supplementary file 18 — Source data Fig. 2 [file 44319_2024_213_MOESM18_ESM.zip › Figure 2/2E/CKO 15 weeks Snap-1633.czi - C=0.tif]

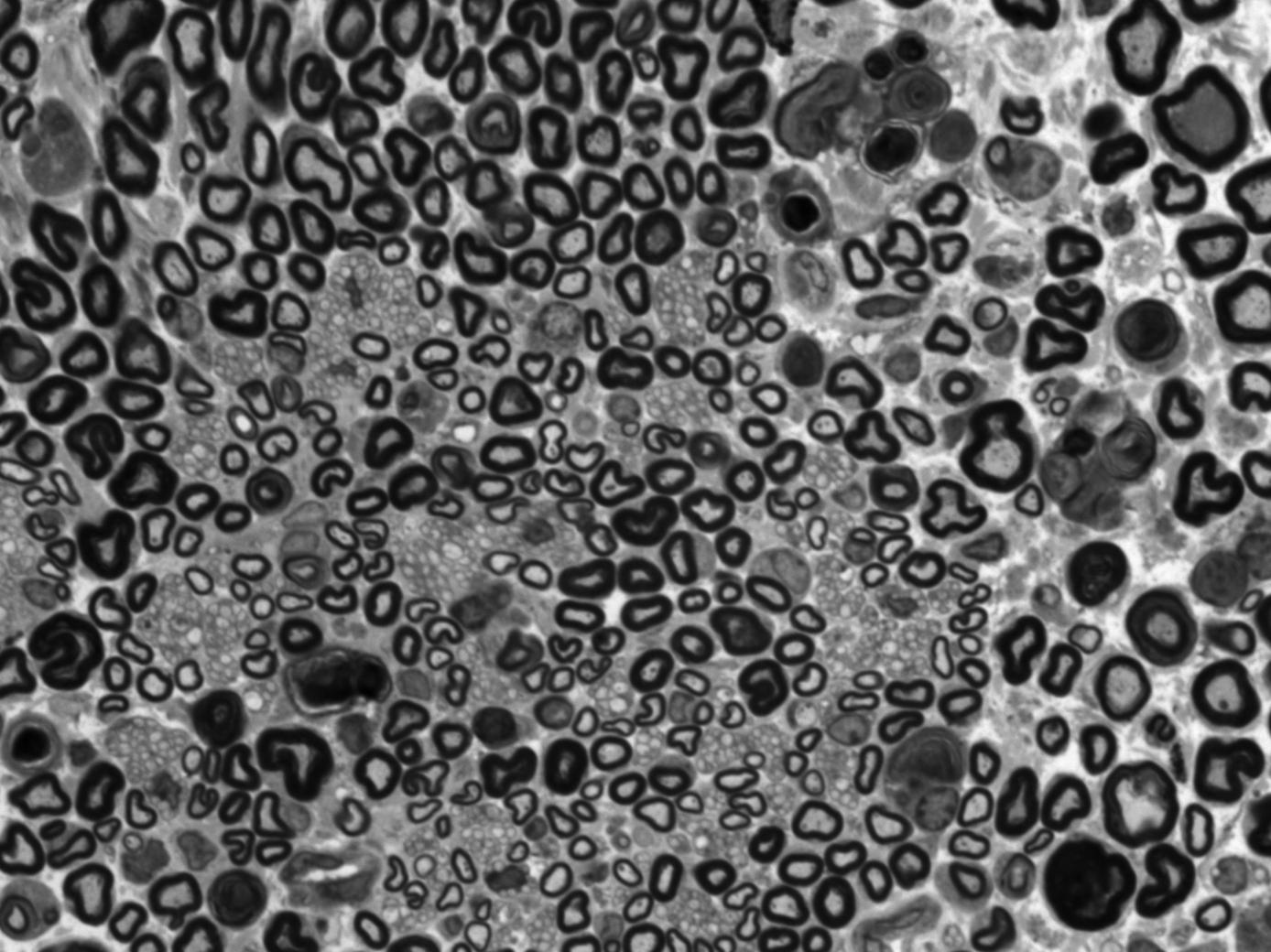

Supplement: Supplementary file 18 — Source data Fig. 2 [file 44319_2024_213_MOESM18_ESM.zip › Figure 2/2E/BCKO 15 weeks Snap-1611.czi - C=0.tif]

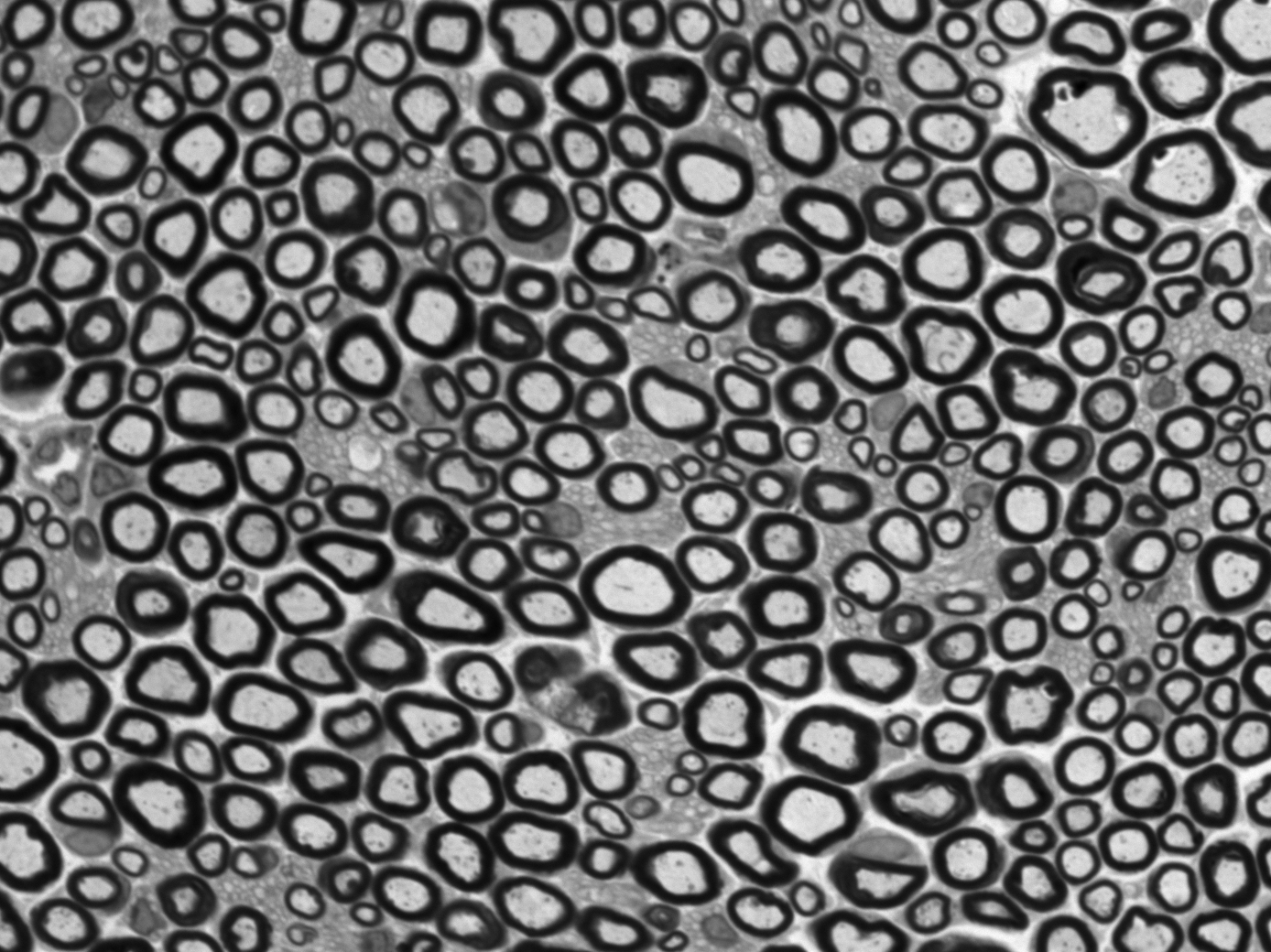

Supplement: Supplementary file 18 — Source data Fig. 2 [file 44319_2024_213_MOESM18_ESM.zip › Figure 2/2E/BKO 15 weeks Snap-1620.czi - C=0.tif]

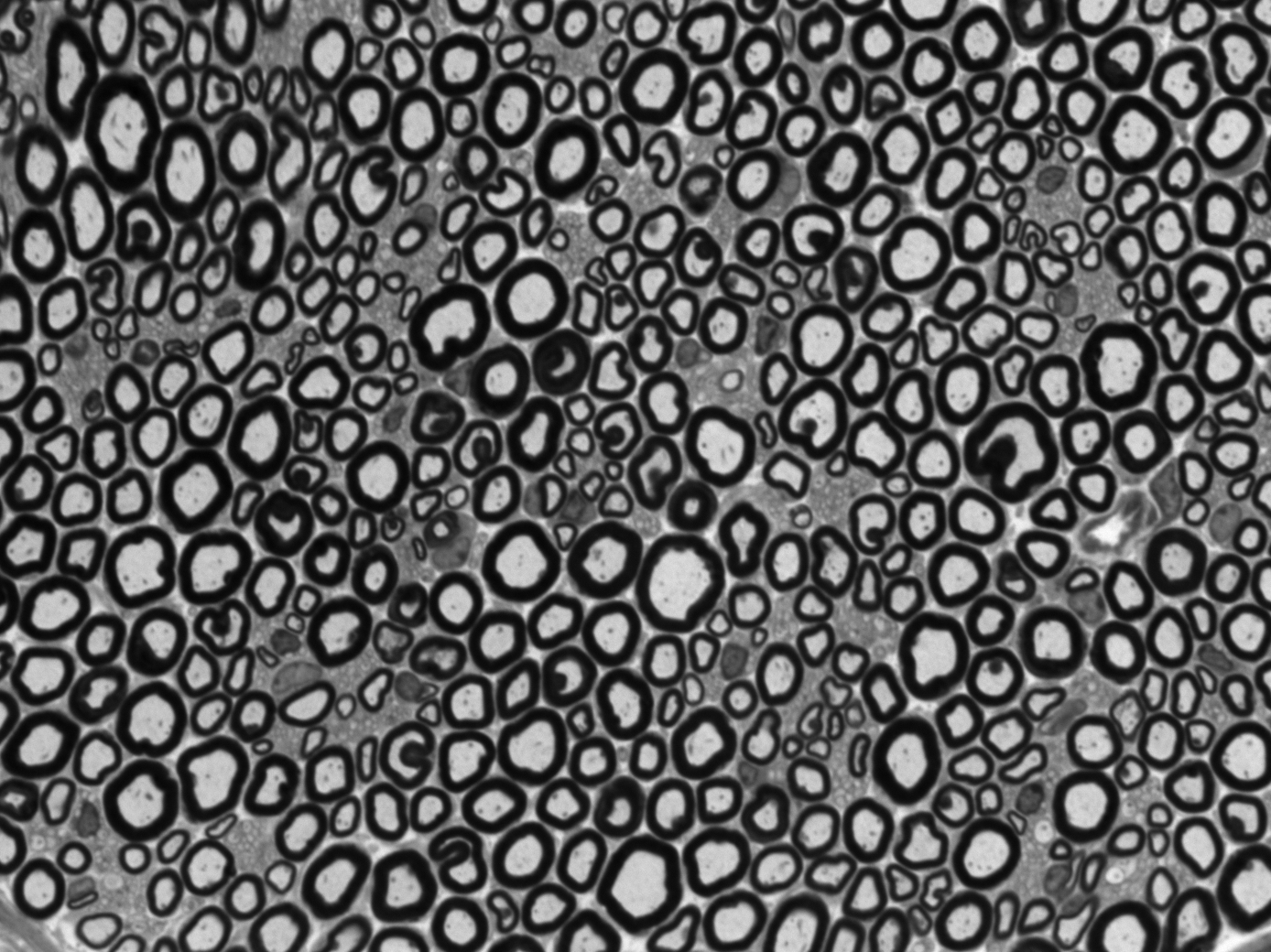

Supplement: Supplementary file 18 — Source data Fig. 2 [file 44319_2024_213_MOESM18_ESM.zip › Figure 2/2E/WT 15 weeks Snap-1629.czi - C=0.tif]

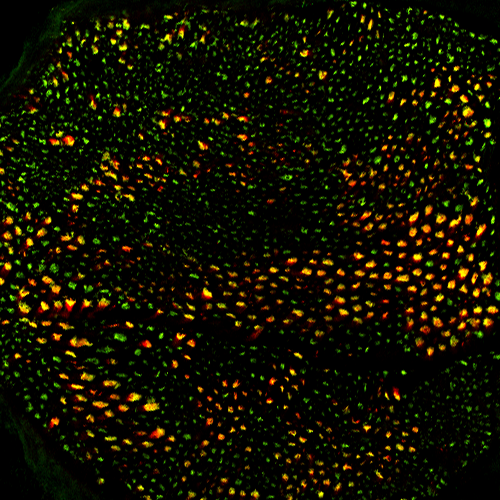

Supplement: Supplementary file 19 — Source data Fig. 3 [file 44319_2024_213_MOESM19_ESM.zip › Figure 3/3B/BKO.tif]

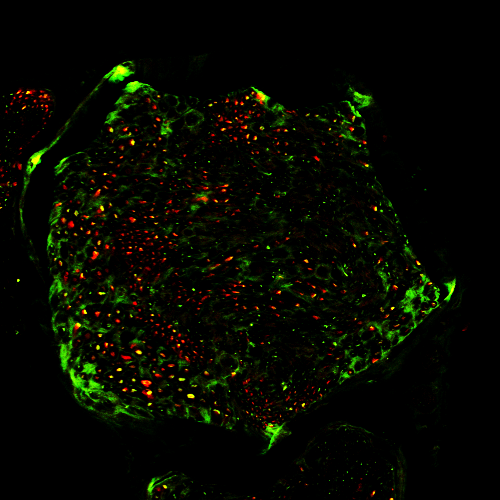

Supplement: Supplementary file 19 — Source data Fig. 3 [file 44319_2024_213_MOESM19_ESM.zip › Figure 3/3B/BCKO.tif]

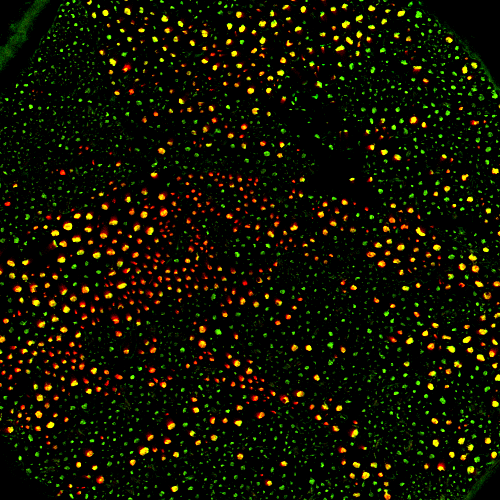

Supplement: Supplementary file 19 — Source data Fig. 3 [file 44319_2024_213_MOESM19_ESM.zip › Figure 3/3B/CKO.tif]

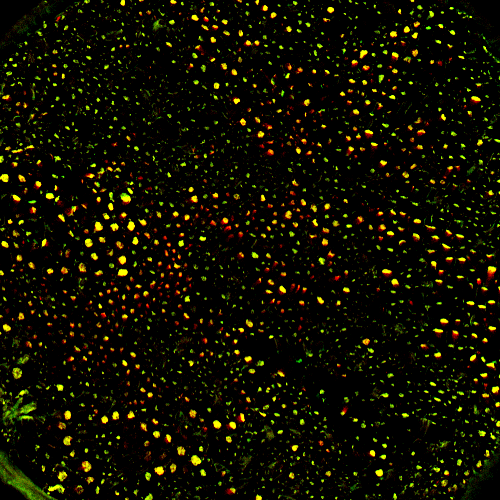

Supplement: Supplementary file 19 — Source data Fig. 3 [file 44319_2024_213_MOESM19_ESM.zip › Figure 3/3B/WT.tif]

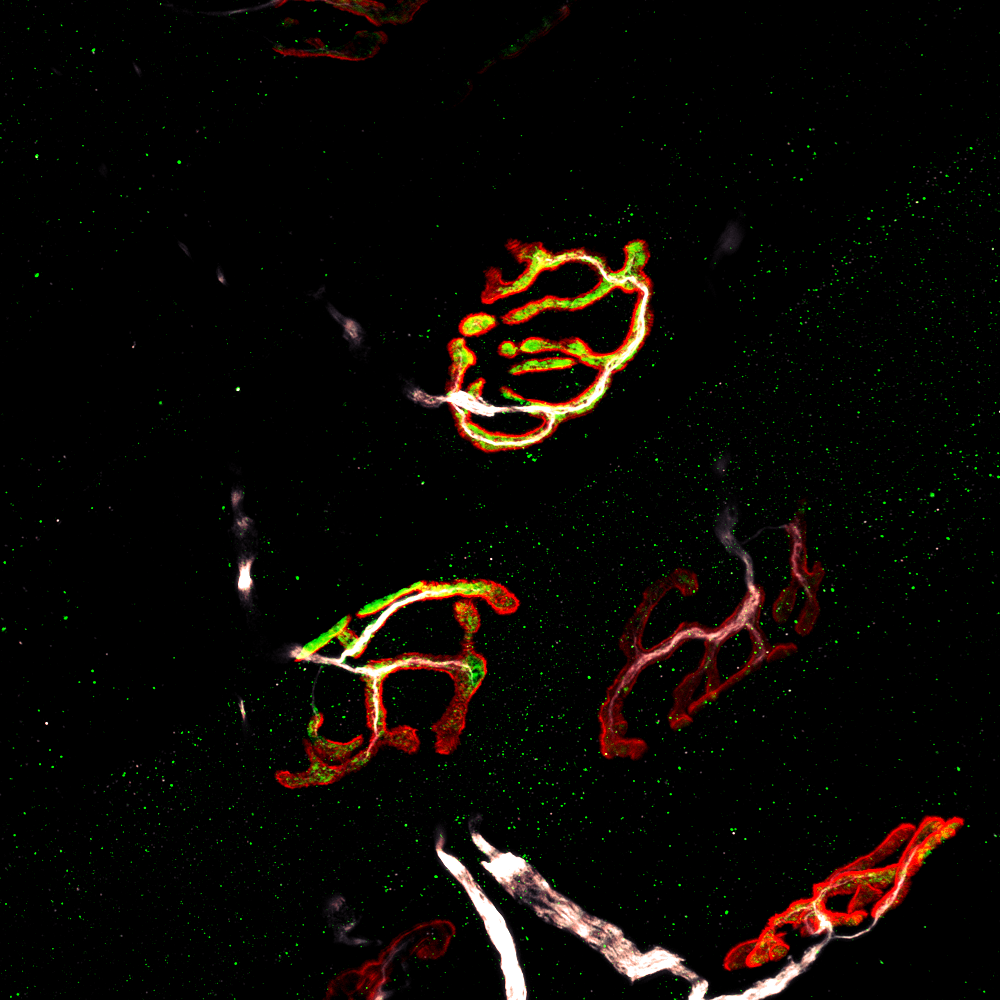

Supplement: Supplementary file 19 — Source data Fig. 3 [file 44319_2024_213_MOESM19_ESM.zip › Figure 3/3G/BKO_3506_Image 3_Airyscan Processing_Maximum intensity projection.lsm (RGB).tif]

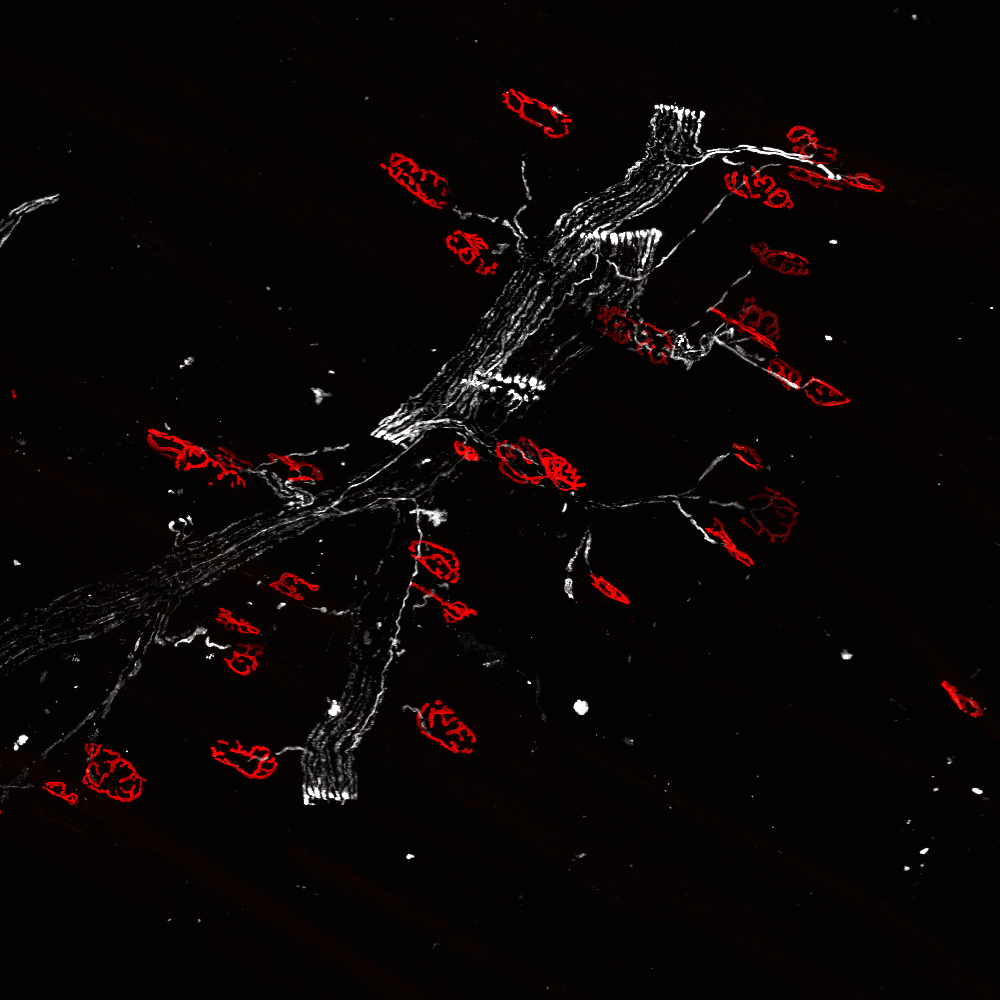

Supplement: Supplementary file 19 — Source data Fig. 3 [file 44319_2024_213_MOESM19_ESM.zip › Figure 3/3G/WT_3485_(merge)Image 14_Airyscan Processing_Maximum intensity projection.lsm (RGB).tif]

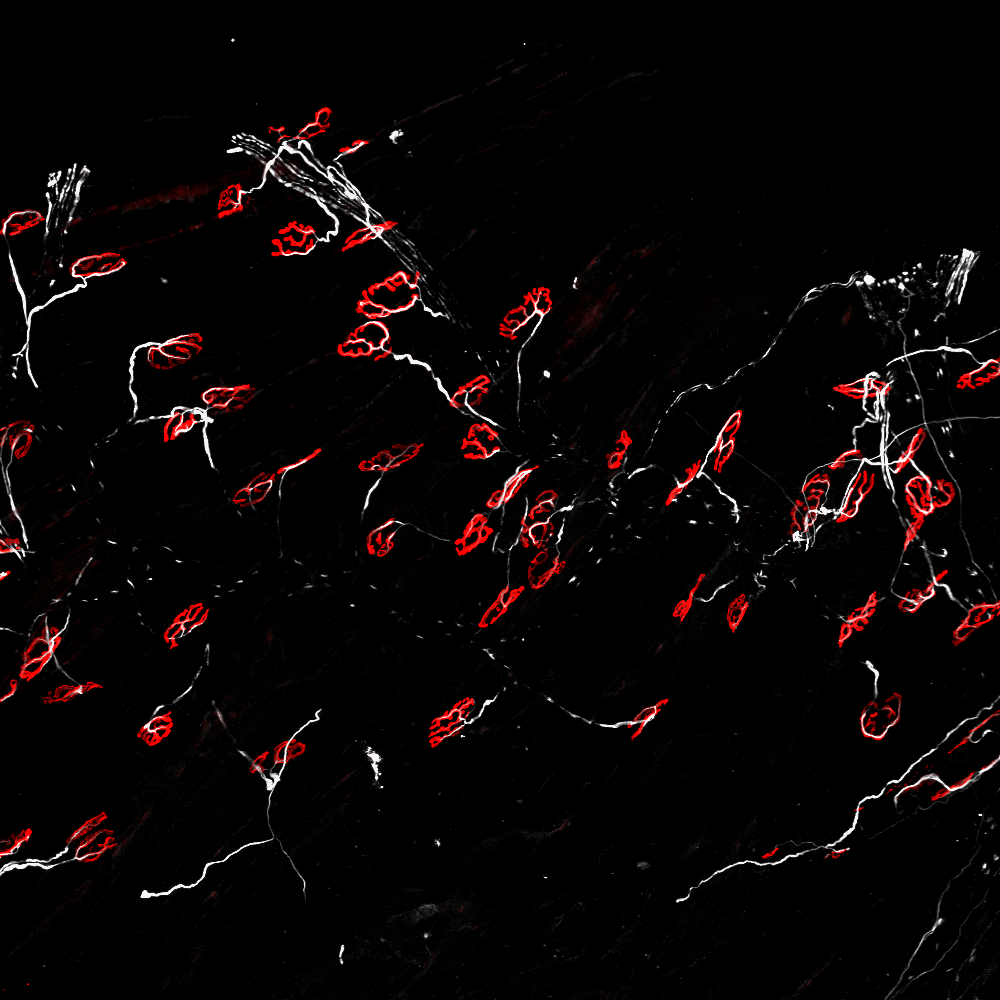

Supplement: Supplementary file 19 — Source data Fig. 3 [file 44319_2024_213_MOESM19_ESM.zip › Figure 3/3G/BKO_3478_(merge)Image 12_Airyscan Processing_Maximum intensity projection.lsm (RGB).tif]

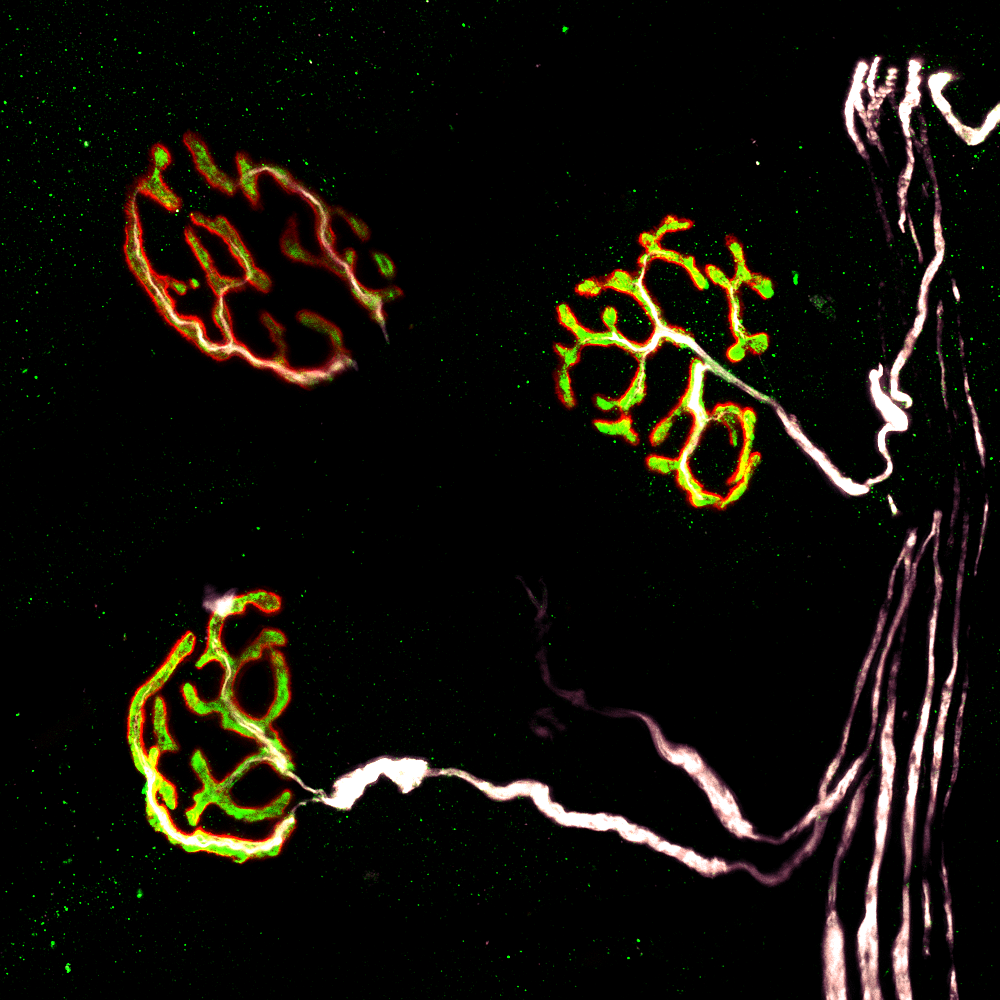

Supplement: Supplementary file 19 — Source data Fig. 3 [file 44319_2024_213_MOESM19_ESM.zip › Figure 3/3G/WT_Image 18(merge)_Airyscan Processing_Maximum intensity projection.lsm (RGB).tif]

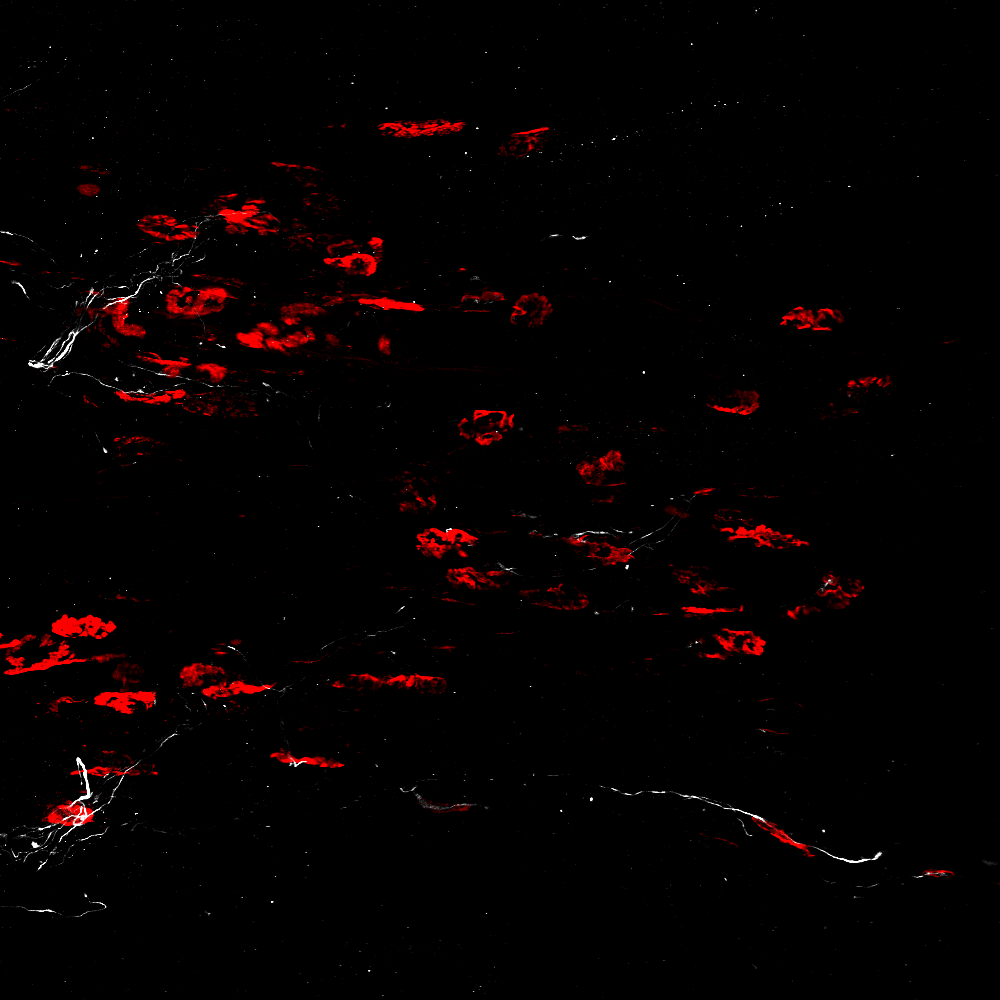

Supplement: Supplementary file 19 — Source data Fig. 3 [file 44319_2024_213_MOESM19_ESM.zip › Figure 3/3G/BCKO_3446_(merge)Image 1_Airyscan Processing_Maximum intensity projection.lsm (RGB).tif]

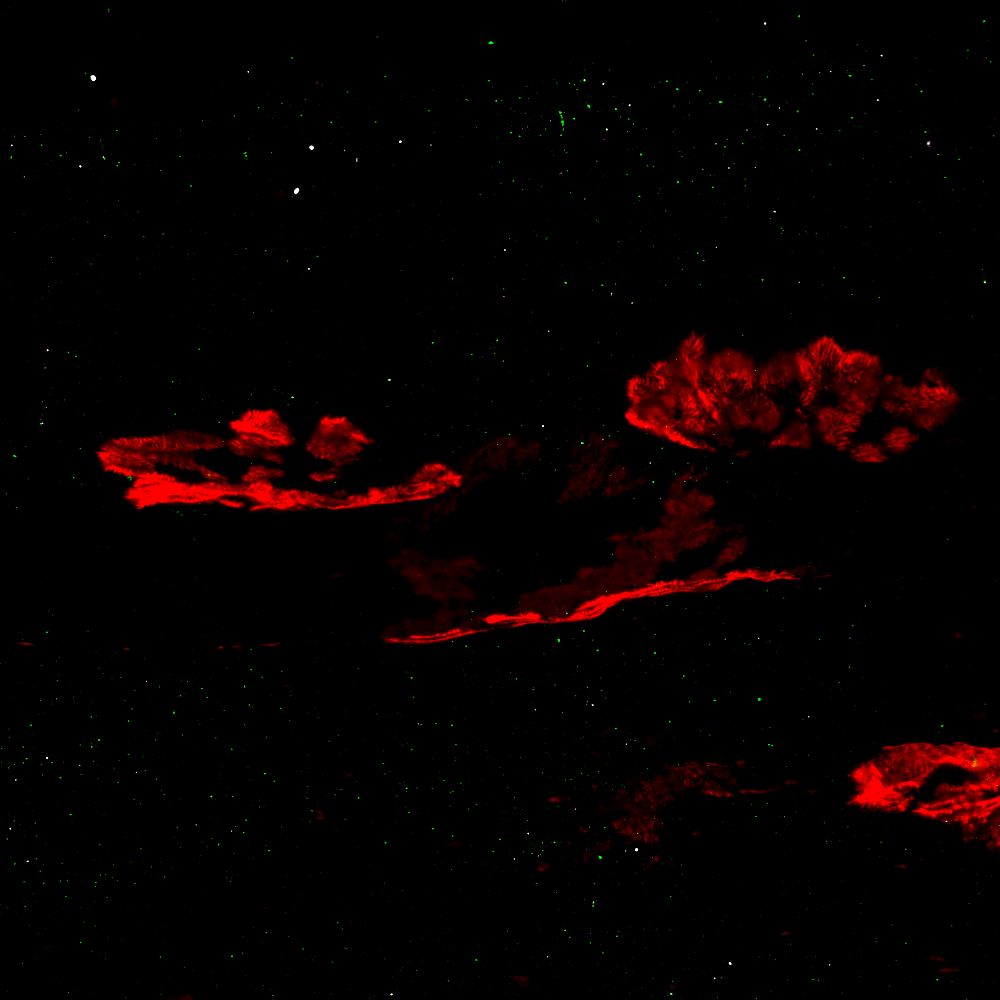

Supplement: Supplementary file 19 — Source data Fig. 3 [file 44319_2024_213_MOESM19_ESM.zip › Figure 3/3G/BCKO_3446_Image 6_Airyscan Processing_Maximum intensity projection.lsm (RGB).tif]

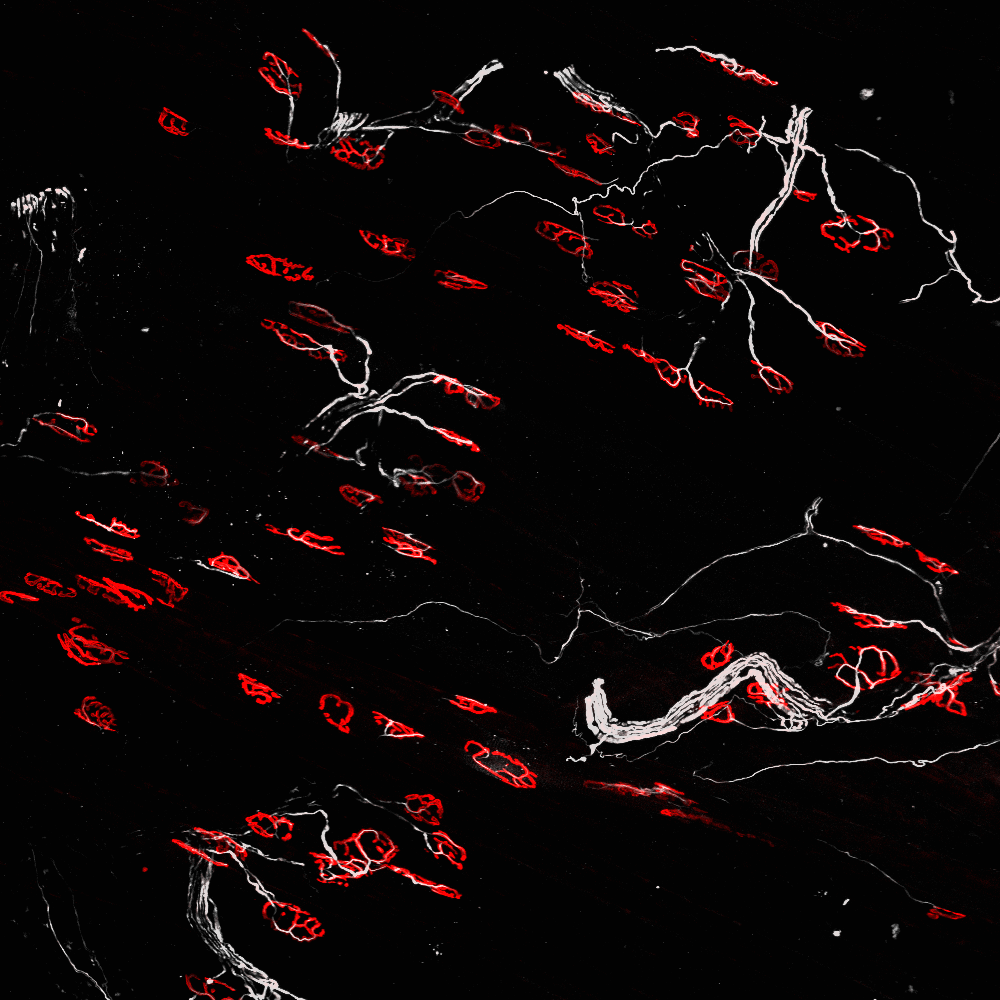

Supplement: Supplementary file 19 — Source data Fig. 3 [file 44319_2024_213_MOESM19_ESM.zip › Figure 3/3G/CKO_3463_(merge)Image 17_Airyscan Processing_Maximum intensity projection.lsm (RGB).tif]

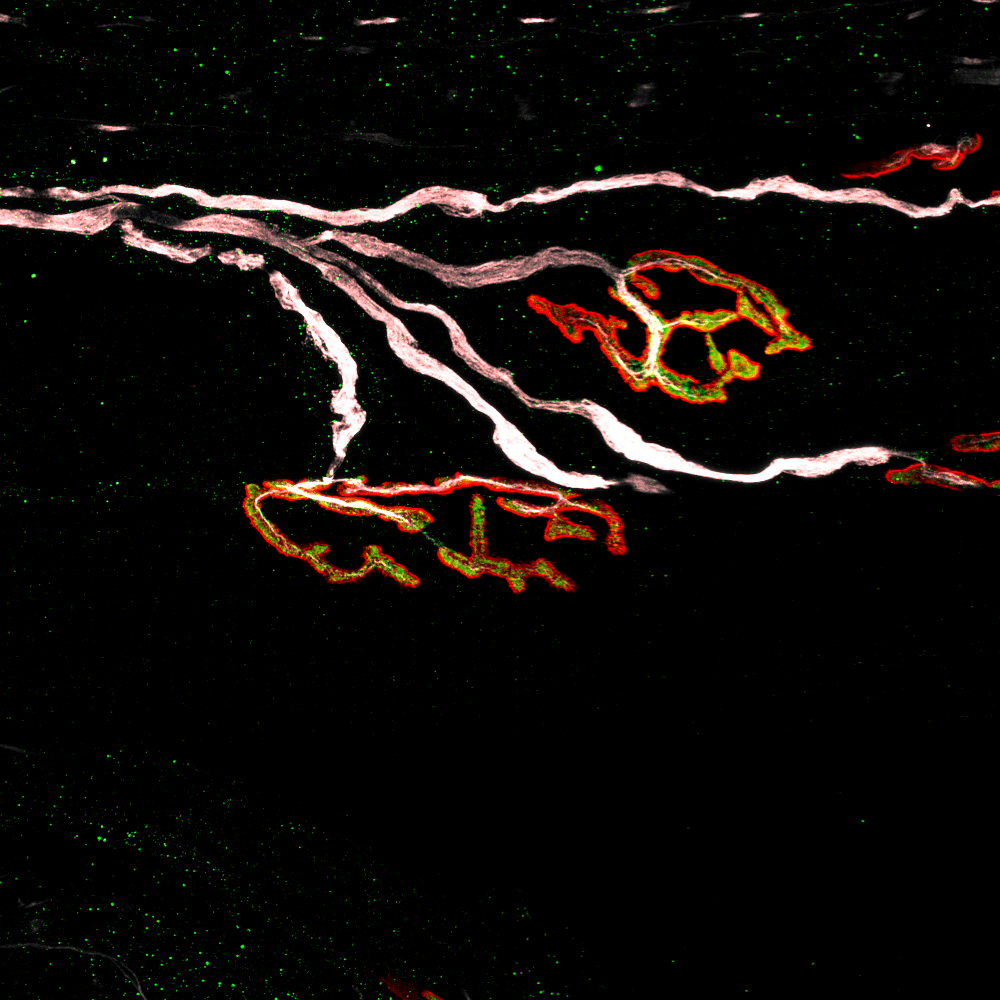

Supplement: Supplementary file 19 — Source data Fig. 3 [file 44319_2024_213_MOESM19_ESM.zip › Figure 3/3G/CKO_3464_Image 3_Airyscan Processing_Maximum intensity projection.lsm (RGB).tif]

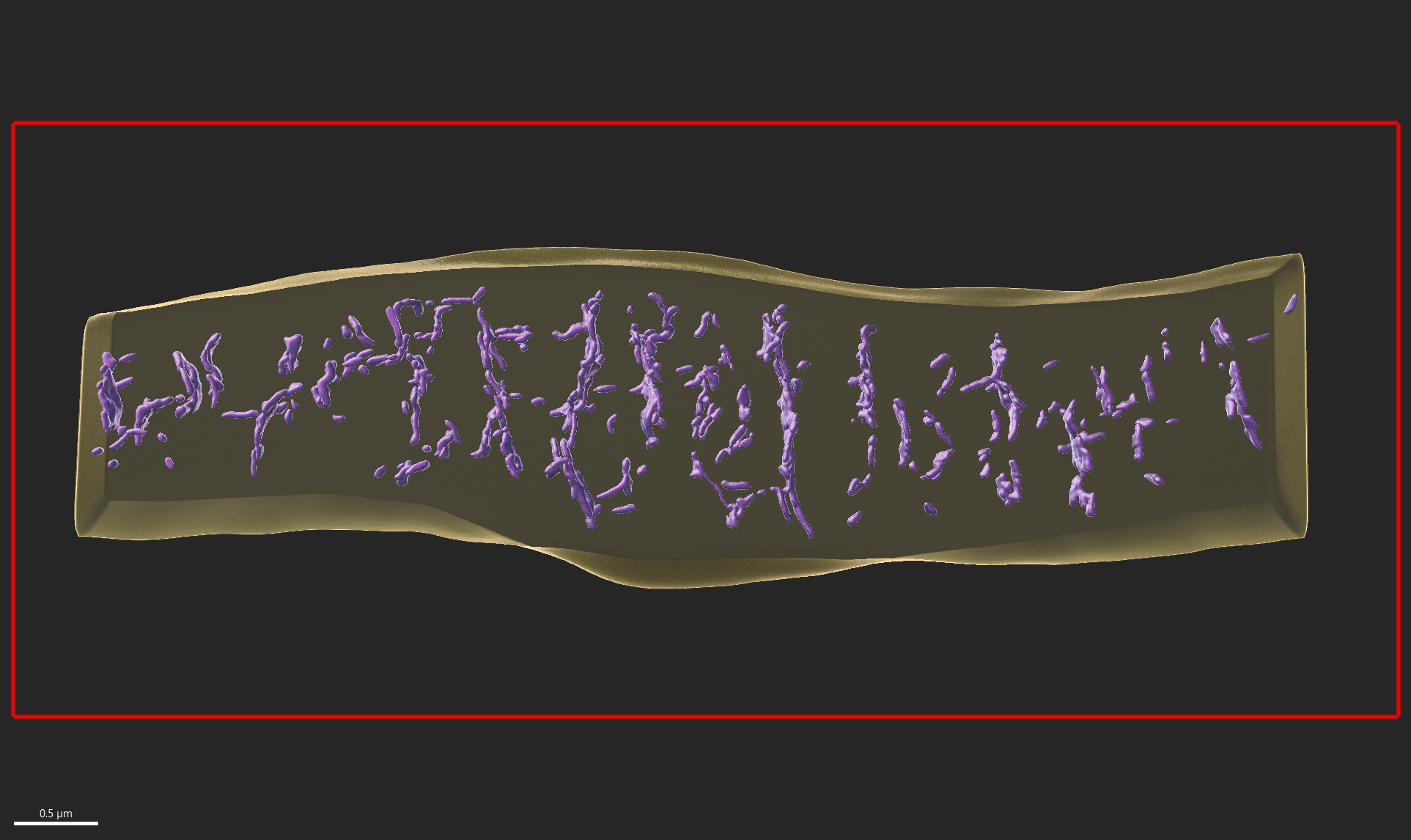

Supplement: Supplementary file 20 — Source data Fig. 4 [file 44319_2024_213_MOESM20_ESM.zip › Figure 4/4E/Labels_mut_snaps_5000x_bin2 stack 20240502 ER crop4_2024-05-07T15-38-04.630_Resize.tif]

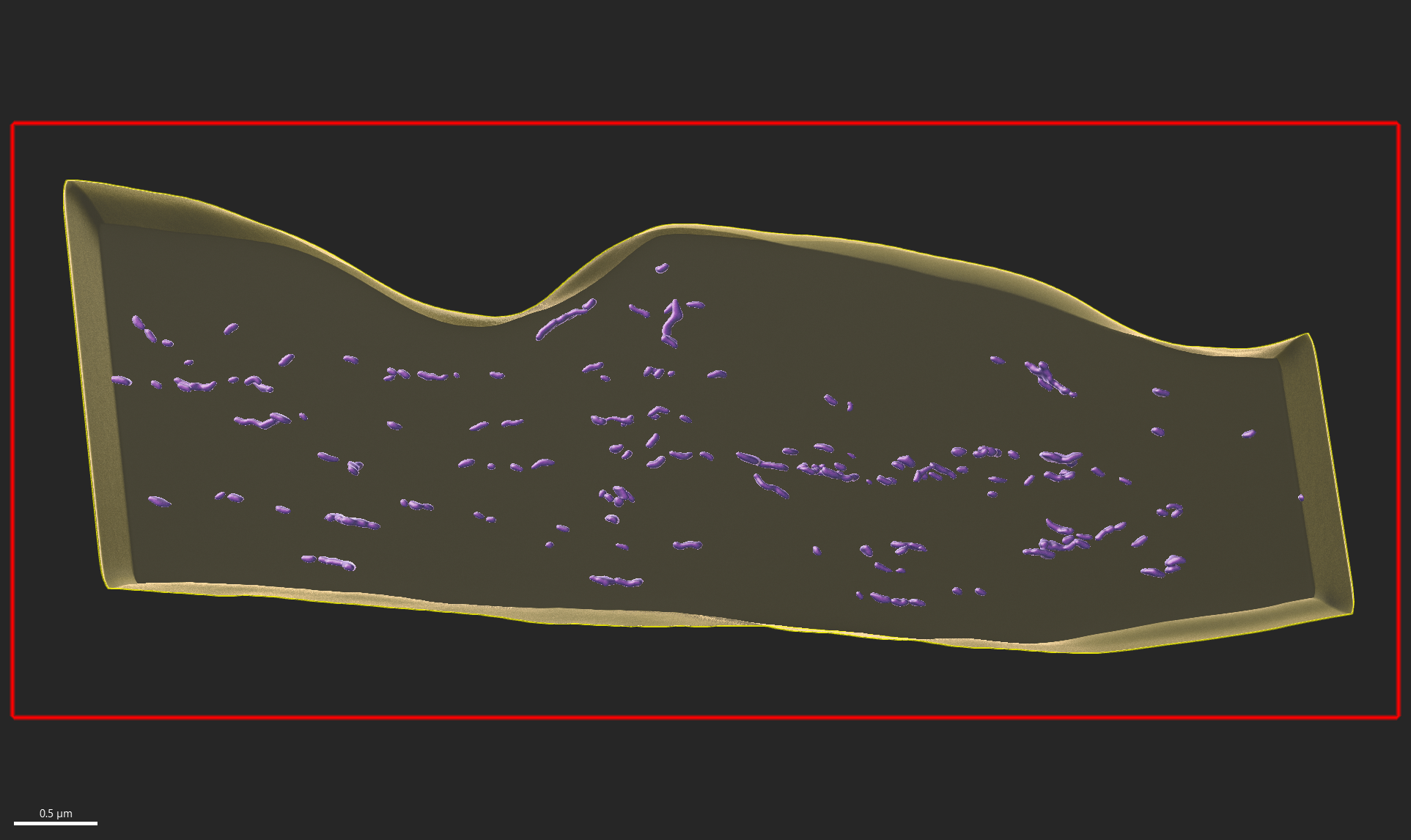

Supplement: Supplementary file 20 — Source data Fig. 4 [file 44319_2024_213_MOESM20_ESM.zip › Figure 4/4E/Labels_wt_snaps_5000x_bin2 stack 20240506 ER crop4_2024-05-07T15-37-28.623_resize.tif]

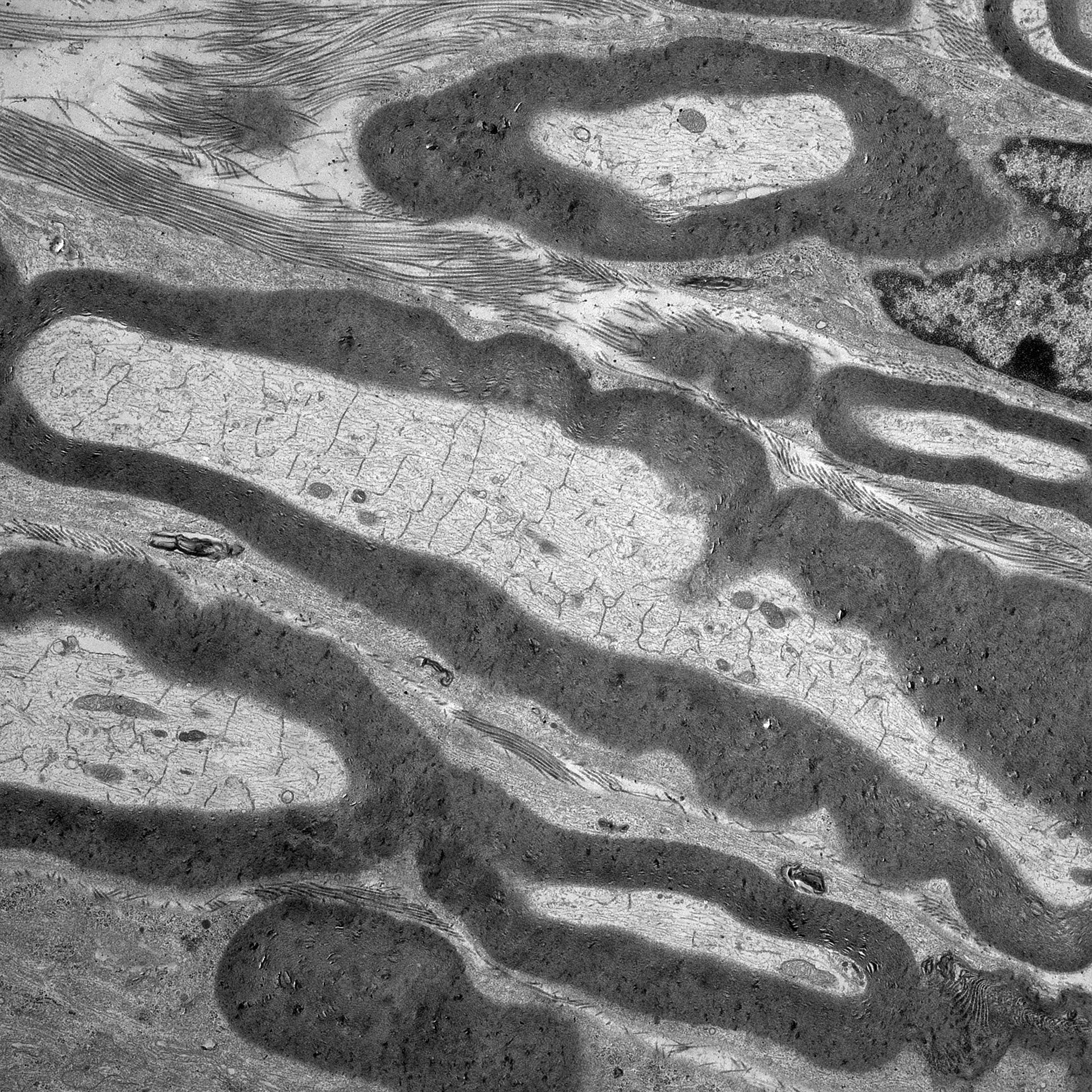

Supplement: Supplementary file 20 — Source data Fig. 4 [file 44319_2024_213_MOESM20_ESM.zip › Figure 4/4D/#2689_mouse4610_FAM134_BCKO_male_whole_nerve_2500X_0188.dm4 - C=0 no bar.tif]

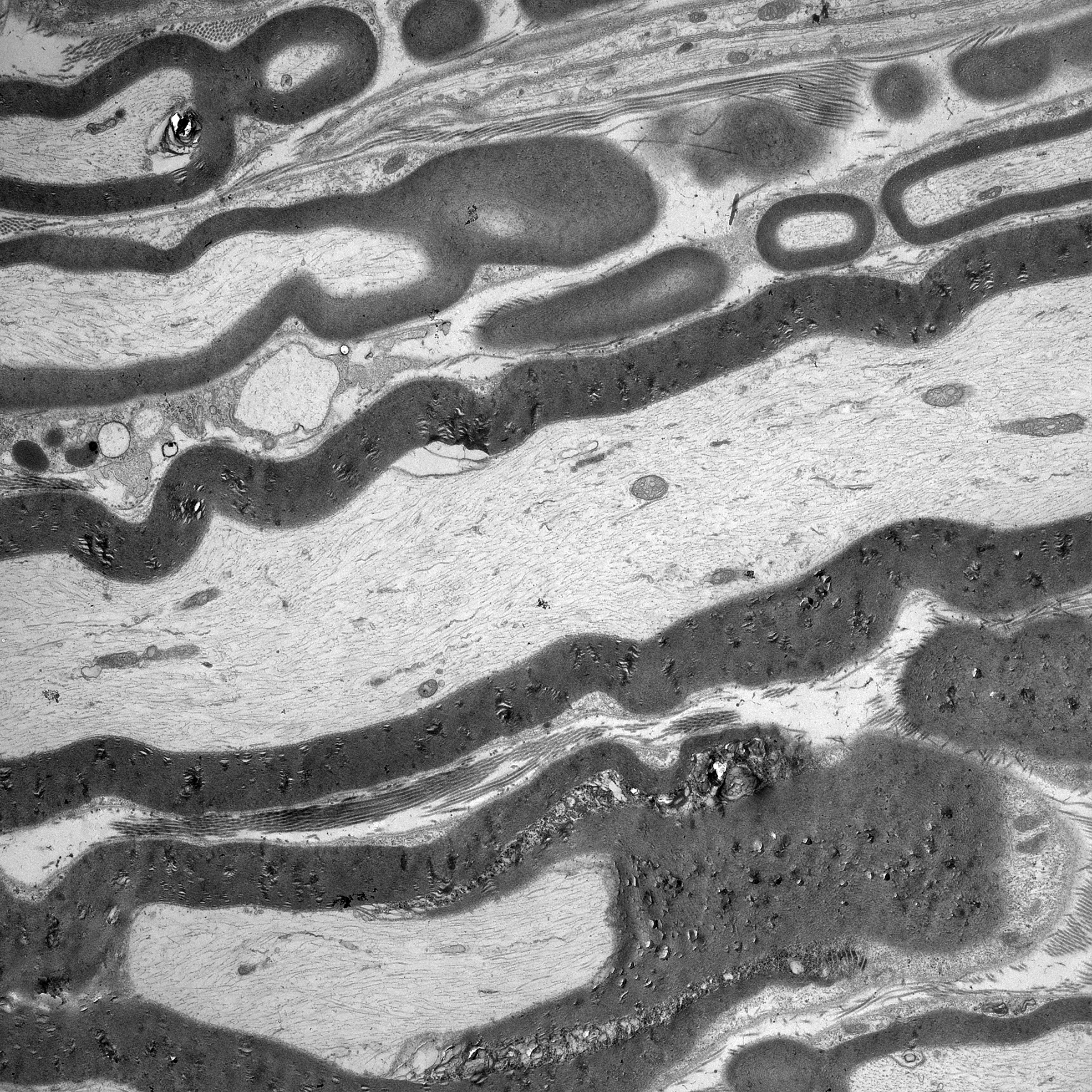

Supplement: Supplementary file 20 — Source data Fig. 4 [file 44319_2024_213_MOESM20_ESM.zip › Figure 4/4D/#2675_mouse4568_FAM134_WT_male_distal_part_2500X_0124.dm4 - C=0 no bar.tif]

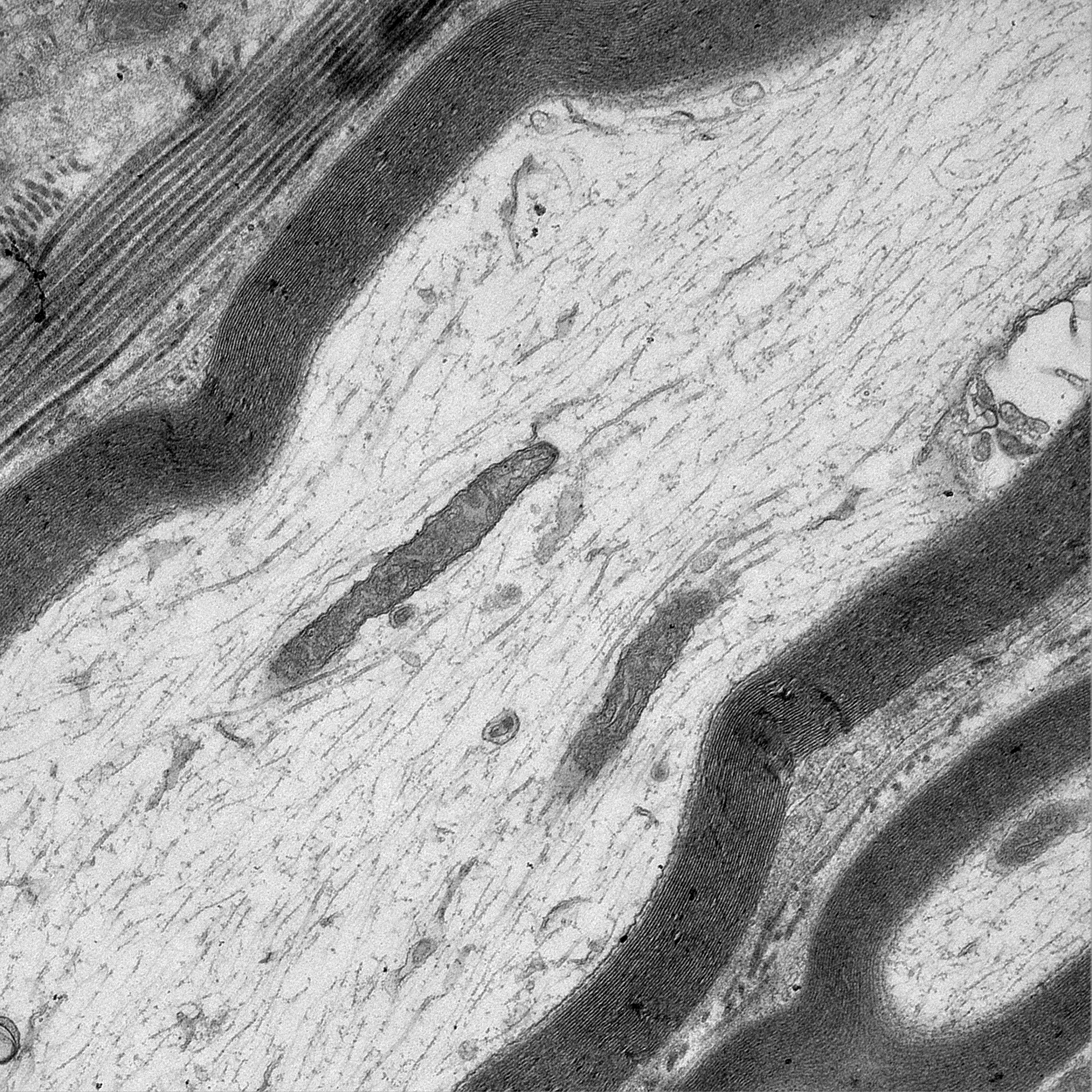

Supplement: Supplementary file 20 — Source data Fig. 4 [file 44319_2024_213_MOESM20_ESM.zip › Figure 4/4D/#2680_mouse4570_FAM134_WT_male_whole_nerve_8000X_0155.dm4 - C=0 no bar.tif]

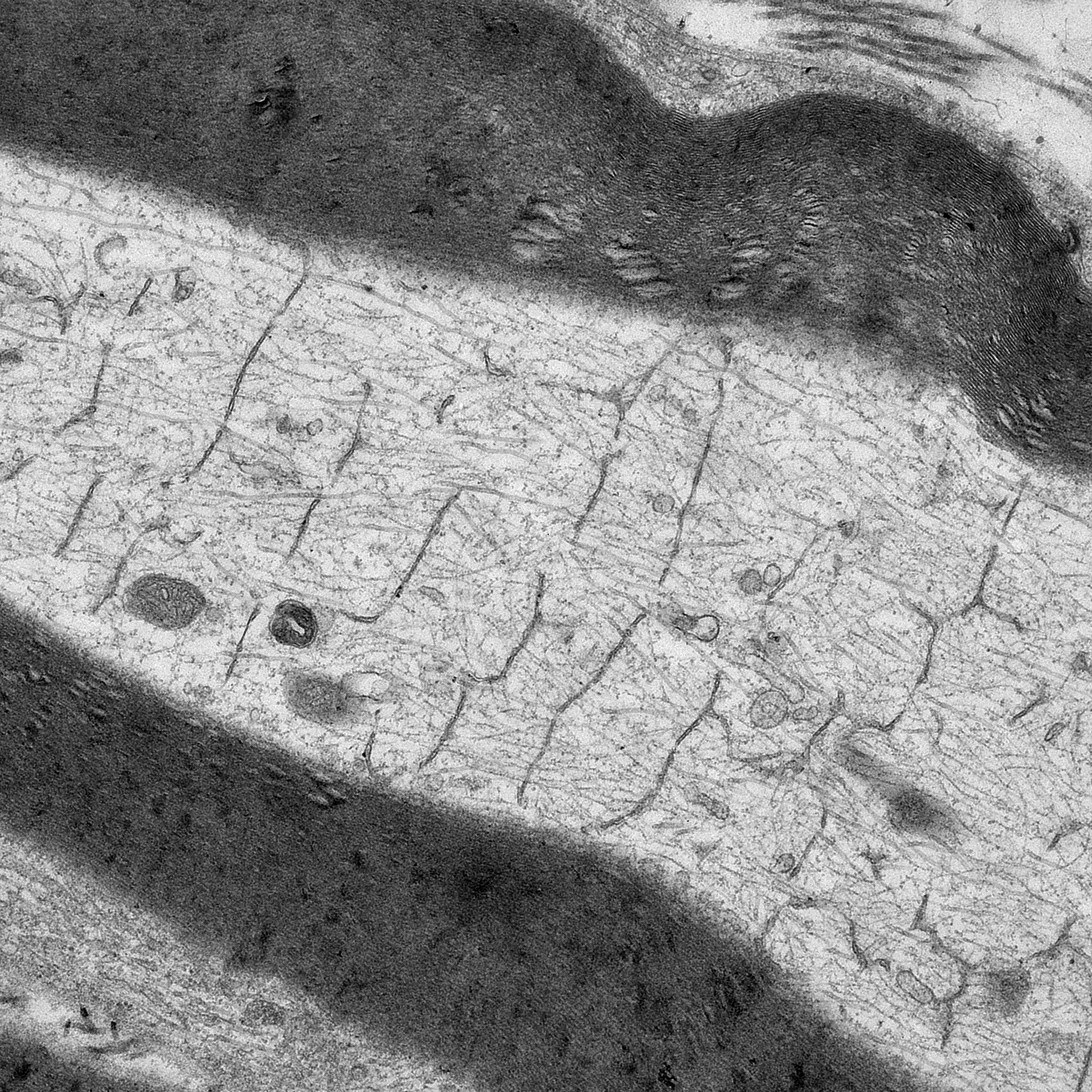

Supplement: Supplementary file 20 — Source data Fig. 4 [file 44319_2024_213_MOESM20_ESM.zip › Figure 4/4D/#2689_mouse4610_FAM134_BCKO_male_whole_nerve_8000X_0189.dm4 - C=0 no bar.tif]

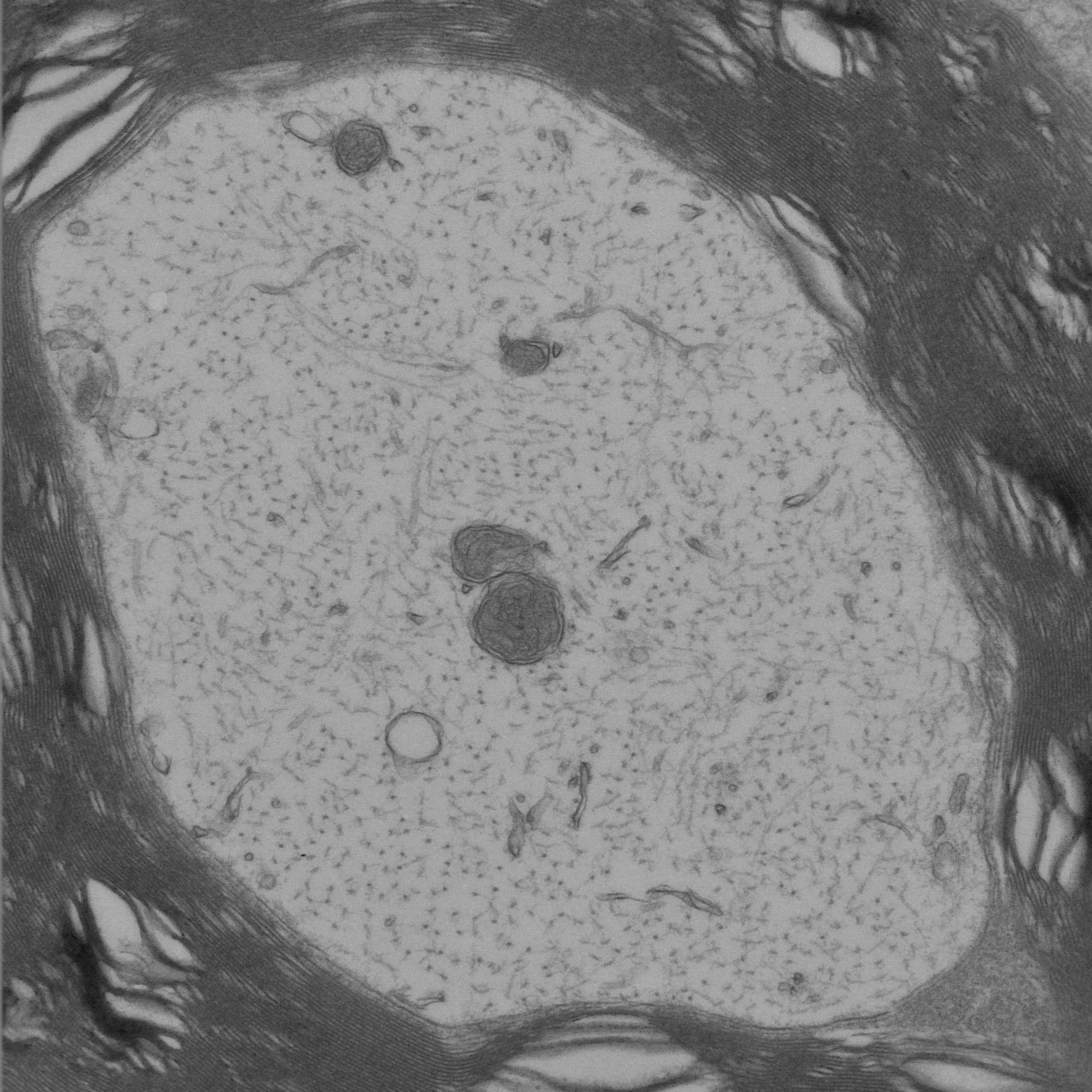

Supplement: Supplementary file 20 — Source data Fig. 4 [file 44319_2024_213_MOESM20_ESM.zip › Figure 4/4A/4 weeks 12kX_2442 b__0020 no bar.tif]

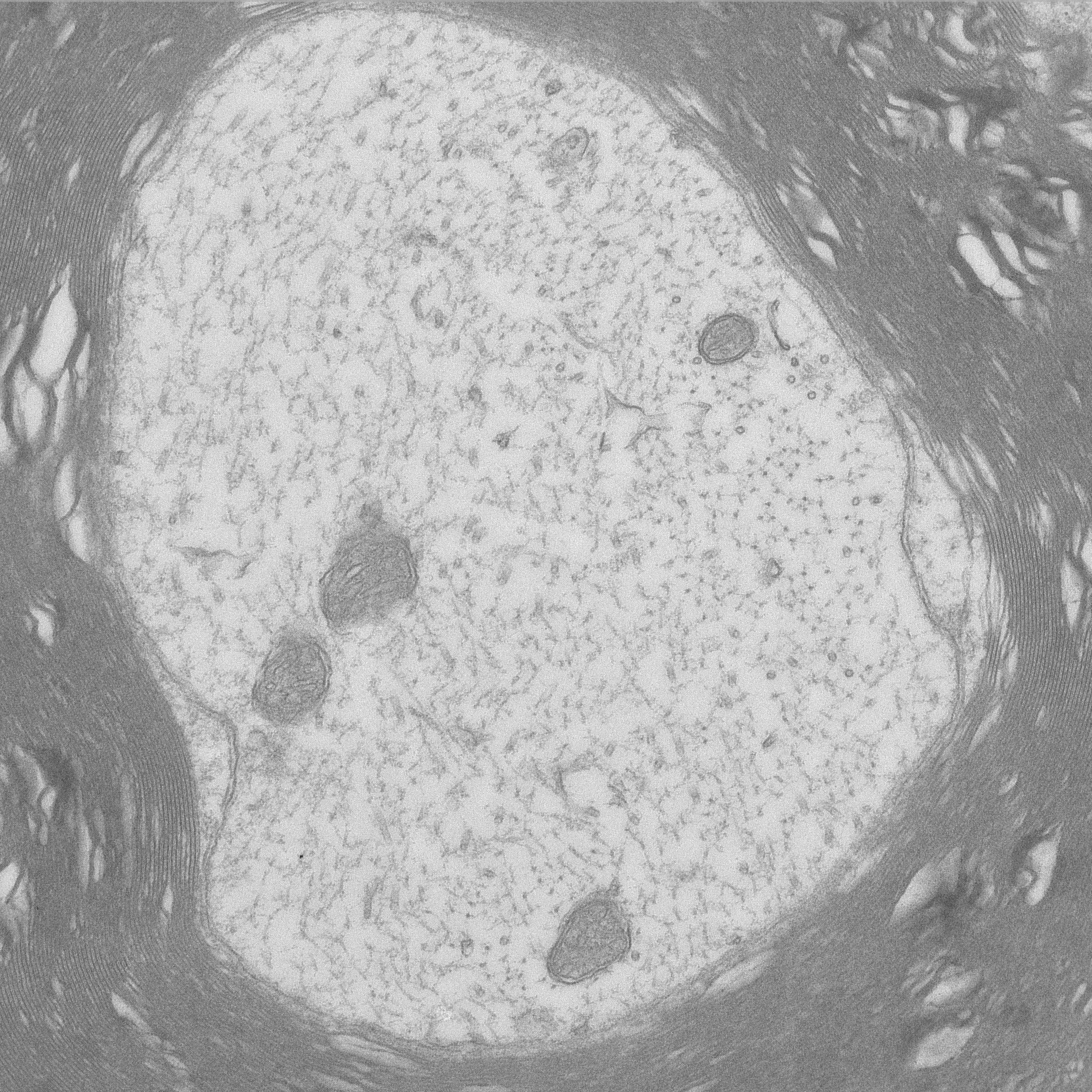

Supplement: Supplementary file 20 — Source data Fig. 4 [file 44319_2024_213_MOESM20_ESM.zip › Figure 4/4A/4 weeks 12kX_2451 wt__0023 no bar.tif]

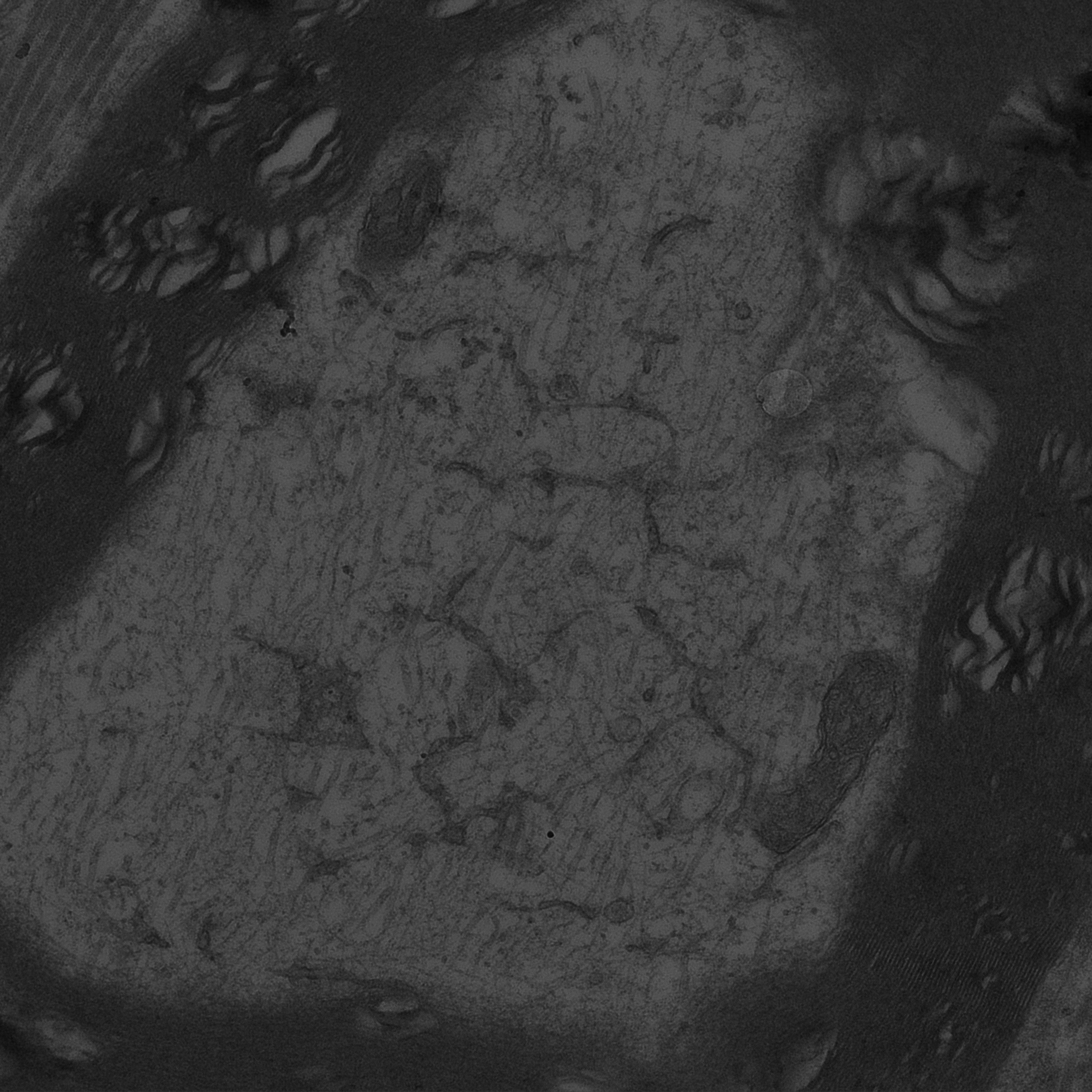

Supplement: Supplementary file 20 — Source data Fig. 4 [file 44319_2024_213_MOESM20_ESM.zip › Figure 4/4A/4 weeks 12kX_2433 bc__0001 no bar.tif]

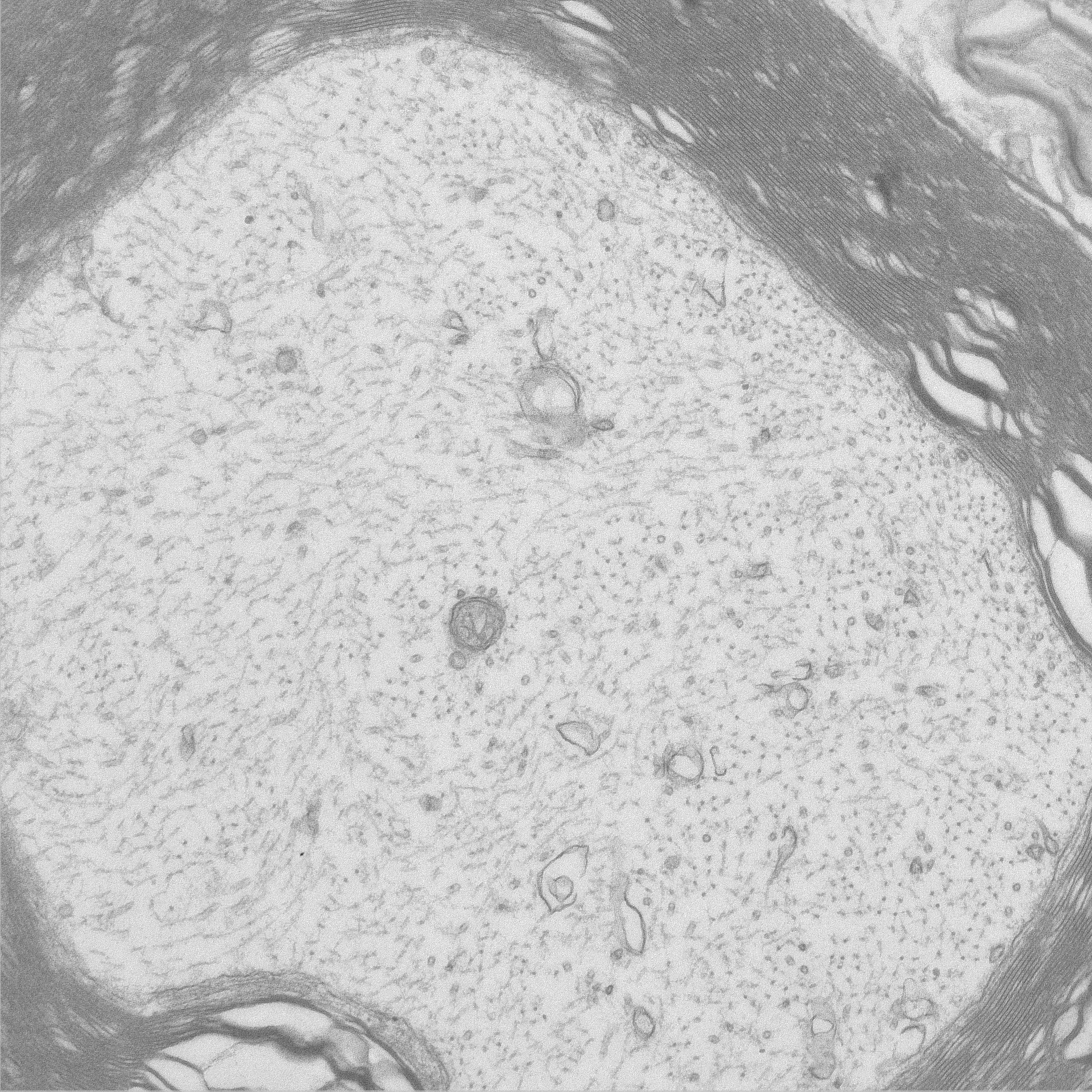

Supplement: Supplementary file 20 — Source data Fig. 4 [file 44319_2024_213_MOESM20_ESM.zip › Figure 4/4A/4 weeks 12kX_2439 c__0007 no bar.tif]

Fam134a (Brain)

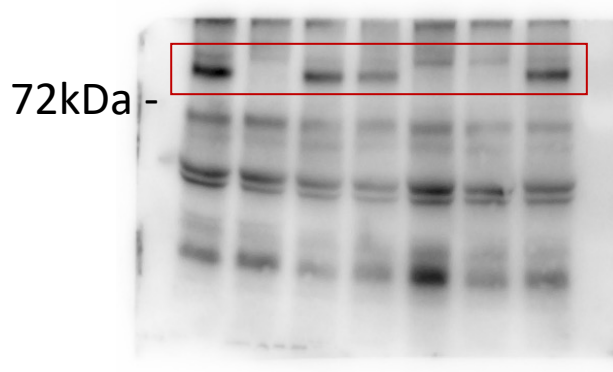

Fam134a (Spinal cord)

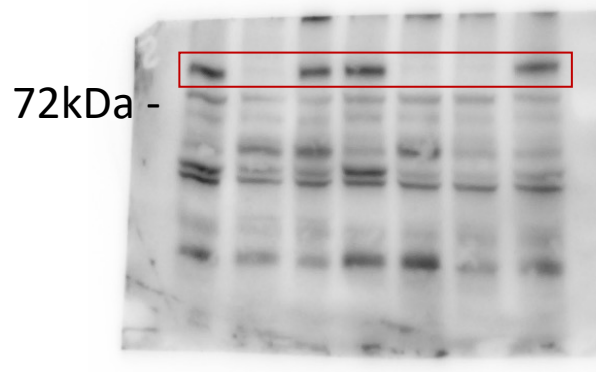

Fam134a (DRG)

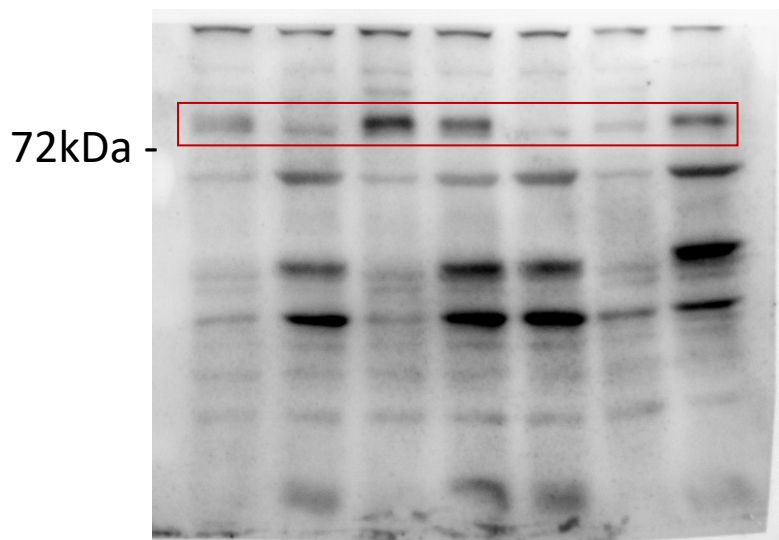

Fam134a (Sciatic nerve)

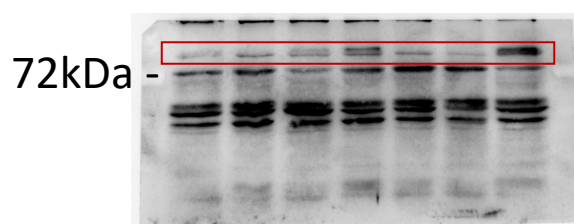

Supplement: Supplementary file 21 — Source data Fig. 5 [file 44319_2024_213_MOESM21_ESM.zip › Figure 5/5A/Fam134a.pdf]

Fam134c (Brain)

72kDa -

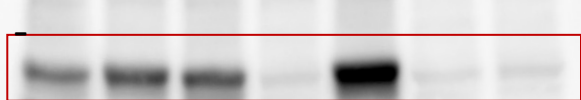

Fam134c (Spinal cord)

72kDa -

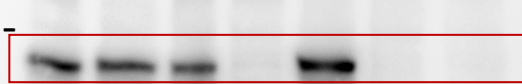

Fam134c (DRG)

72kDa -

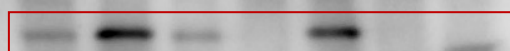

Fam134c (Sciatic nerve)

72kDa -

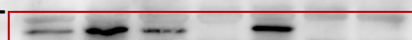

Supplement: Supplementary file 21 — Source data Fig. 5 [file 44319_2024_213_MOESM21_ESM.zip › Figure 5/5A/Fam134c.pdf]

Fam134b (Brain)

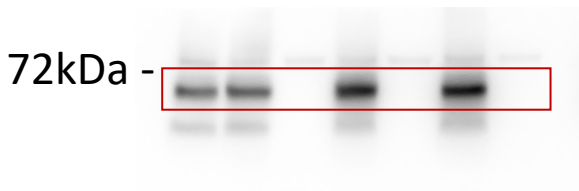

Fam134b (Spinal cord)

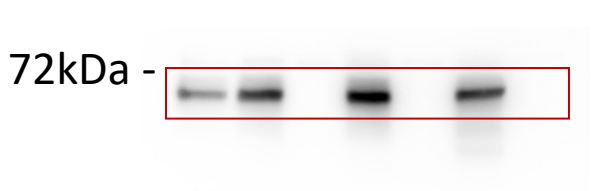

Fam134b (DRG)

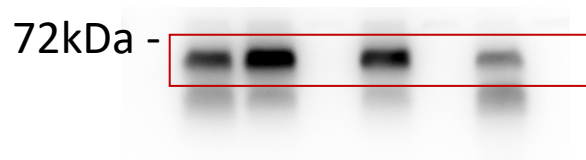

Fam134b (Sciatic nerve)

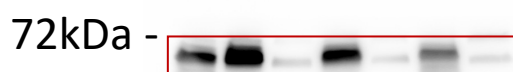

Supplement: Supplementary file 21 — Source data Fig. 5 [file 44319_2024_213_MOESM21_ESM.zip › Figure 5/5A/Fam134b.pdf]

Vinculin (Brain)

130kDa -

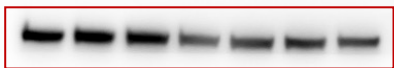

Vinculin (Spinal cord)

130kDa -

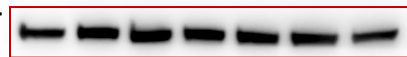

Vinculin (DRG)

130kDa -

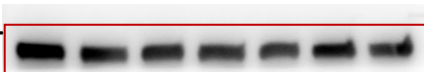

Vinculin (Sciatic nerve)

130kDa -

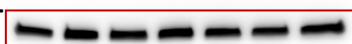

Supplement: Supplementary file 21 — Source data Fig. 5 [file 44319_2024_213_MOESM21_ESM.zip › Figure 5/5A/Vinculin (2).pdf]

Vinculin (Brain)

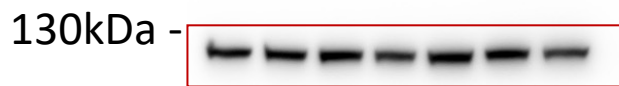

Vinculin (Spinal cord)

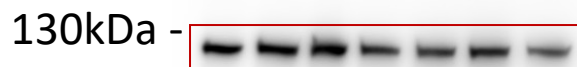

Vinculin (DRG)

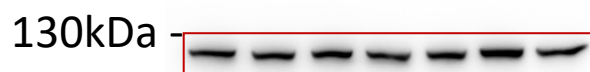

Vinculin (Sciatic nerve)

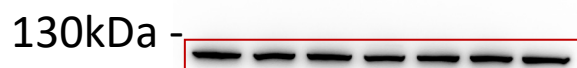

Supplement: Supplementary file 21 — Source data Fig. 5 [file 44319_2024_213_MOESM21_ESM.zip › Figure 5/5A/Vinculin (1).pdf]

### Vinculin (Reep1)

130kDa -

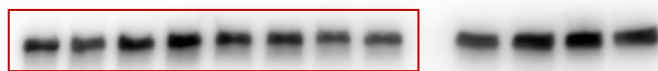

### Vinculin (Reep2)

130kDa -

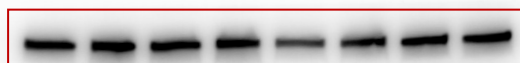

Supplement: Supplementary file 21 — Source data Fig. 5 [file 44319_2024_213_MOESM21_ESM.zip › Figure 5/5F/Vinculin (Reep1 - Reep2).pdf]

## Reep1

26kDa -

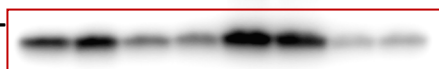

## Reep2

26kDa -

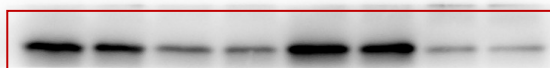

Supplement: Supplementary file 21 — Source data Fig. 5 [file 44319_2024_213_MOESM21_ESM.zip › Figure 5/5F/Reep1 - Reep2.pdf]

beta-Tubulin

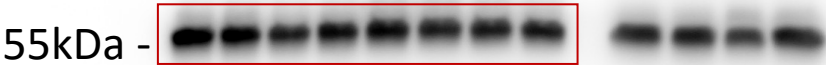

Supplement: Supplementary file 21 — Source data Fig. 5 [file 44319_2024_213_MOESM21_ESM.zip › Figure 5/5H/beta-Tubulin.pdf]

Vinculin (beta-Tubulin)

130kDa -

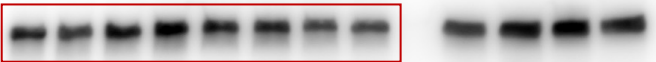

Supplement: Supplementary file 21 — Source data Fig. 5 [file 44319_2024_213_MOESM21_ESM.zip › Figure 5/5H/Vinculin (beta-Tubulin).pdf]

beta-Actin

43kDa -

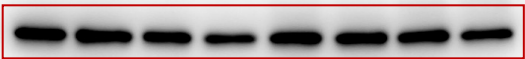

Supplement: Supplementary file 21 — Source data Fig. 5 [file 44319_2024_213_MOESM21_ESM.zip › Figure 5/5H/beta-Actin.pdf]

Vinculin (NfH – beta-Actin)

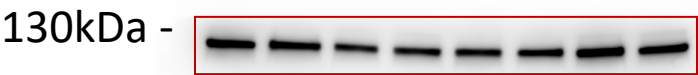

Supplement: Supplementary file 21 — Source data Fig. 5 [file 44319_2024_213_MOESM21_ESM.zip › Figure 5/5H/Vinculin (NfH ΓÇô beta-Actin) .pdf]

## Neurofilament heavy chain (NfH)

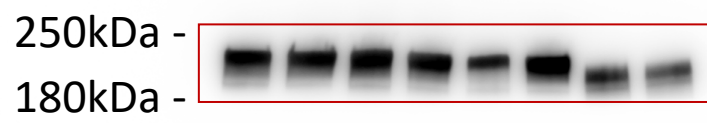

Supplement: Supplementary file 21 — Source data Fig. 5 [file 44319_2024_213_MOESM21_ESM.zip › Figure 5/5H/NfH.pdf]

## Hyperphospho NfH (SMI31)

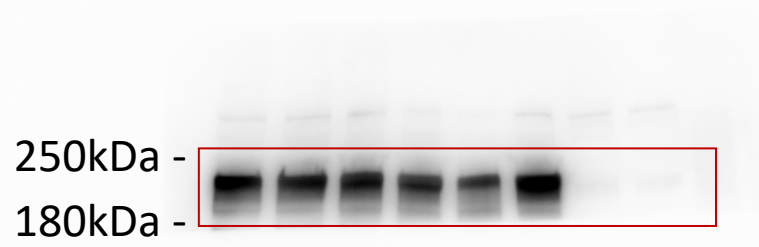

Supplement: Supplementary file 21 — Source data Fig. 5 [file 44319_2024_213_MOESM21_ESM.zip › Figure 5/5H/Hyper-P-NfH.pdf]
