# Supplementary material for: Fam134c and Fam134b shape axonal endoplasmic reticulum architecture in vivo
Source: EMBO Rep. 2024 Jul 22;25(8):25. doi: 10.1038/s44319-024-00213-7 (PMC11316074; doi:10.1038/s44319-024-00213-7)
Supplement: Supplementary file 23 — Expanded View Figures [file 44319_2024_213_MOESM23_ESM.pdf]

Expanded View Figures

Figure EV1. ***Fam134b/c* combined deletion determines a progressive neurological impairment.**

(**A**) *Fam134b/c*^{dKO} mice show reduced body weight at 15 weeks of age, as compared with the other Fam134 mutant mice. $n \geq 7$ animals/group. (**B**) Representative images of tricep and gastrocnemius muscles from WT, *Fam134b*^{KO}, *Fam134c*^{KO}, and *Fam134b/c*^{dKO} mice aged 15 weeks. Scale bar, 5 mm. (**C**) The weight of tricep and gastrocnemius muscles are massively reduced in *Fam134b/c*^{dKO} mice. $n \geq 4$ animals/group. (**D**) Representative images of 4-week-old mice with the indicated genotypes showing hindlimb clasping of *Fam134b/c*^{dKO} mice while they are suspended by the tail. The images relative to WT and *Fam134b/c*^{dKO} mice are also shown in Fig. 1G. (**E**, **F**) Open field test scores relative to total distance (**E**) and maximum speed (**F**) performed by WT, *Fam134b*^{KO}, and *Fam134c*^{KO}, and *Fam134b/c*^{dKO} mice aged 4 or 15 weeks, indicating a worsening of performance in 15-week-old *Fam134b/c*^{dKO} mice compared with the other genotypes. $n \geq 12$ animals/group. (**G**) Track plot relative to the exploration of WT, *Fam134b*^{KO}, *Fam134c*^{KO}, and *Fam134b/c*^{dKO} mice during the open field test at the indicated age, showing a dramatic decrease of *Fam134b/c*^{dKO} mice movement at 15 weeks of age. (**H**) Hot plate test indicating increased latency (s) of first hind paw pain response in *Fam134b/c*^{dKO} mice aged 4 and 15 weeks respect with the indicated genotypes. $n \geq 12$ animals/group. (**I–K**) Extracellular single-unit in vivo recordings of spinal nociceptive neurons in 4-week-old mice, measuring the spontaneous activity or firing rate (**I**), the activity evoked by mechanical stimulation (**J**), as well as the duration of evoked activity (**K**). *Fam134b/c*^{dKO} and, at a lower extent, *Fam134c*^{KO} mice exhibit an increase in all the parameters analyzed compared with the other genotypes. $n \geq 3$ animals/group. Data information: Statistical significance was determined by one-way ANOVA (**A**, **C**, **E**, **F**, **H–K**) followed by Tukey's multiple comparisons test. Data represent mean ± SEM. ns $P > 0.05$, $^{*}P < 0.05$, $^{**}P < 0.01$, $^{***}P < 0.001$, $^{****}P < 0.0001$. Statistical analysis and exact *P* values are included in the source data files. Images of behavioral tests were generated using Biorender. Source data are available online for this figure.

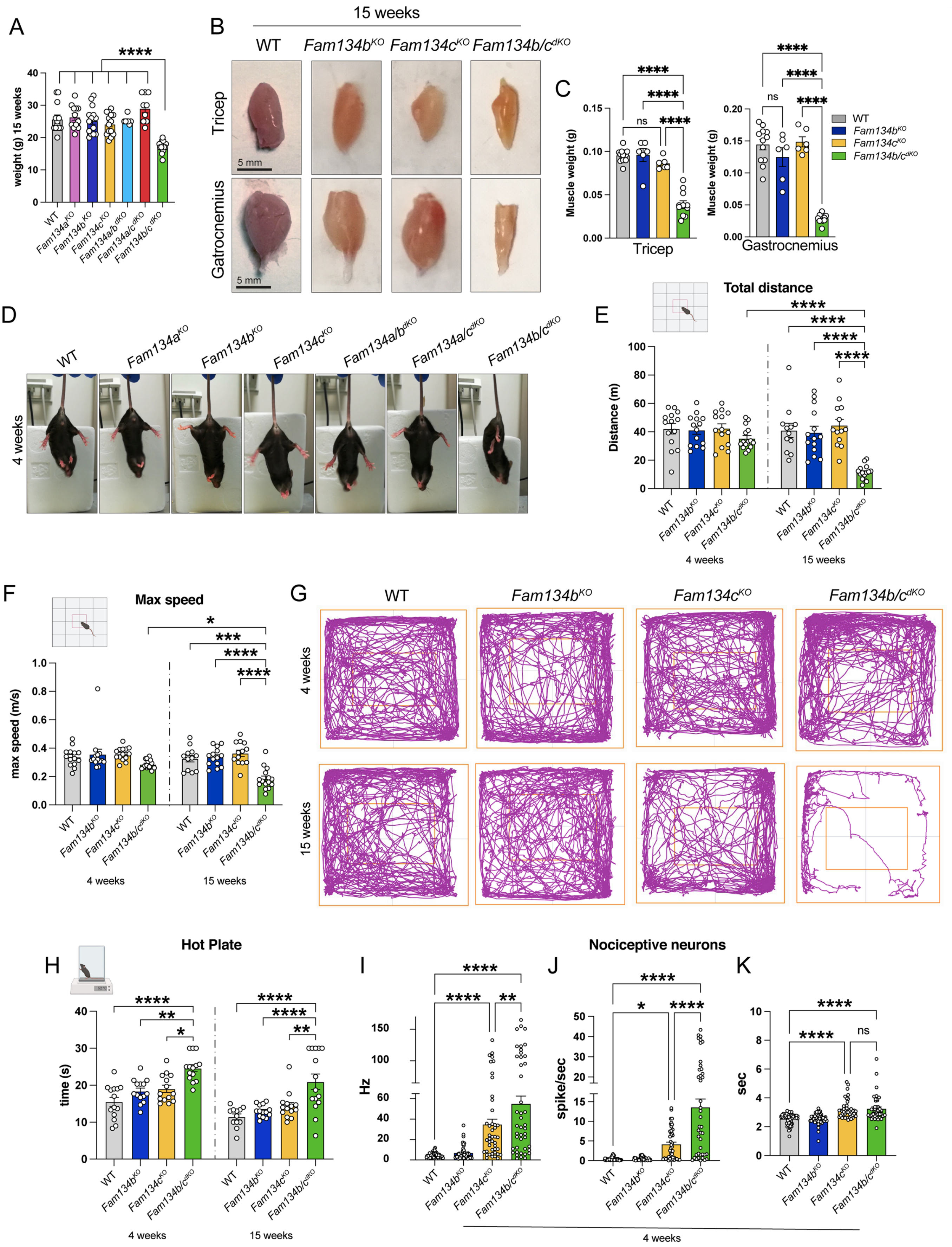
A
weight (g) 15 weeks
****
WT
Fam134a^KO
Fam134b^KO
Fam134c^KO
Fam134a/b^dKO
Fam134a/c^dKO
Fam134b/c^dKO
B
15 weeks
WT
Fam134b^KO
Fam134c^KO
Fam134b/c^dKO
Tricep
Gatrocnemius
5 mm
C
Muscle weight (g)
Tricep
Gastrocnemius
ns
****
WT
Fam134b^KO
Fam134c^KO
Fam134b/c^dKO
D
4 weeks
E
Total distance
Distance (m)
4 weeks
15 weeks
F
Max speed
max speed (m/s)
G
15 weeks
H
Hot Plate
time (s)
Nociceptive neurons
I
Hz
J
spike/sec
K
sec
4 weeks

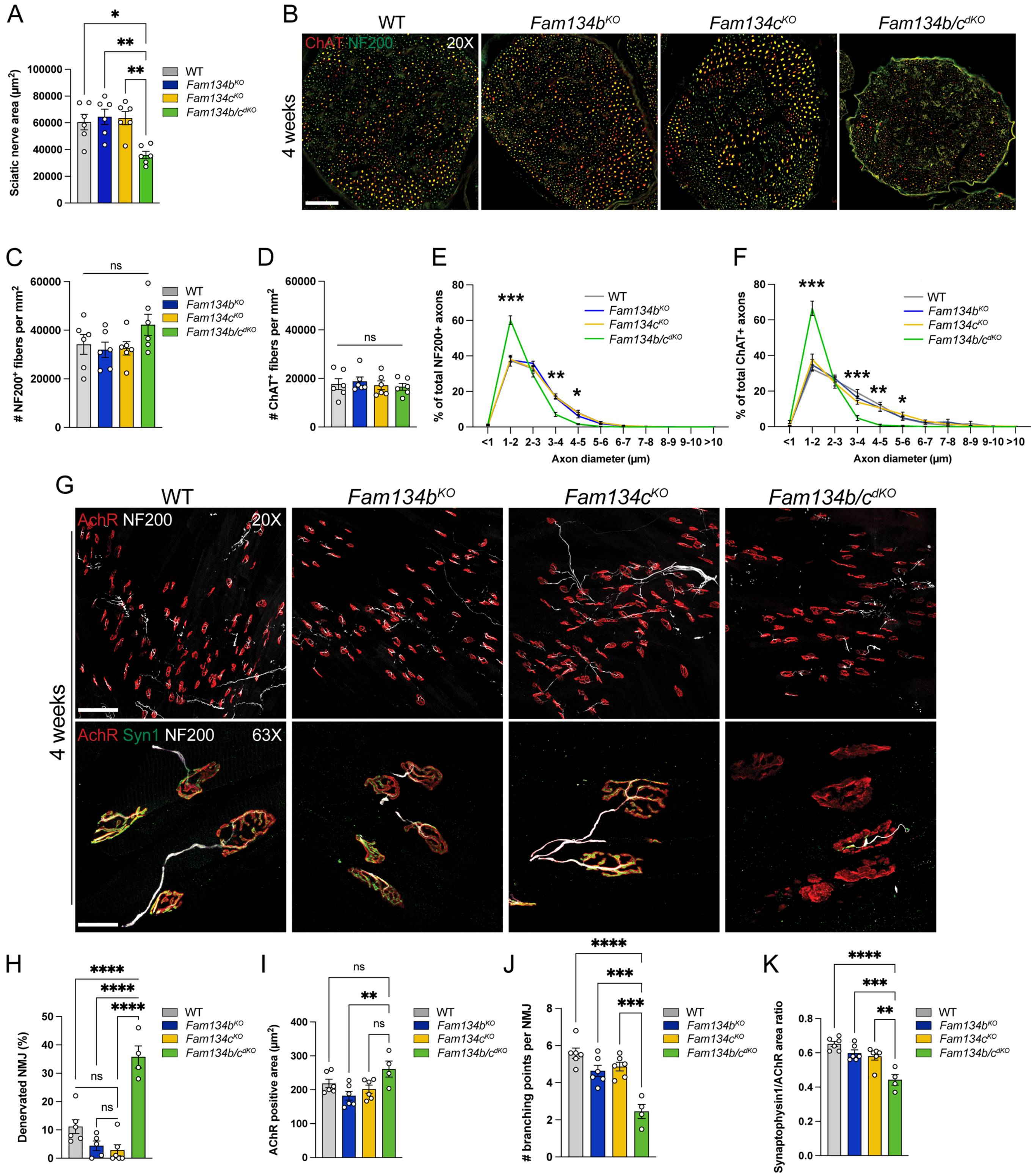
A
Sciatic nerve area (µm²)
WT
Fam134b^KO
Fam134c^KO
Fam134b/c^dKO
B
WT
Fam134b^KO
Fam134c^KO
Fam134b/c^dKO
4 weeks
ChAT NF200
20X
C
NF200⁺ fibers per mm²
ns
D
ChAT⁺ fibers per mm²
ns
E
% of total NF200+ axons
Axon diameter (µm)
F
% of total ChAT+ axons
Axon diameter (µm)
G
AchR NF200
20X
AchR Syn1 NF200
63X
H
Denervated NMJ (%)
I
AChR positive area (µm²)
J
branching points per NMJ
K
Synaptophysin1/AChR area ratio

Figure EV2. Early onset of axonal neurodegeneration and denervation of neuromuscular junctions in *Fam134b/c*^{dKO}.

(**A**) Quantification of the tibial sciatic nerve area shows a significant decrease in *Fam134b/c*^{dKO} compared with the other genotypes. (**B**) Representative immunofluorescence staining of ChAT (red) and NF200 (green) in tibial sciatic nerve sections from 4-weeks-old WT, *Fam134b*^{KO}, *Fam134c*^{KO} and *Fam134b/c*^{dKO} mice. Scale bar, 50 µm. (**C**, **D**) Number of NF200^{+} (**C**) or ChAT^{+} (**D**) axons per mm^{2} showing no difference among genotypes. (**E**, **F**) Diameter distribution of NF200^{+} (**E**) or ChAT^{+} (**F**) axons showing accumulation of smaller sized axons in *Fam134b/c*^{dKO} compared with the other genotypes. (**G**) Representative immunofluorescence staining of AchR (red), NF200 (gray), and Syn1 (green) in EDL muscle from 4-weeks-old WT, *Fam134b*^{KO}, *Fam134c*^{KO}, and *Fam134b/c*^{dKO} mice. Scale bars, 100 µm and 25 µm, respectively, in the ×20 and ×63 magnification. (**H**) *Fam134b/c*^{dKO} muscles show an increased percentage of denervated neuromuscular junctions (NMJs) compared with the other genotypes. (**I**) AchR mean positive area indicates no main changes among the different genotypes. (**J**) *Fam134b/c*^{dKO} NMJs show a decreased number of branches compared with the other genotypes. (**K**) The ratio between the Syn1 and AChR positive area is reduced in *Fam134b/c*^{dKO} NMJs compared to the other genotypes. Data information: $n \geq 4$ animals/group in all experiments. Statistical significance was determined by one-way ANOVA (**A**, **C**, **D**, **H**–**K**) or two-way ANOVA (**E**, **F**), followed by Tukey's multiple comparisons test. Data represent mean ± SEM. ns $P > 0.05$, *$P < 0.05$, **$P < 0.01$, ***$P < 0.001$, ****$P < 0.0001$. Statistical analysis and exact *P* values are included in the source data files. Source data are available online for this figure.

© The Author(s)

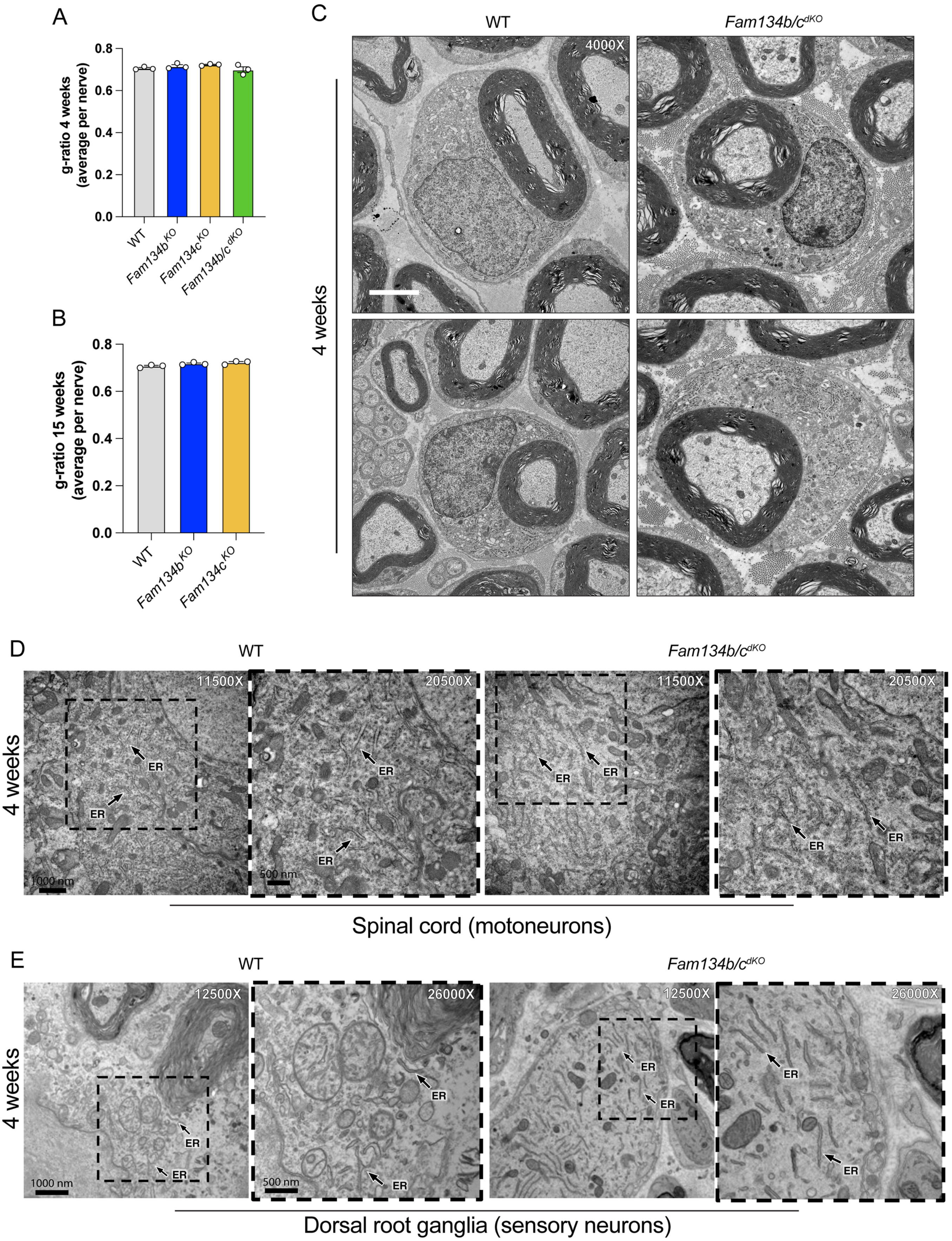
A
g-ratio 4 weeks (average per nerve)
0.8
0.6
0.4
0.2
0.0
WT
Fam134b^KO
Fam134c^KO
Fam134b/c^dKO
B
g-ratio 15 weeks (average per nerve)
0.8
0.6
0.4
0.2
0.0
WT
Fam134b^KO
Fam134c^KO
C
WT
Fam134b/c^dKO
4000X
4 weeks
D
WT
Fam134b/c^dKO
11500X
20500X
11500X
20500X
ER
4 weeks
1000 nm
500 nm
Spinal cord (motoneurons)
E
WT
Fam134b/c^dKO
12500X
26000X
12500X
26000X
ER
4 weeks
1000 nm
500 nm
Dorsal root ganglia (sensory neurons)

Figure EV3. Schwann cell, motoneuron, and sensory neuron soma show no ultrastructural alteration in *Fam134b/c*^{dKO} mice.

(**A**, **B**) Quantification of the g-ratio of WT, *Fam134b*^{KO}, *Fam134c*^{KO}, and *Fam134b/c*^{dKO} myelinated axons from tibial nerves aged 4 (**A**) and 15 (**B**) weeks. $n = 3$ animals/group. Data represent mean ± SEM. (**C**) Representative electron micrographs showing Schwann cells in WT and *Fam134b/c*^{dKO} sciatic nerve aged 4 weeks. Scale bar, 2 µm. (**D**) Representative electron micrographs of motoneurons in the ventral horn of the lumbar spinal cord from WT and *Fam134b/c*^{dKO} mice aged 4 weeks. (**E**) Representative electron micrographs of neurons in lumbar DRG from WT and *Fam134b/c*^{dKO} mice aged 4 weeks. Scale bars, 1000 nm or 500 nm in the insets. Source data are available online for this figure.

 © The Author(s)

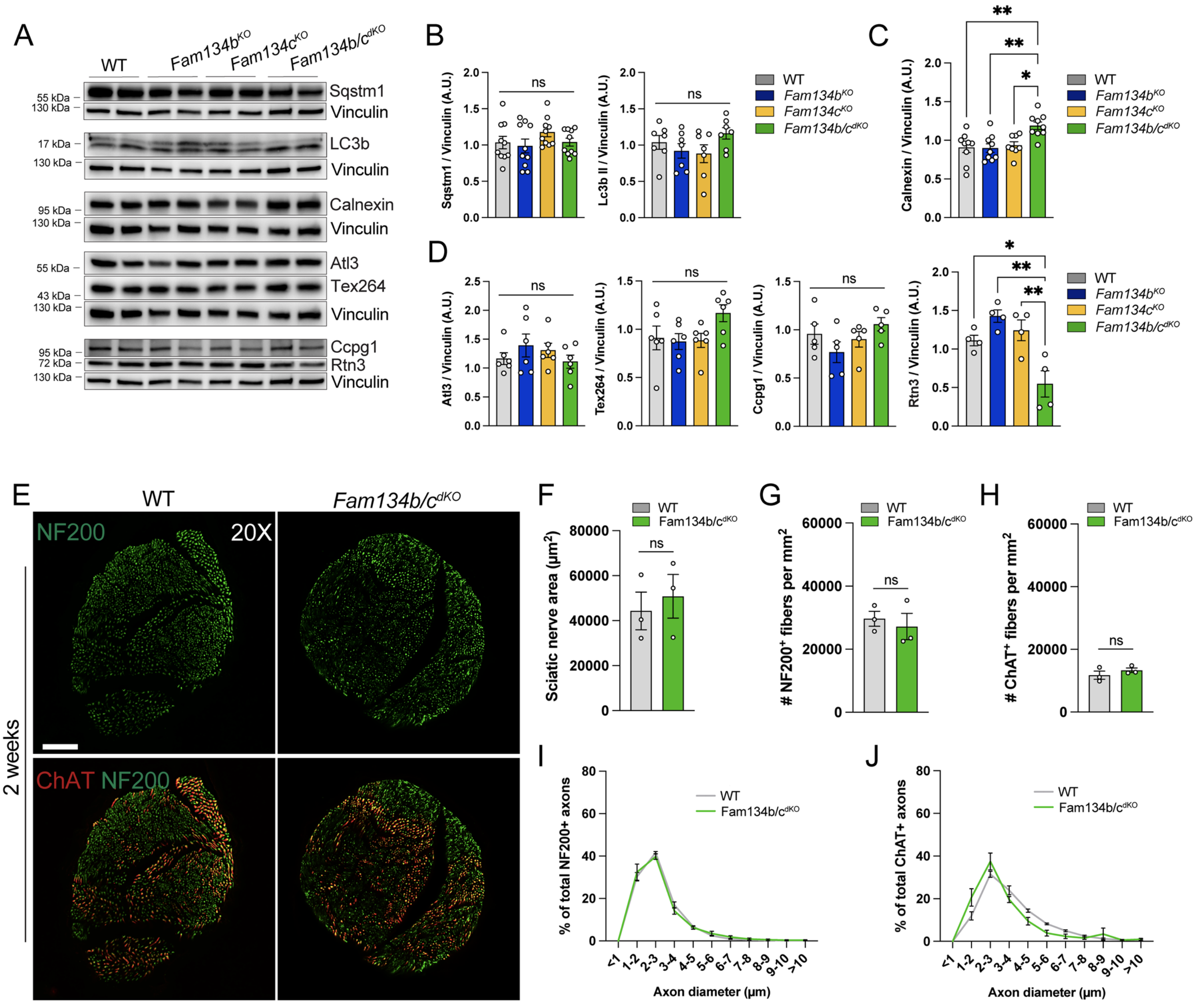

Figure EV4. Autophagy and histology of sciatic nerve show no major alterations in 2-week-old *Fam134b/c^dKO* mice.

(**A**) Western blot analysis showing protein expression of autophagy markers (Sqstm1/p62 and Lc3b), the ER marker Calnexin, and ER-phagy receptors (Atl3, Tex264, Ccpg1, and Rtn3) in sciatic nerve of 2-week-old mice with the indicated genotypes. (**B–D**) Protein level quantification of autophagy markers (**B**), Calnexin (**C**), and ER-phagy receptors (**D**). Normalized to vinculin. $n \geq 4$ animals/group. (**E**) Representative immunofluorescence staining of ChAT (red) and NF200 (green) in tibial sciatic nerve sections of WT and *Fam134b/c^dKO* mice aged 2 weeks. Scale bar, 50 µm. (**F**) Quantification of the tibial sciatic nerve area showing no difference between WT and *Fam134b/c^dKO* mice. (**G–J**) Morphometric analysis of axons shows no alterations between WT and *Fam134b/c^dKO* mice either in both the number of NF200⁺ (**G**) and ChAT⁺ axons (**H**) or in their diameter distribution (**I**, **J**). $n = 3$ animals/group. Data information: Statistical significance was determined by one-way ANOVA (**B–D**) followed by Tukey's multiple comparisons test or Student's *t*-test (**F–H**). Data represent mean ± SEM. ns $P > 0.05$, *$P < 0.05$, **$P < 0.01$. Statistical analysis and exact p-values are included in the source data files. Source data are available online for this figure.

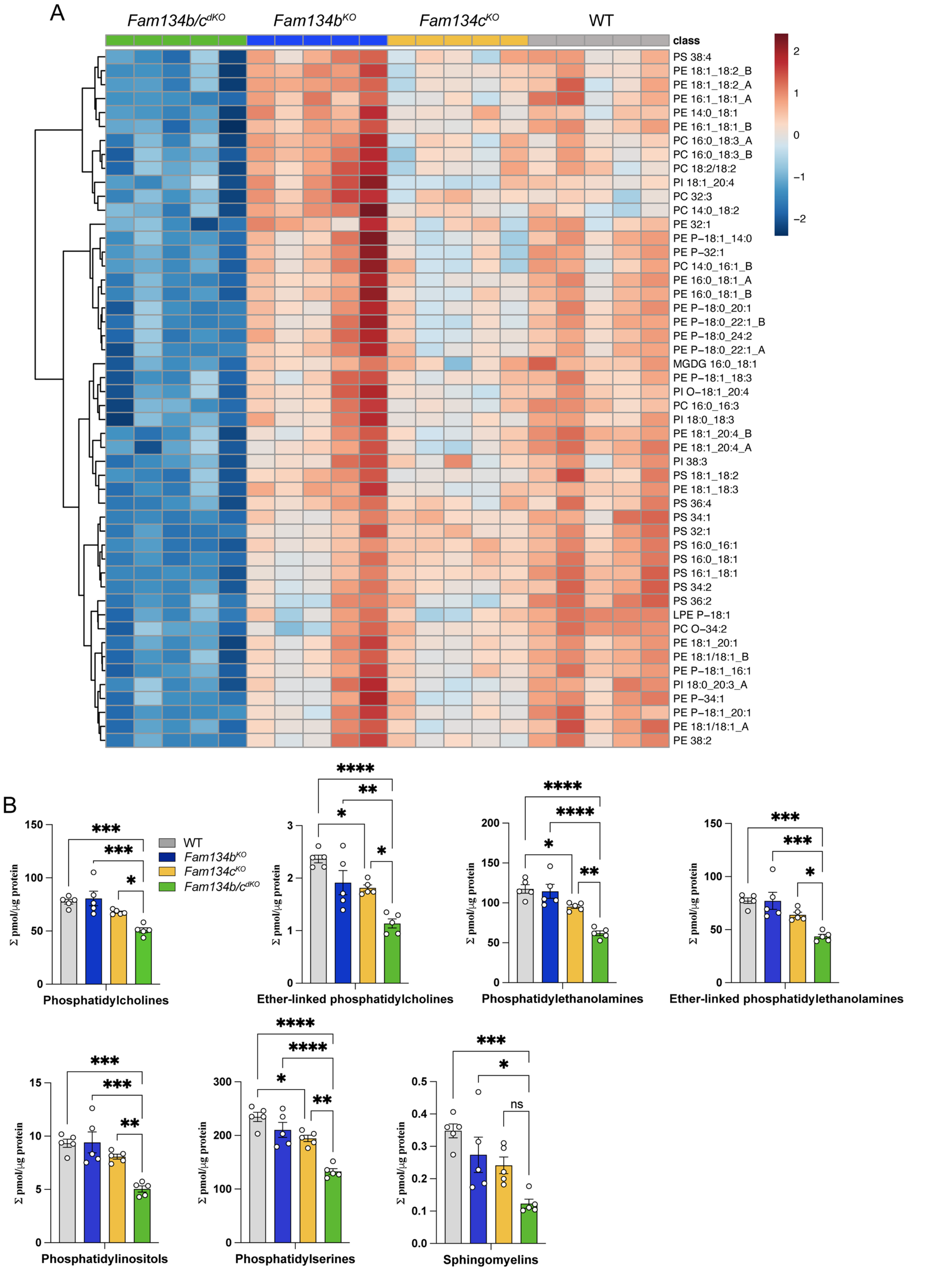
A
Fam134b/c dKO
Fam134b KO
Fam134c KO
WT
class
PS 38:4
PE 18:1_18:2_B
PE 18:1_18:2_A
PE 16:1_18:1_A
PE 14:0_18:1
PE 16:1_18:1_B
PC 16:0_18:3_A
PC 16:0_18:3_B
PC 18:2/18:2
PI 18:1_20:4
PC 32:3
PC 14:0_18:2
PE 32:1
PE P–18:1_14:0
PE P–32:1
PC 14:0_16:1_B
PE 16:0_18:1_A
PE 16:0_18:1_B
PE P–18:0_20:1
PE P–18:0_22:1_B
PE P–18:0_24:2
PE P–18:0_22:1_A
MGDG 16:0_18:1
PE P–18:1_18:3
PI O–18:1_20:4
PC 16:0_16:3
PI 18:0_18:3
PE 18:1_20:4_B
PE 18:1_20:4_A
PI 38:3
PS 18:1_18:2
PE 18:1_18:3
PS 36:4
PS 34:1
PS 32:1
PS 16:0_16:1
PS 16:0_18:1
PS 16:1_18:1
PS 34:2
PS 36:2
LPE P–18:1
PC O–34:2
PE 18:1_20:1
PE 18:1/18:1_B
PE P–18:1_16:1
PI 18:0_20:3_A
PE P–34:1
PE P–18:1_20:1
PE 18:1/18:1_A
PE 38:2
2
1
0
−1
−2
B
WT
Fam134b KO
Fam134c KO
Fam134b/c dKO
Σ pmol/µg protein
Phosphatidylcholines
Ether-linked phosphatidylcholines
Phosphatidylethanolamines
Ether-linked phosphatidylethanolamines
Phosphatidylinositols
Phosphatidylserines
Sphingomyelins
ns

 © The Author(s)

Figure EV5. Altered lipidome profile in *Fam134b/c*^{dKO} sciatic nerve.

(**A**) Heatmap reporting the top 50 statistically significant lipids and comparing WT, *Fam134b*^{KO}, *Fam134c*^{KO}, and *Fam134b/c*^{dKO}. The log2 relative lipid quantity scale is depicted on the top right. (**B**) Lipid concentration in WT, *Fam134b*^{KO}, *Fam134c*^{KO}, and *Fam134b/c*^{dKO} sciatic nerves grouped in lipid subclasses. Lipid quantity is expressed in pmol and normalized to µg of total protein. $n = 5$ animals/group, 2 replicates/animal. Statistical significance was determined by one-way ANOVA followed by Tukey's multiple comparisons test. Data represent mean ± SEM. ns $P > 0.05$, *$P < 0.05$, **$P < 0.01$, ***$P < 0.001$, ****$P < 0.0001$. Statistical analysis and exact *P* values are included in the source data files. Source data are available online for this figure.

© The Author(s)
